# Supplementary material for: Study of Dispersed Repeats in the Cyanidioschyzon merolae Genome
Source: Int J Mol Sci. 2024 Apr 18;25(8):4441. doi: 10.3390/ijms25084441 (PMC11050394; doi:10.3390/ijms25084441)
Supplement: Supplementary file 1 [file ijms-25-04441-s001.zip › ijms-2902088-supplementary/supplement/s1/readme.docx]

1.Files cyanidio_repeats_dir.csv and cyanidio_repeats_inv.csv contain data on dispersed repeats in the C.merolae genome - 17,734 and 16,204 pieces, respectively. Files have the delimited text format and, thus, can be exported to the internal format of spreadsheets and DBMS. The first line shows headers indicating the data structure; each line contains the data for one repeat (separated by semicolons):

- - *chromosome number (chromosome)*
  - *left coordinate in the chromosome (left)*
  - *right coordinate in the chromosome (right)*
  - *statistical significance Z (z)*
  - *family number (class_num)*
  - *family profile sequence (v1)*
  - *repeat sequence (v2).*

2.The number of chromosomes is 21 (the 21st corresponds to the chloroplast DNA). If sequences of the repeat family and that of the repeat are written under each other, they represent an alignment. The family sequence reflects the position number of the family profile; the insertion corresponds to number 0. In the repeat sequence, insertions are marked by asterisks.​

3.File freq_consensus.txt contains symbolic consensuses for all repeat families. Field FAM shows the number of the repeat family and IDL shows the length of the consensus.

4.File similarity_Gypsy_Copia.txt contains some similarities between repeats from *Q1* and *Q2* sets. File format corresponds blastn output format. Identifiers of compared sequencies generated by us as following rule:

*cyan_<chromosome_number>_<left_coordinate>_<rigth_coordinate>_<direction>_<LTR/Copia | LTR/Gypsy>*

Last part of the indentifier is a class of the ADR: ‘LTR/Copia’ or ‘LTR/Gypsy’, dir – corresponds DNA strand ‘+’ - forward or ‘-‘ – reverse.

5. Files circos_plot_<*n*>.pdf, where *n* is the family number from 1 to 20, illustrate the location of dispersed repeats from different families across *C. merolae* chromosomes. The outer circle shows the chromosomes, the length of the sector of the circle is proportional to the size of the chromosome. The middle circle shows the localization of dispersed repeats on the forward DNA strand, the inner circle - on the reverse strand. The colors of the strokes correspond to the Z value for the repeat. Colormap blue-green-yellow is used in the increasing Z direction.
