# Supplementary figures and images for: Study of Dispersed Repeats in the Cyanidioschyzon merolae Genome
Source: Int J Mol Sci. 2024 Apr 18;25(8):4441. doi: 10.3390/ijms25084441 (PMC11050394; doi:10.3390/ijms25084441)

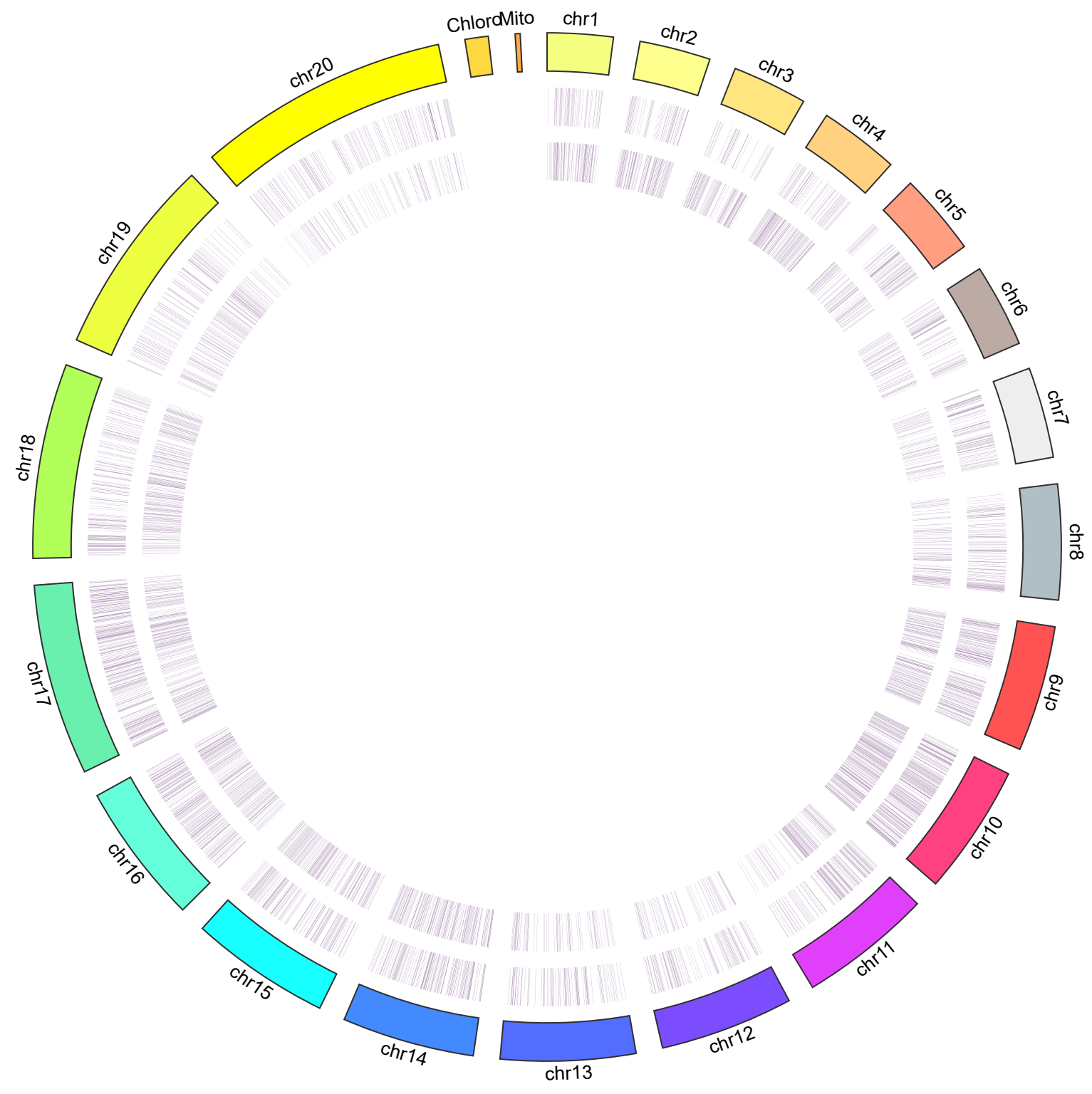

Supplement: Supplementary file 1 [file ijms-25-04441-s001.zip › ijms-2902088-supplementary/supplement/s1/circos_plot_1.pdf]

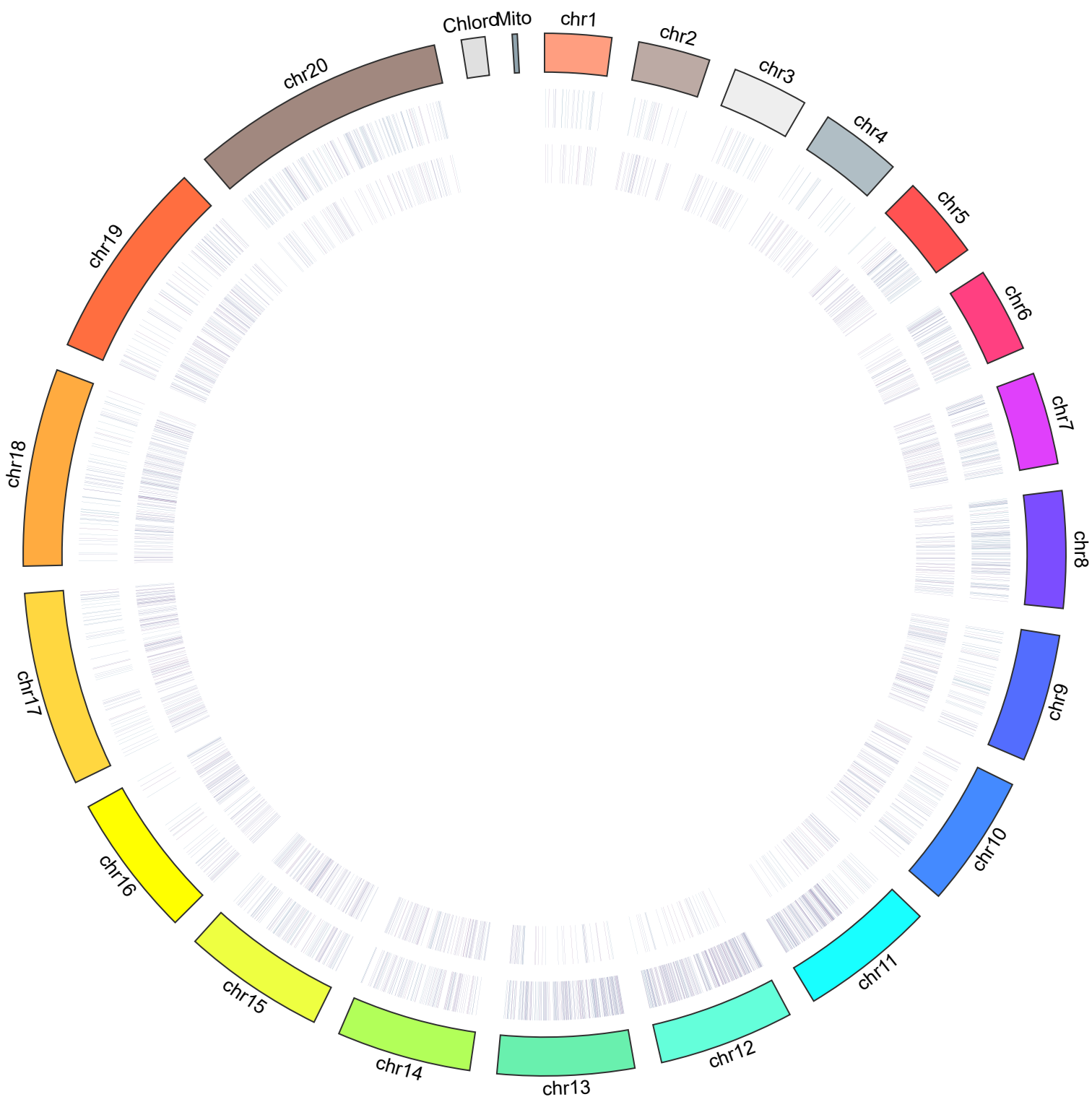

Supplement: Supplementary file 1 [file ijms-25-04441-s001.zip › ijms-2902088-supplementary/supplement/s1/circos_plot_10.pdf]

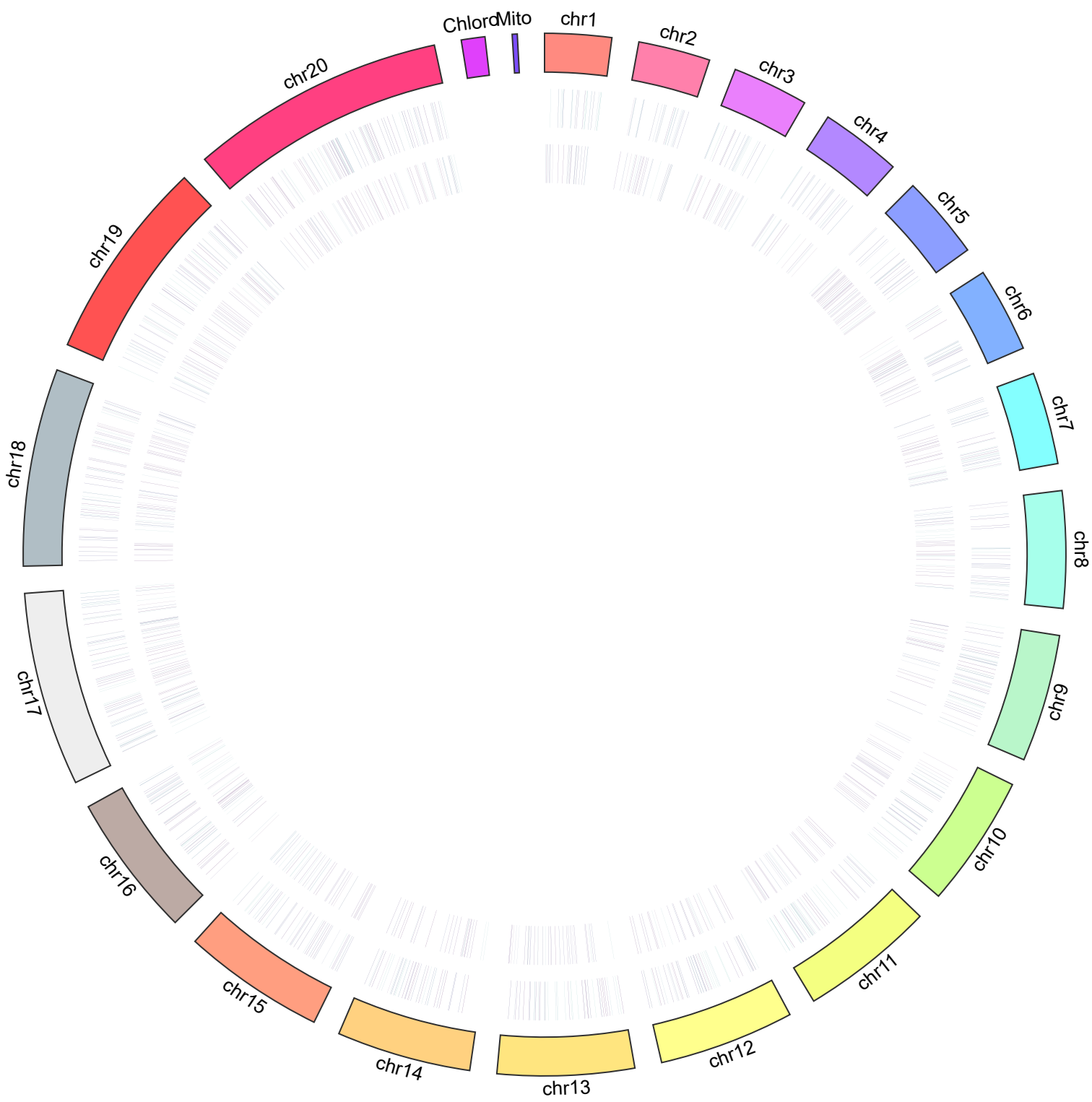

Supplement: Supplementary file 1 [file ijms-25-04441-s001.zip › ijms-2902088-supplementary/supplement/s1/circos_plot_11.pdf]

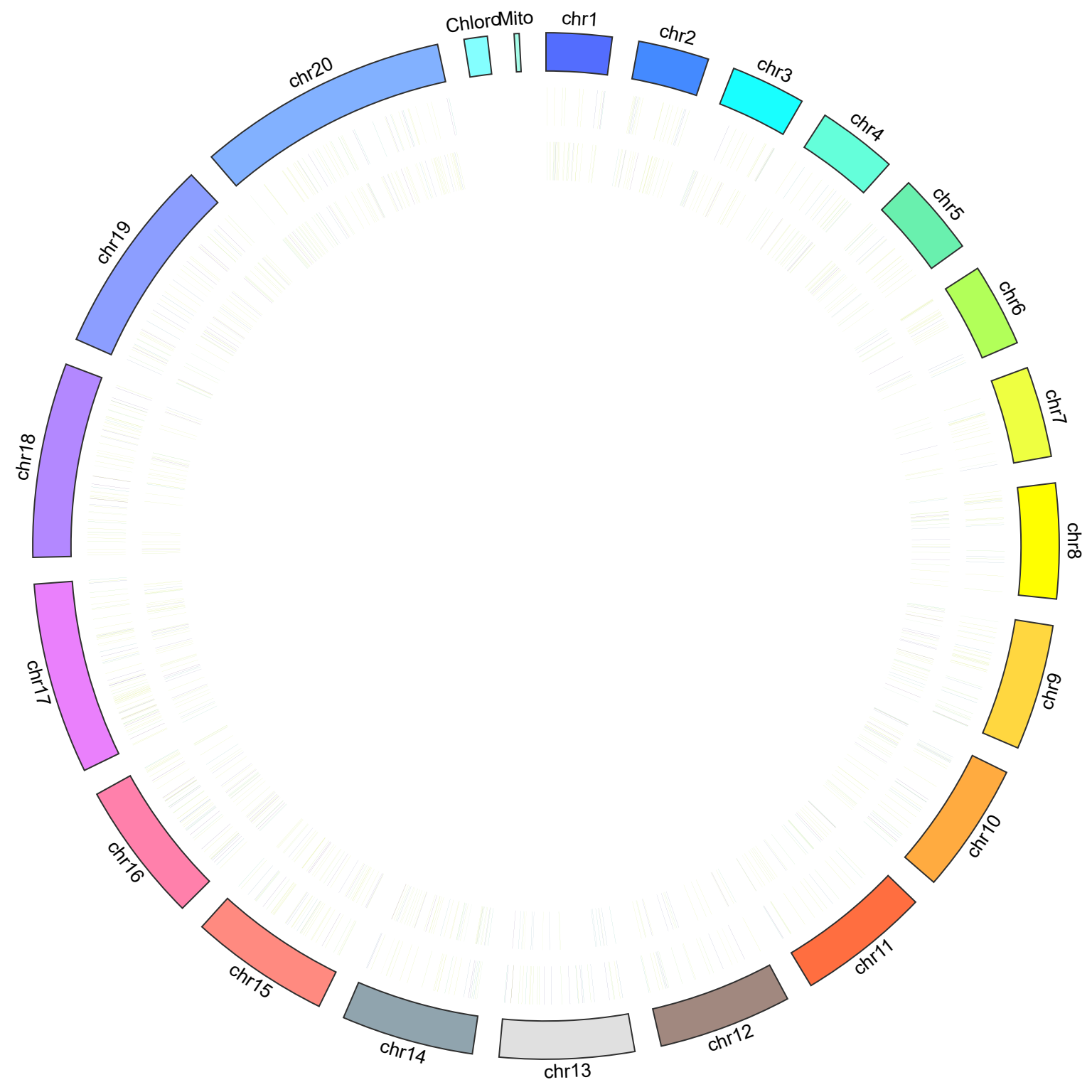

Supplement: Supplementary file 1 [file ijms-25-04441-s001.zip › ijms-2902088-supplementary/supplement/s1/circos_plot_12.pdf]

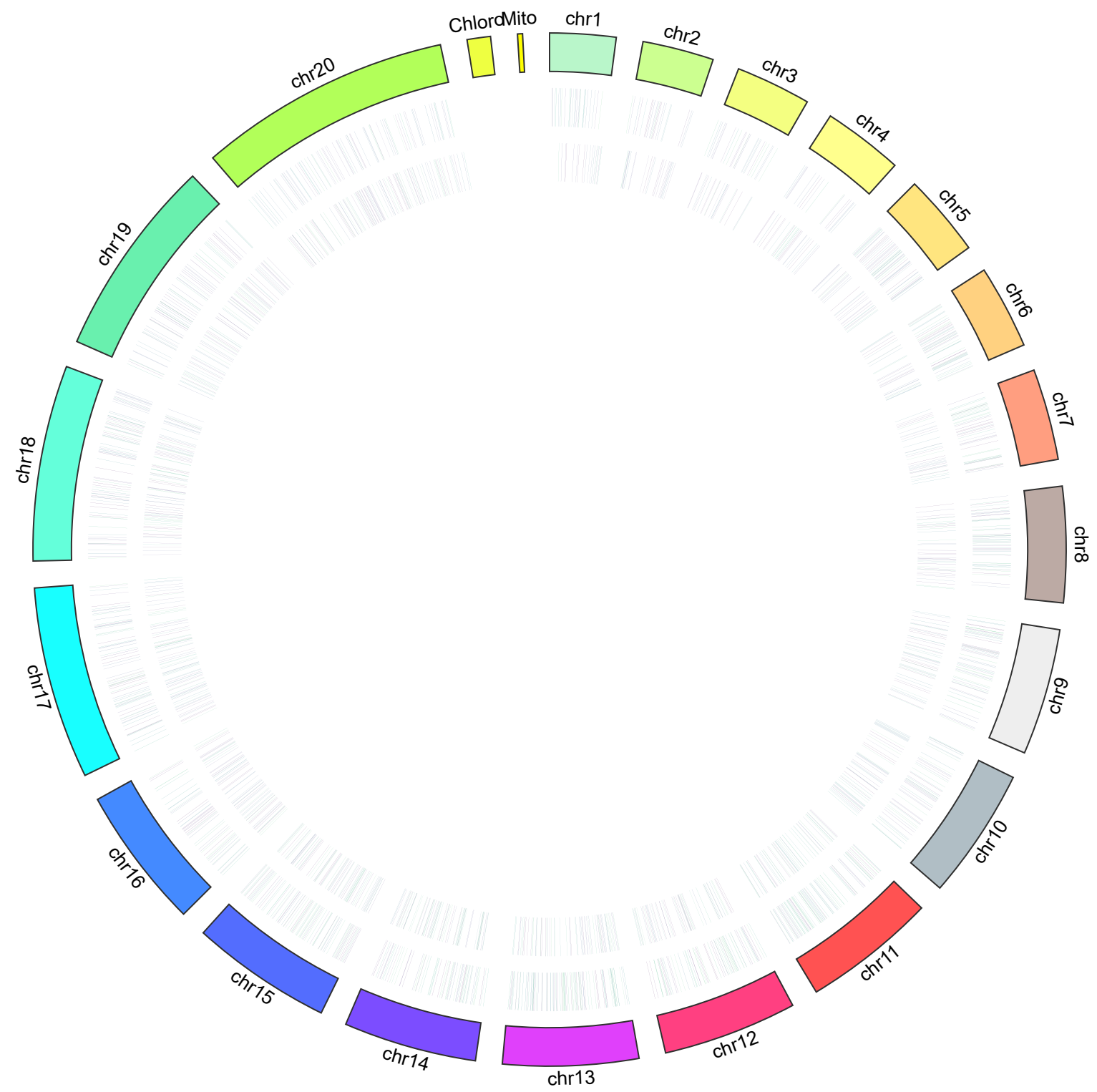

Supplement: Supplementary file 1 [file ijms-25-04441-s001.zip › ijms-2902088-supplementary/supplement/s1/circos_plot_13.pdf]

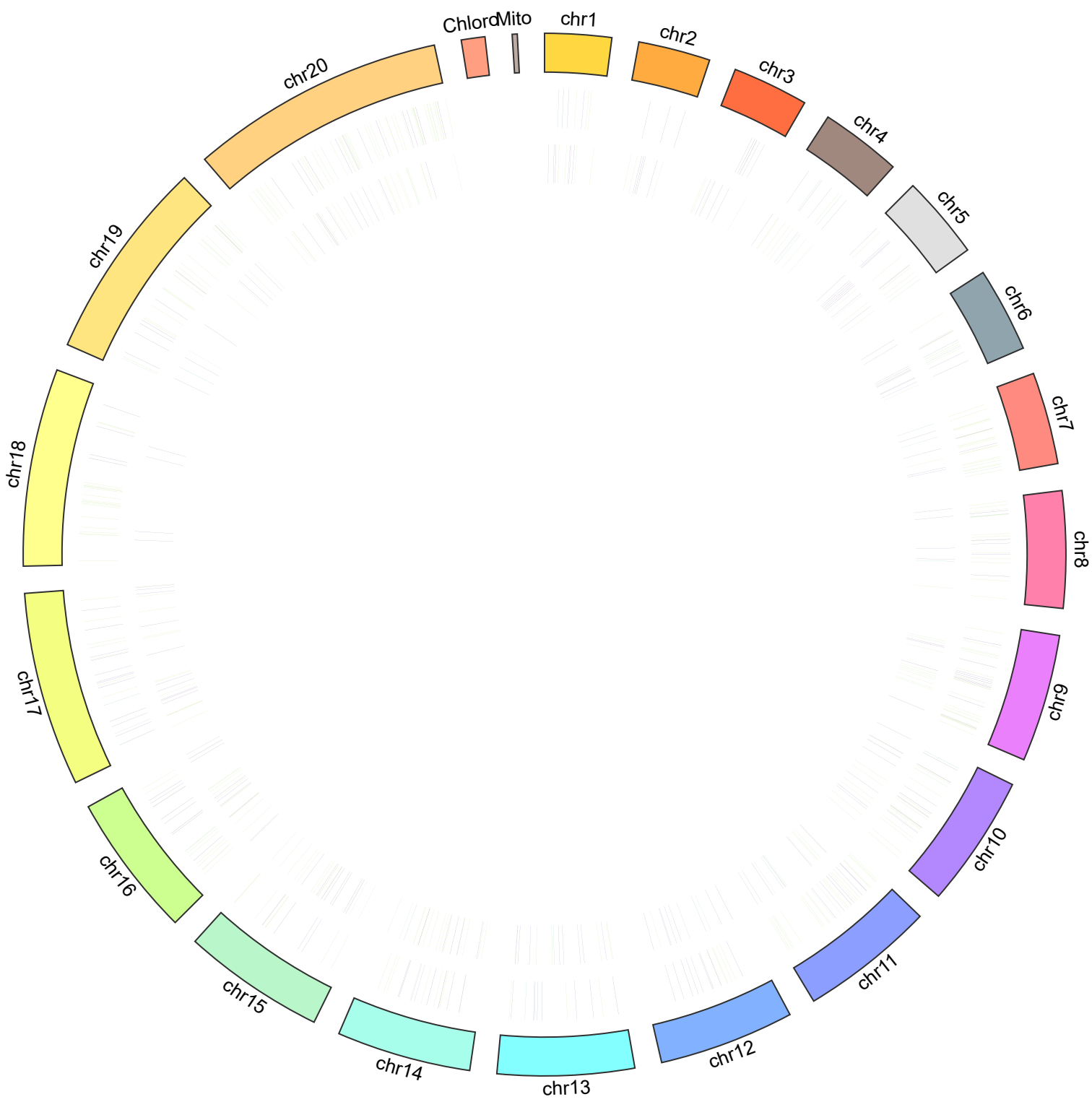

Supplement: Supplementary file 1 [file ijms-25-04441-s001.zip › ijms-2902088-supplementary/supplement/s1/circos_plot_14.pdf]

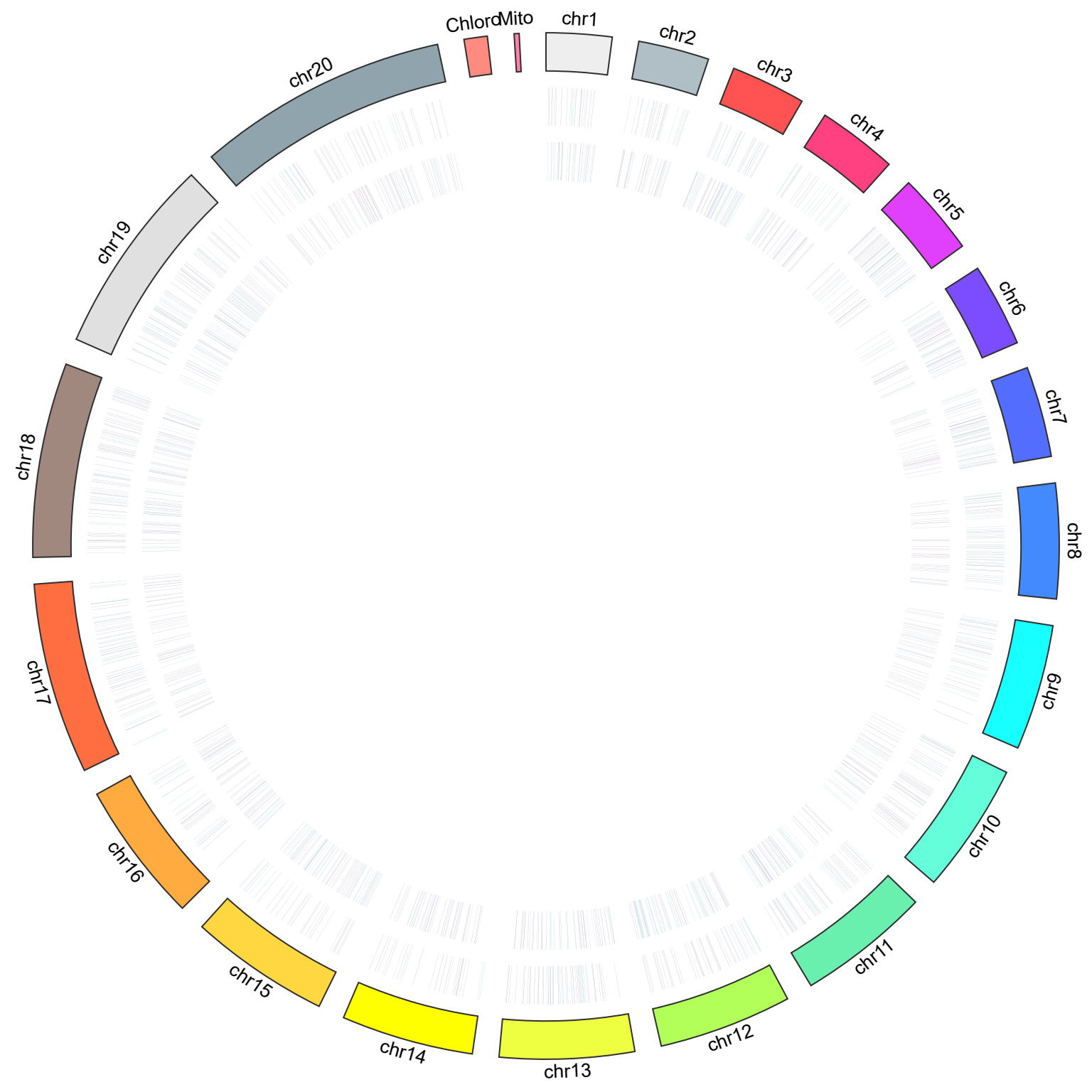

Supplement: Supplementary file 1 [file ijms-25-04441-s001.zip › ijms-2902088-supplementary/supplement/s1/circos_plot_15.pdf]

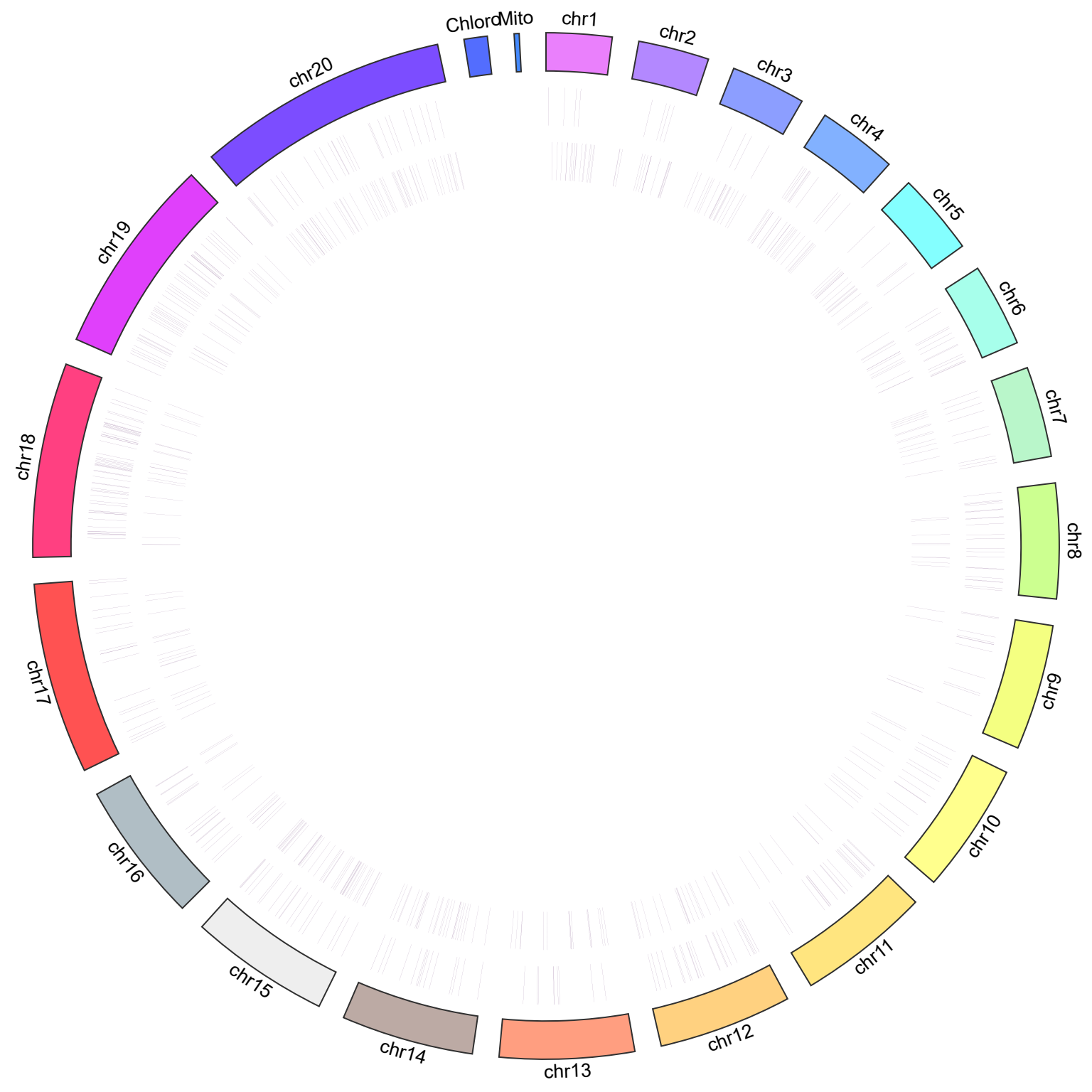

Supplement: Supplementary file 1 [file ijms-25-04441-s001.zip › ijms-2902088-supplementary/supplement/s1/circos_plot_16.pdf]

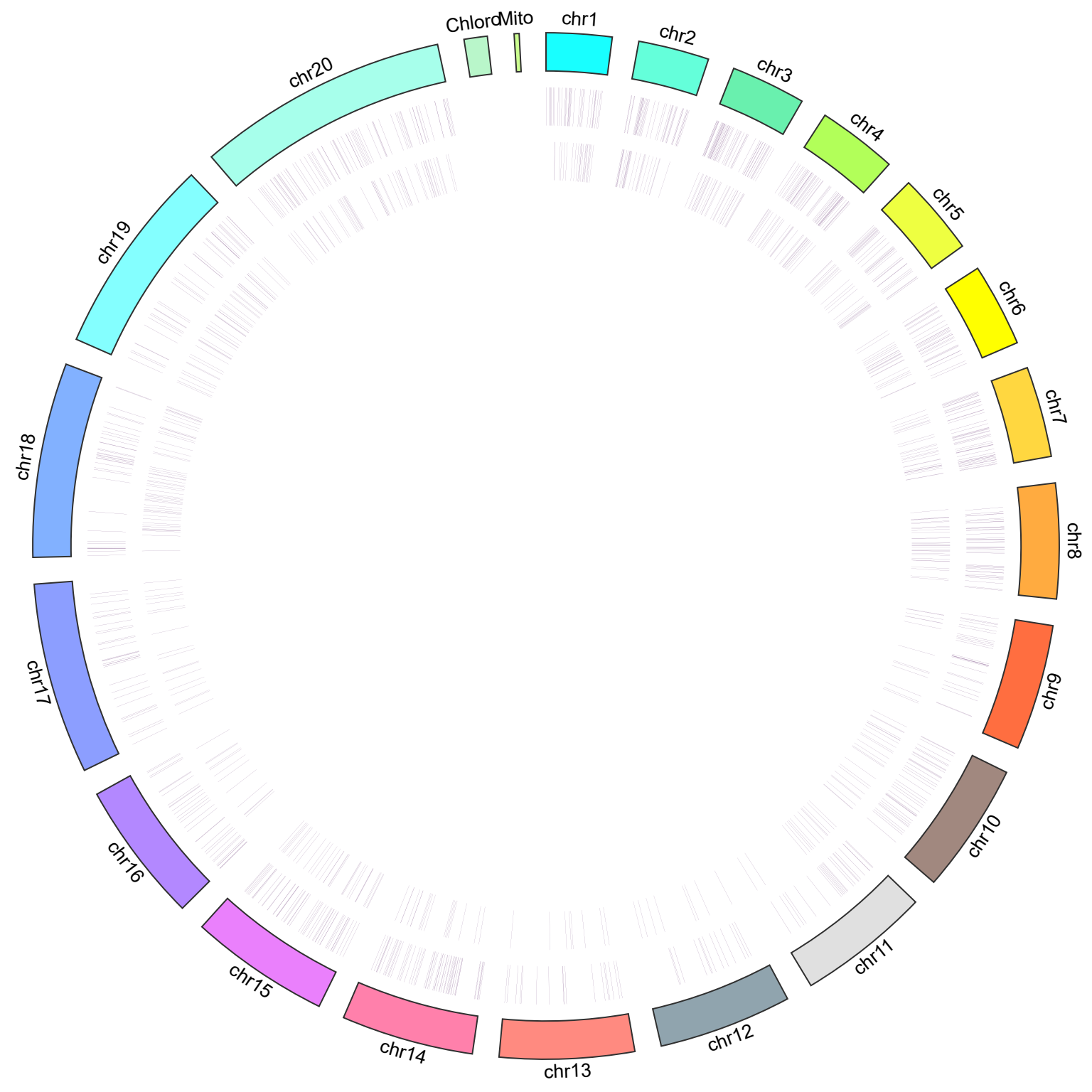

Supplement: Supplementary file 1 [file ijms-25-04441-s001.zip › ijms-2902088-supplementary/supplement/s1/circos_plot_17.pdf]

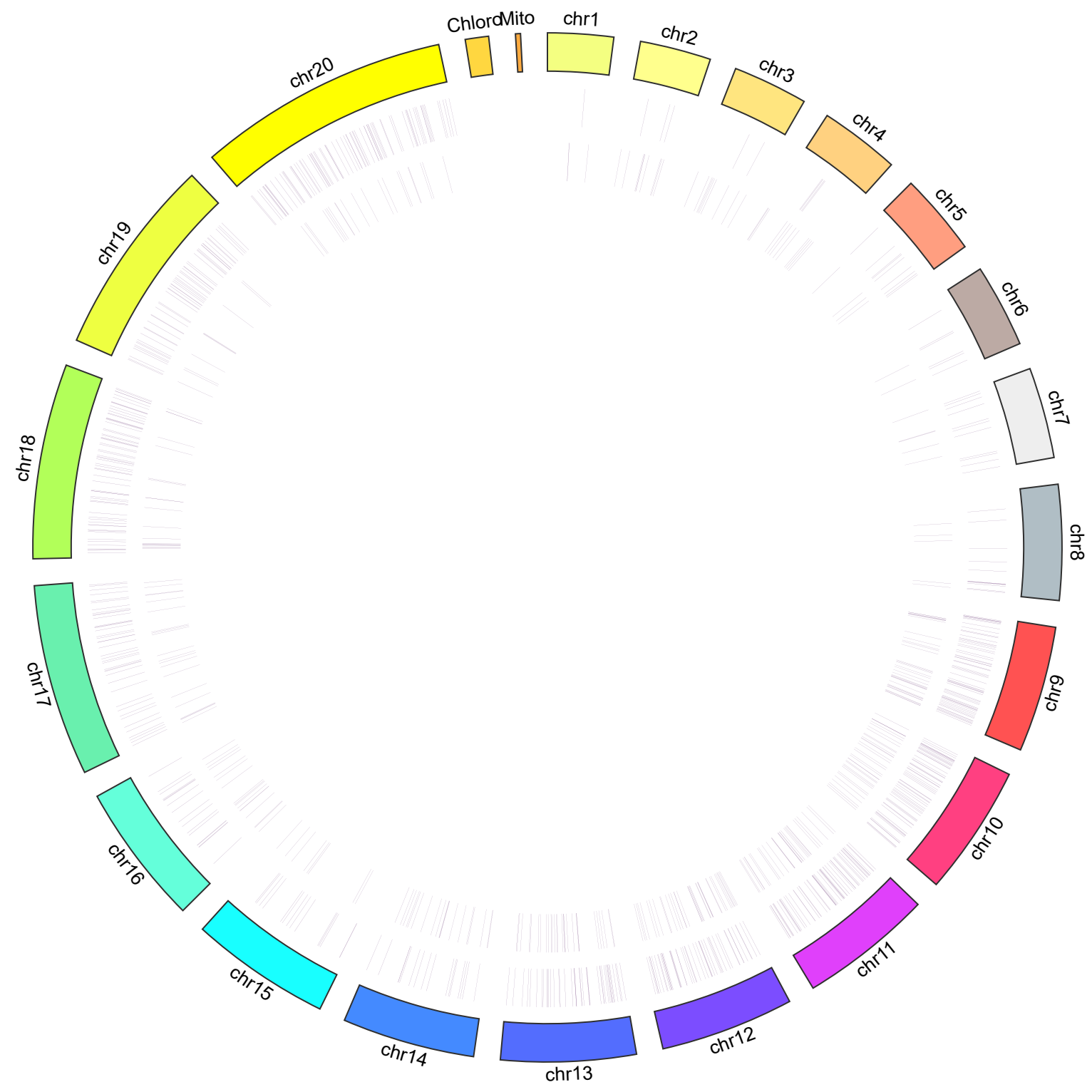

Supplement: Supplementary file 1 [file ijms-25-04441-s001.zip › ijms-2902088-supplementary/supplement/s1/circos_plot_18.pdf]

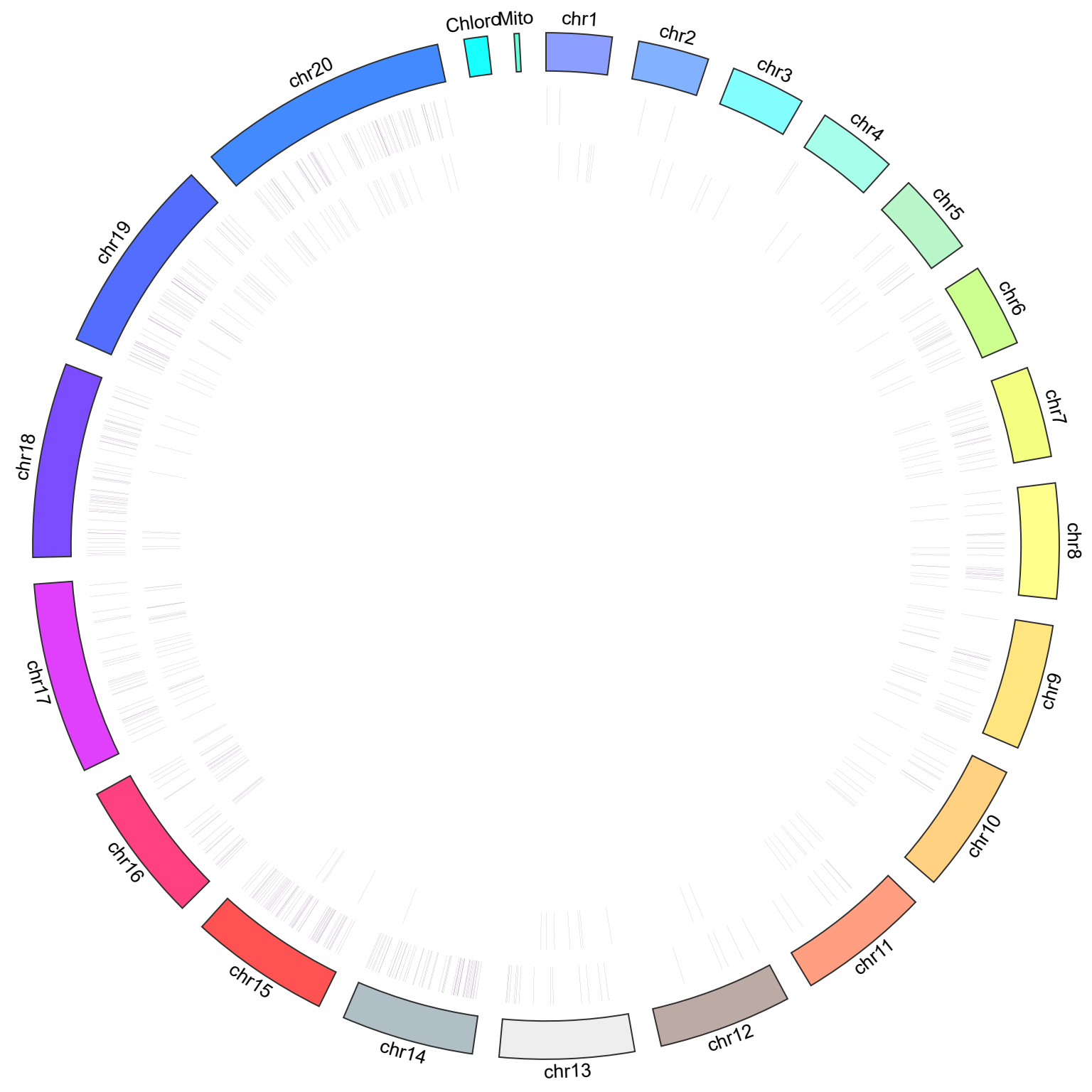

Supplement: Supplementary file 1 [file ijms-25-04441-s001.zip › ijms-2902088-supplementary/supplement/s1/circos_plot_19.pdf]

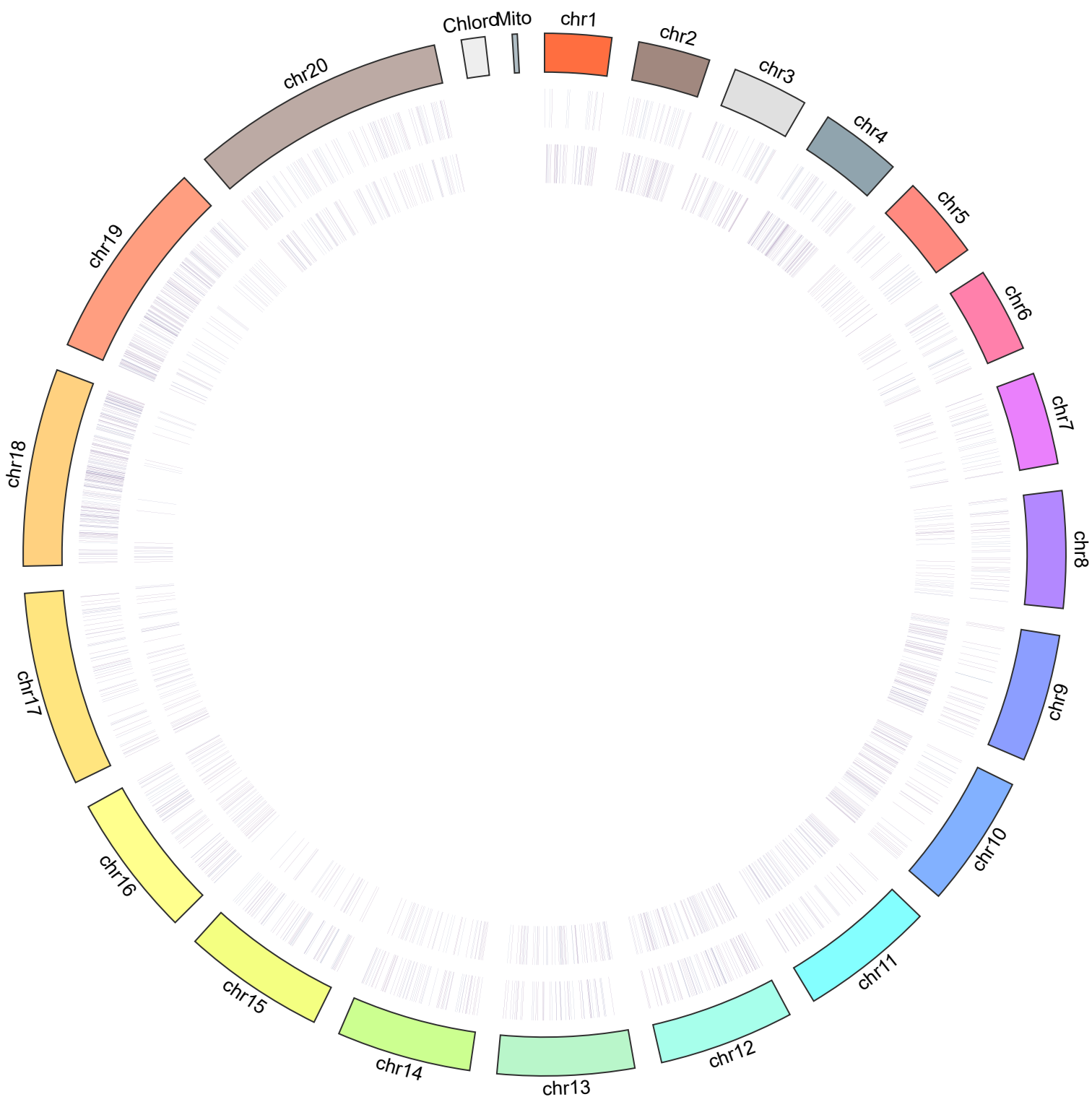

Supplement: Supplementary file 1 [file ijms-25-04441-s001.zip › ijms-2902088-supplementary/supplement/s1/circos_plot_2.pdf]

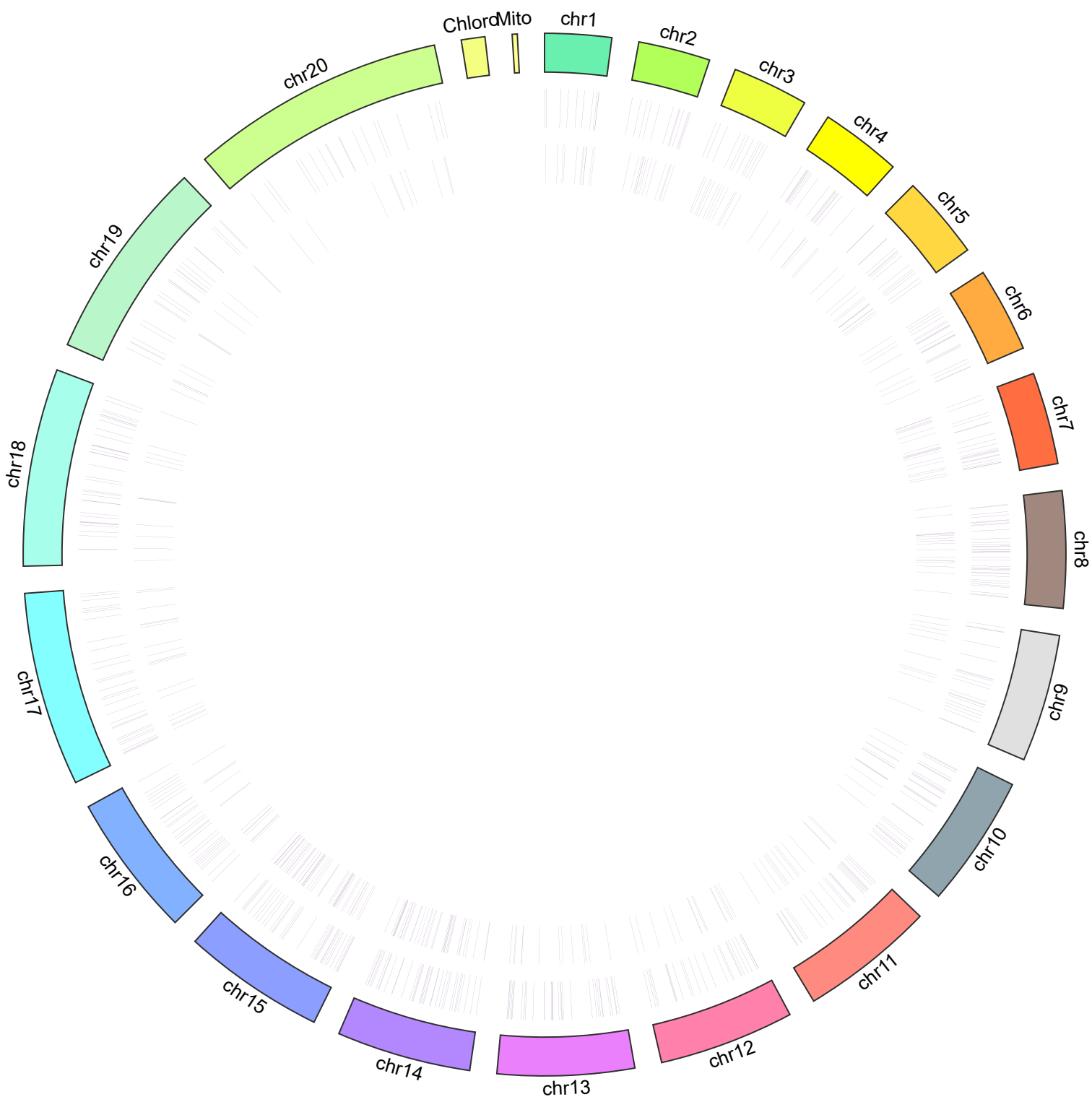

Supplement: Supplementary file 1 [file ijms-25-04441-s001.zip › ijms-2902088-supplementary/supplement/s1/circos_plot_20.pdf]

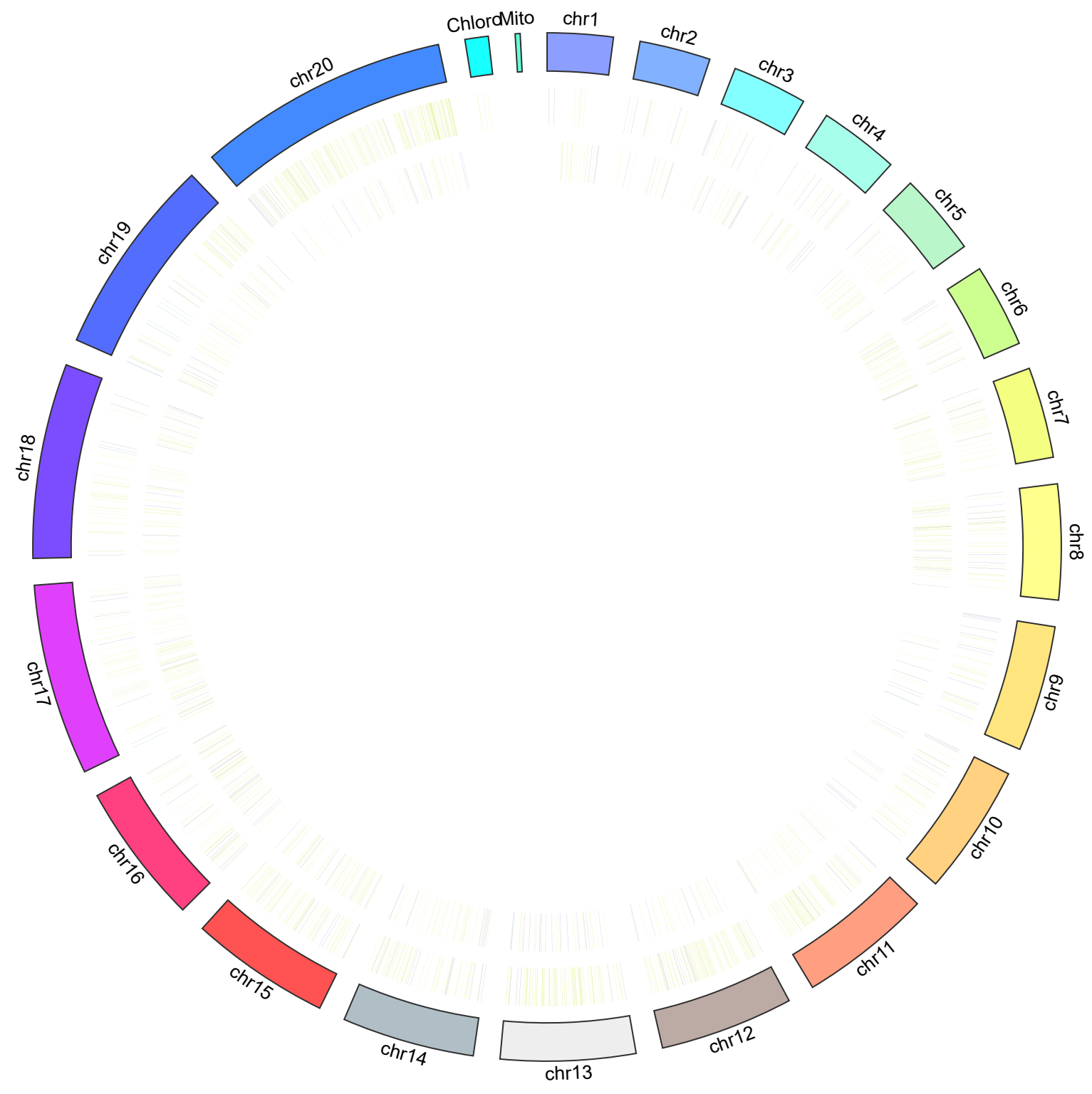

Supplement: Supplementary file 1 [file ijms-25-04441-s001.zip › ijms-2902088-supplementary/supplement/s1/circos_plot_3.pdf]

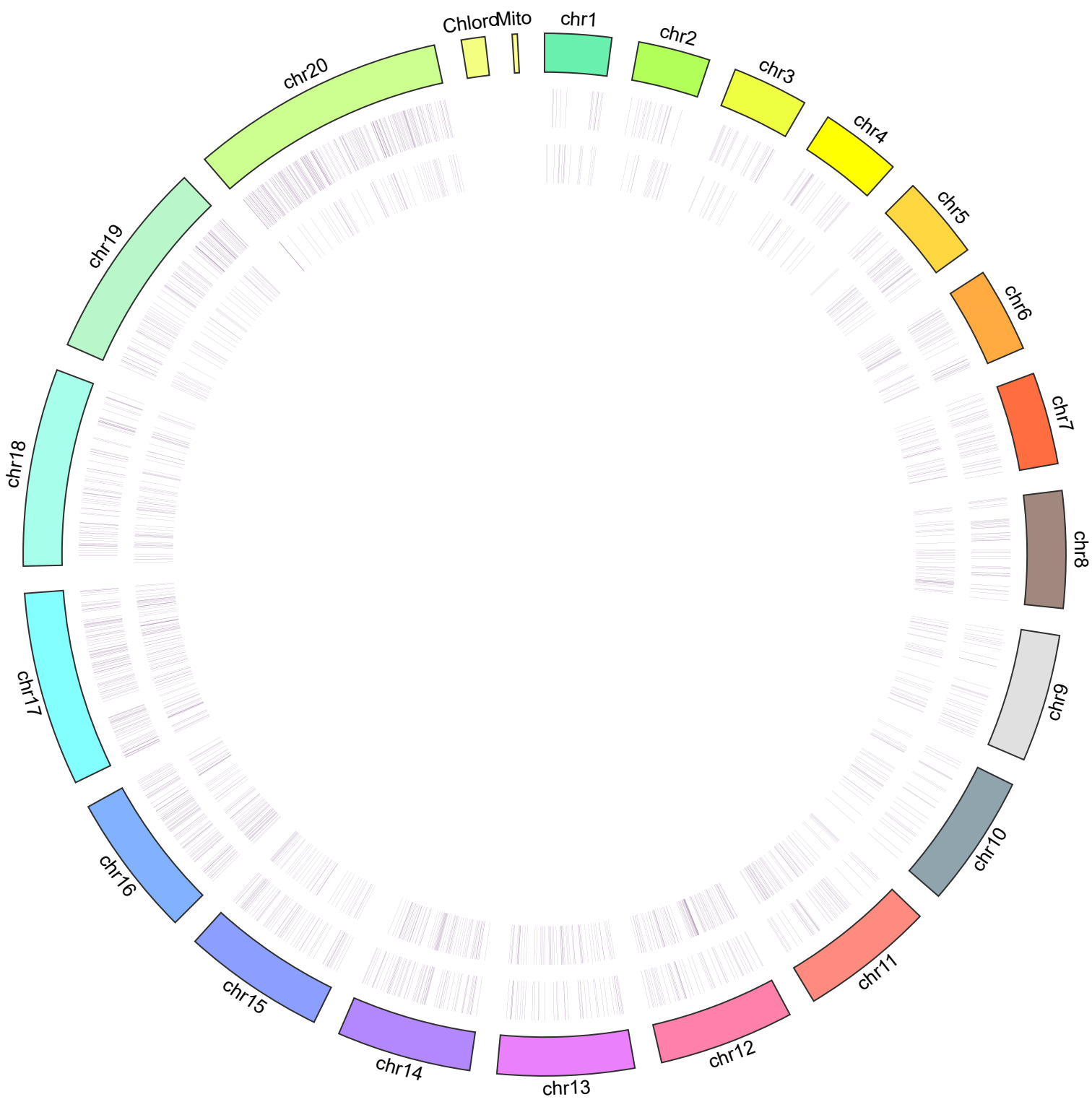

Supplement: Supplementary file 1 [file ijms-25-04441-s001.zip › ijms-2902088-supplementary/supplement/s1/circos_plot_4.pdf]

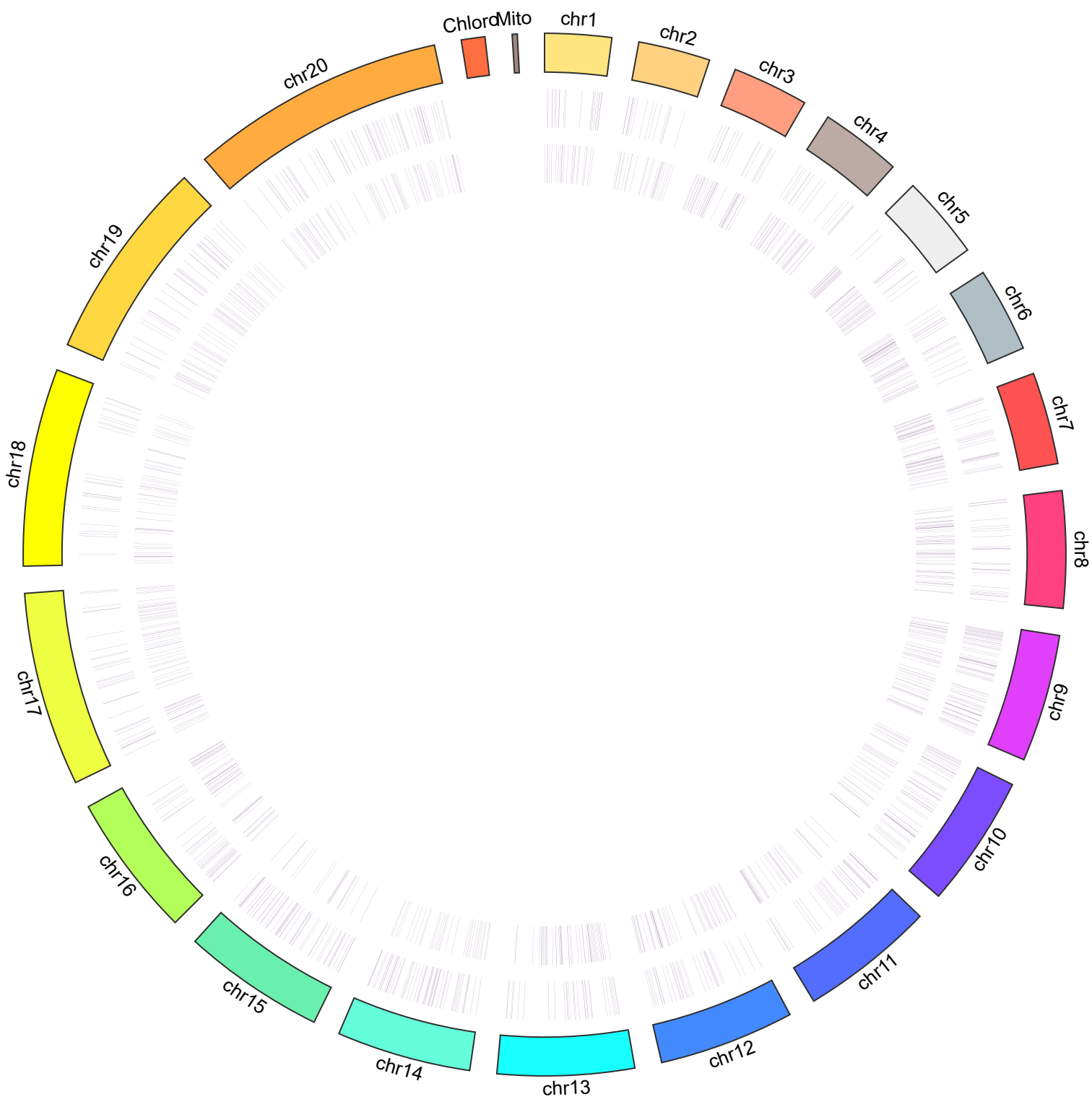

Supplement: Supplementary file 1 [file ijms-25-04441-s001.zip › ijms-2902088-supplementary/supplement/s1/circos_plot_5.pdf]

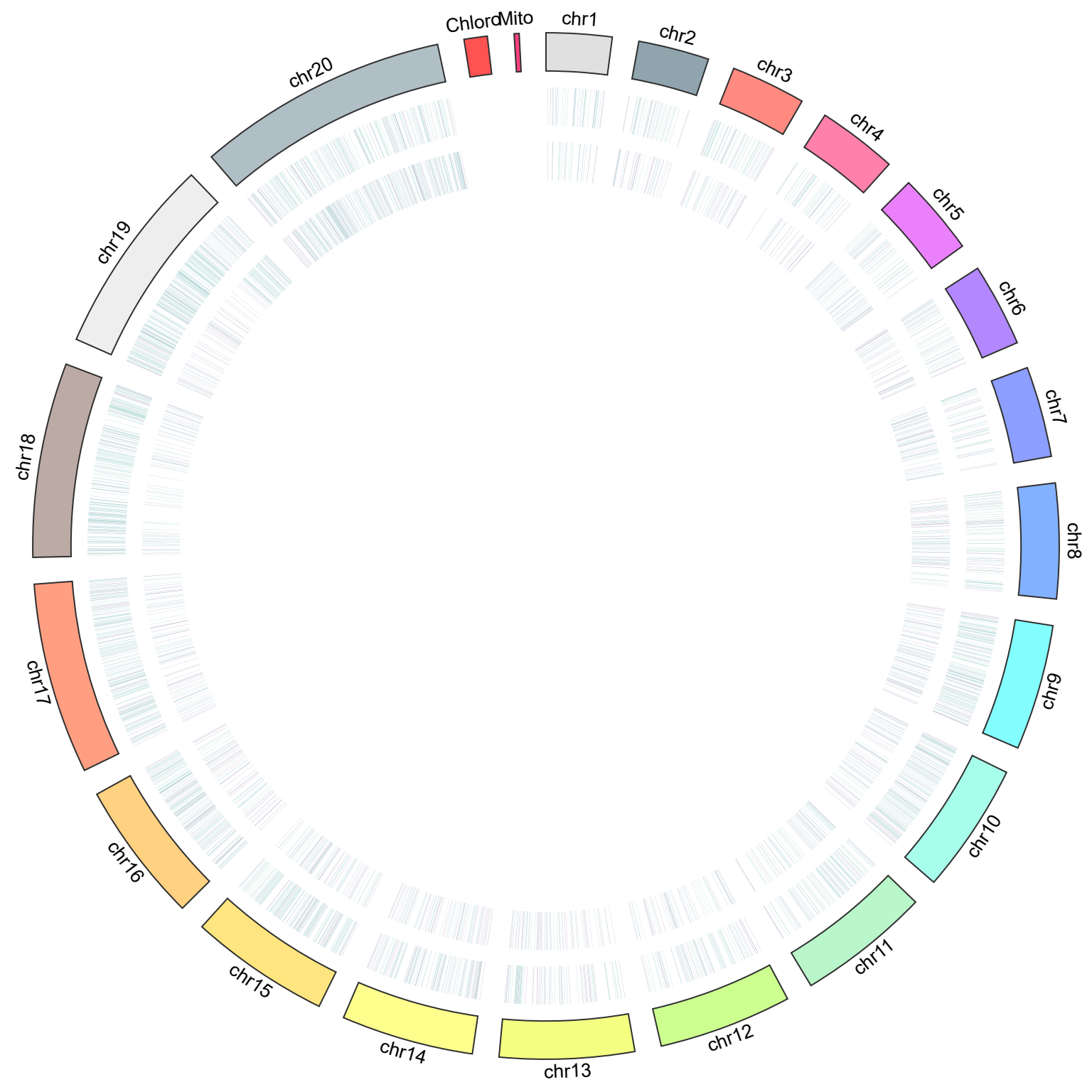

Supplement: Supplementary file 1 [file ijms-25-04441-s001.zip › ijms-2902088-supplementary/supplement/s1/circos_plot_6.pdf]

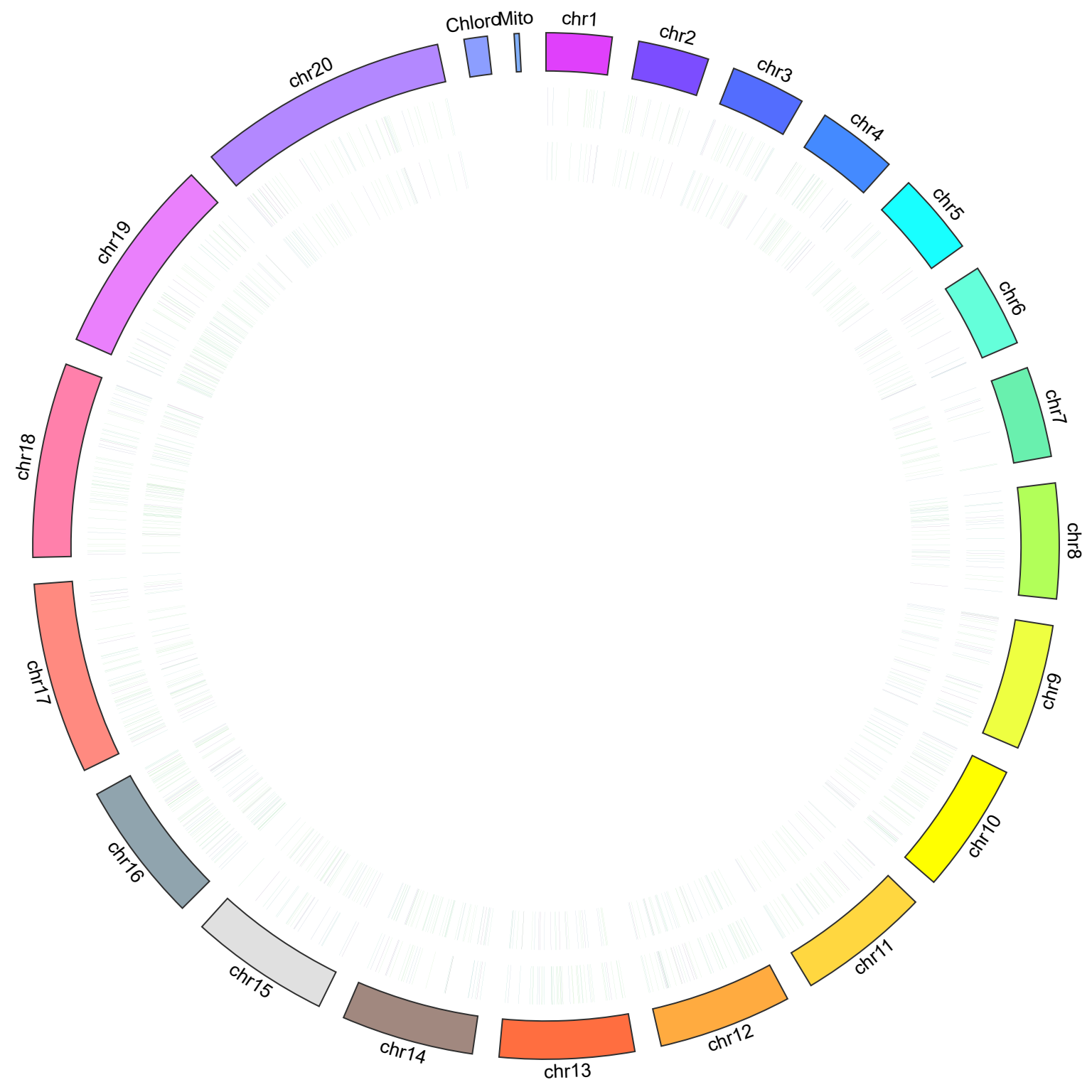

Supplement: Supplementary file 1 [file ijms-25-04441-s001.zip › ijms-2902088-supplementary/supplement/s1/circos_plot_7.pdf]

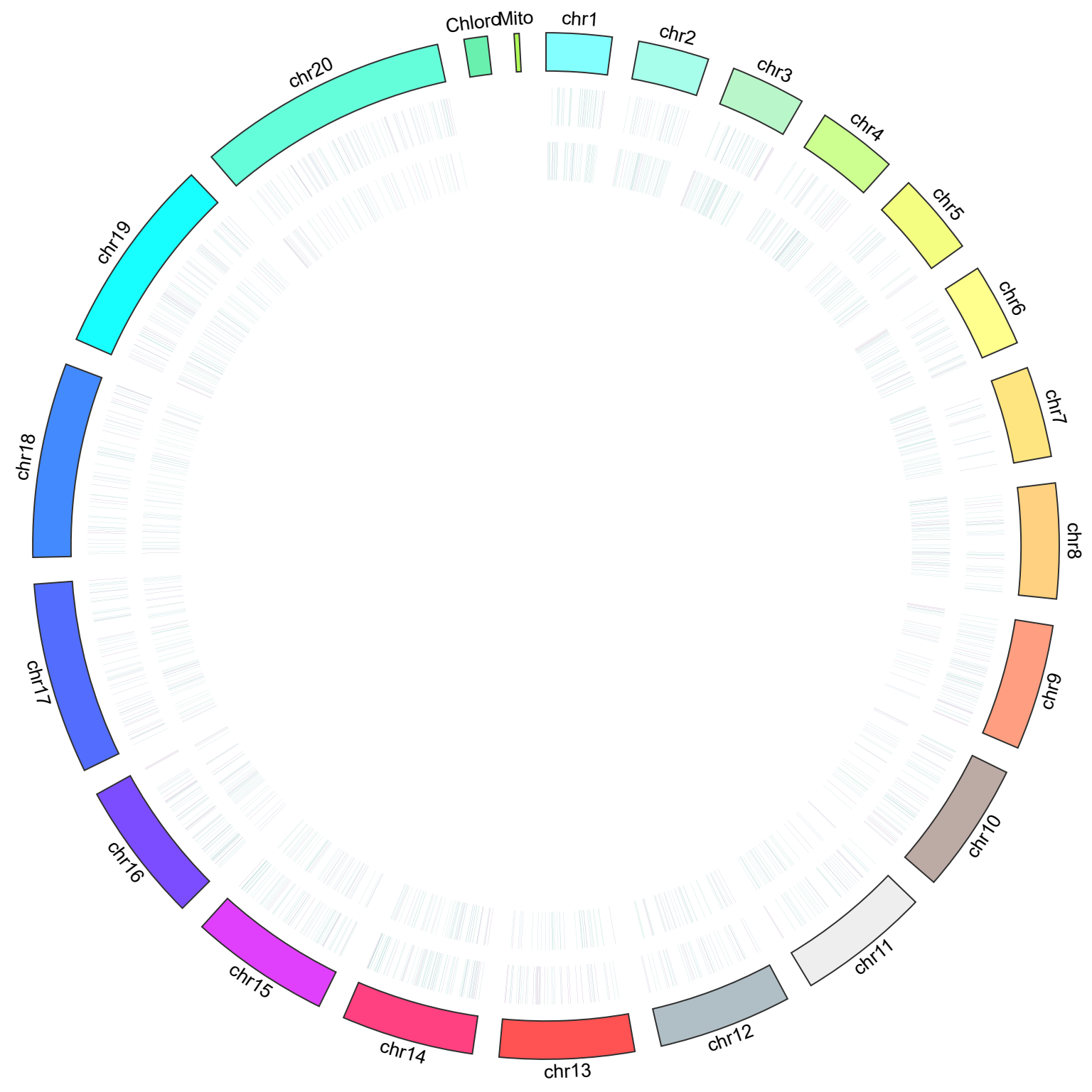

Supplement: Supplementary file 1 [file ijms-25-04441-s001.zip › ijms-2902088-supplementary/supplement/s1/circos_plot_8.pdf]

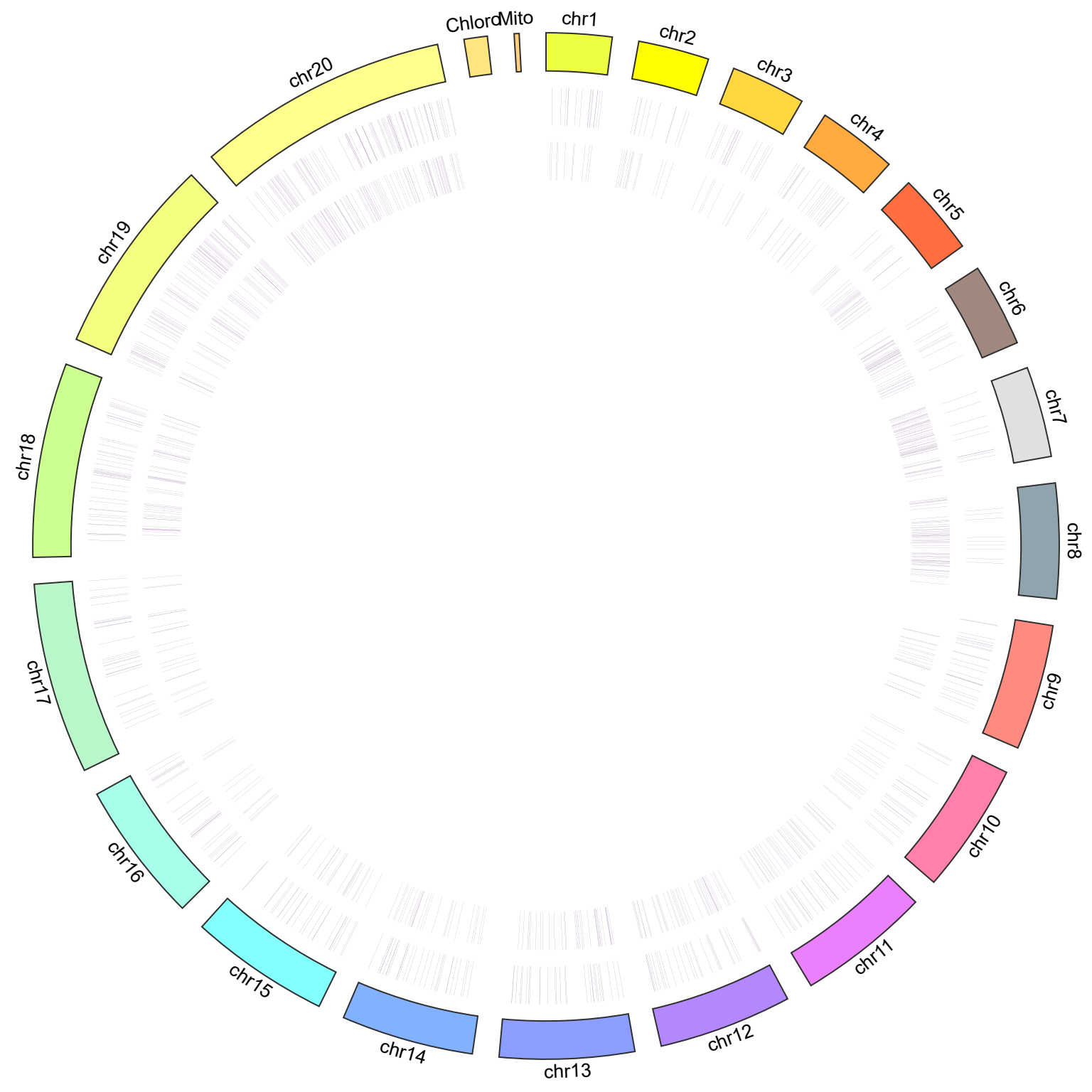

Supplement: Supplementary file 1 [file ijms-25-04441-s001.zip › ijms-2902088-supplementary/supplement/s1/circos_plot_9.pdf]

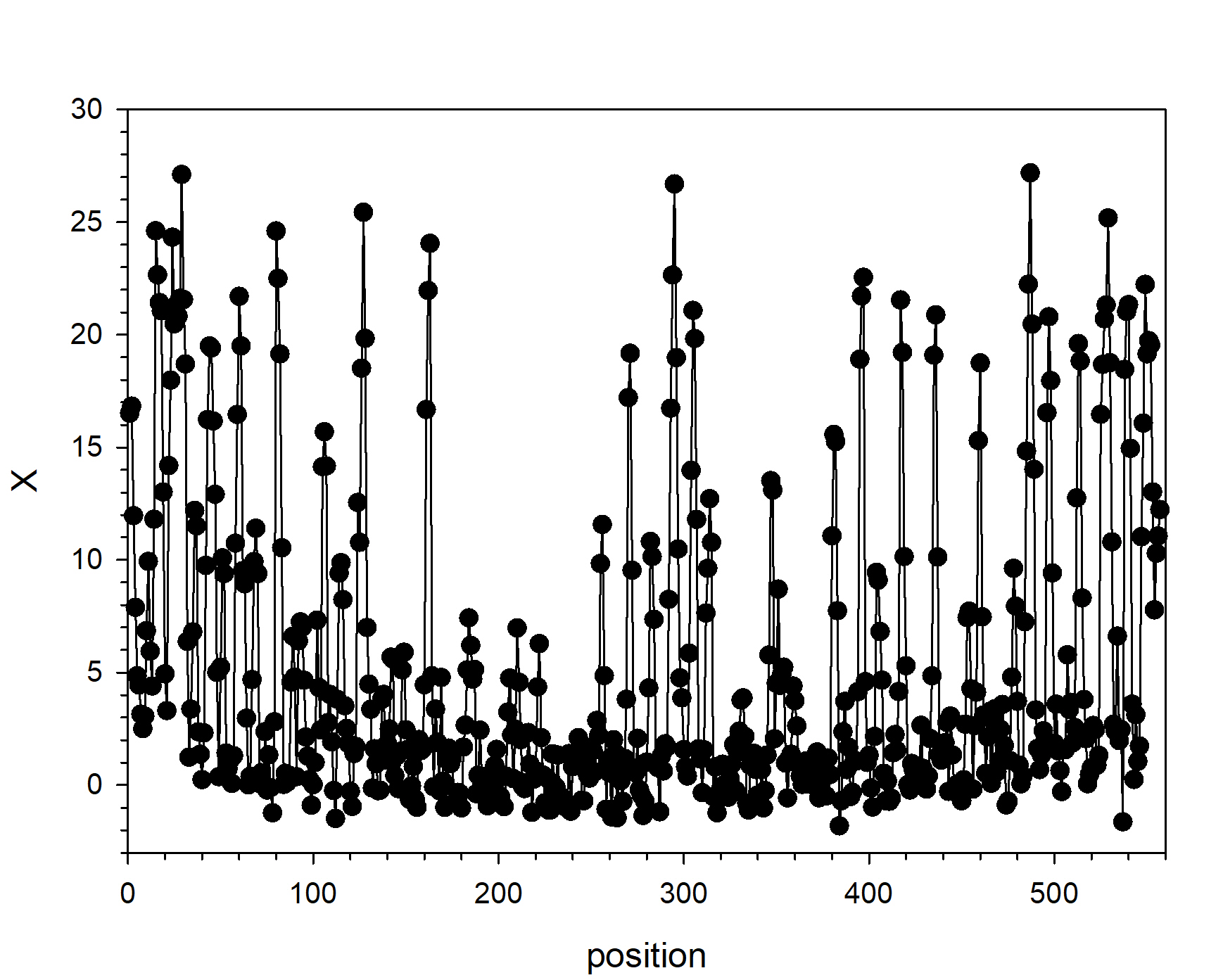

Supplement: Supplementary file 1 [file ijms-25-04441-s001.zip › ijms-2902088-supplementary/supplement/s1/fig2_1.jpg]

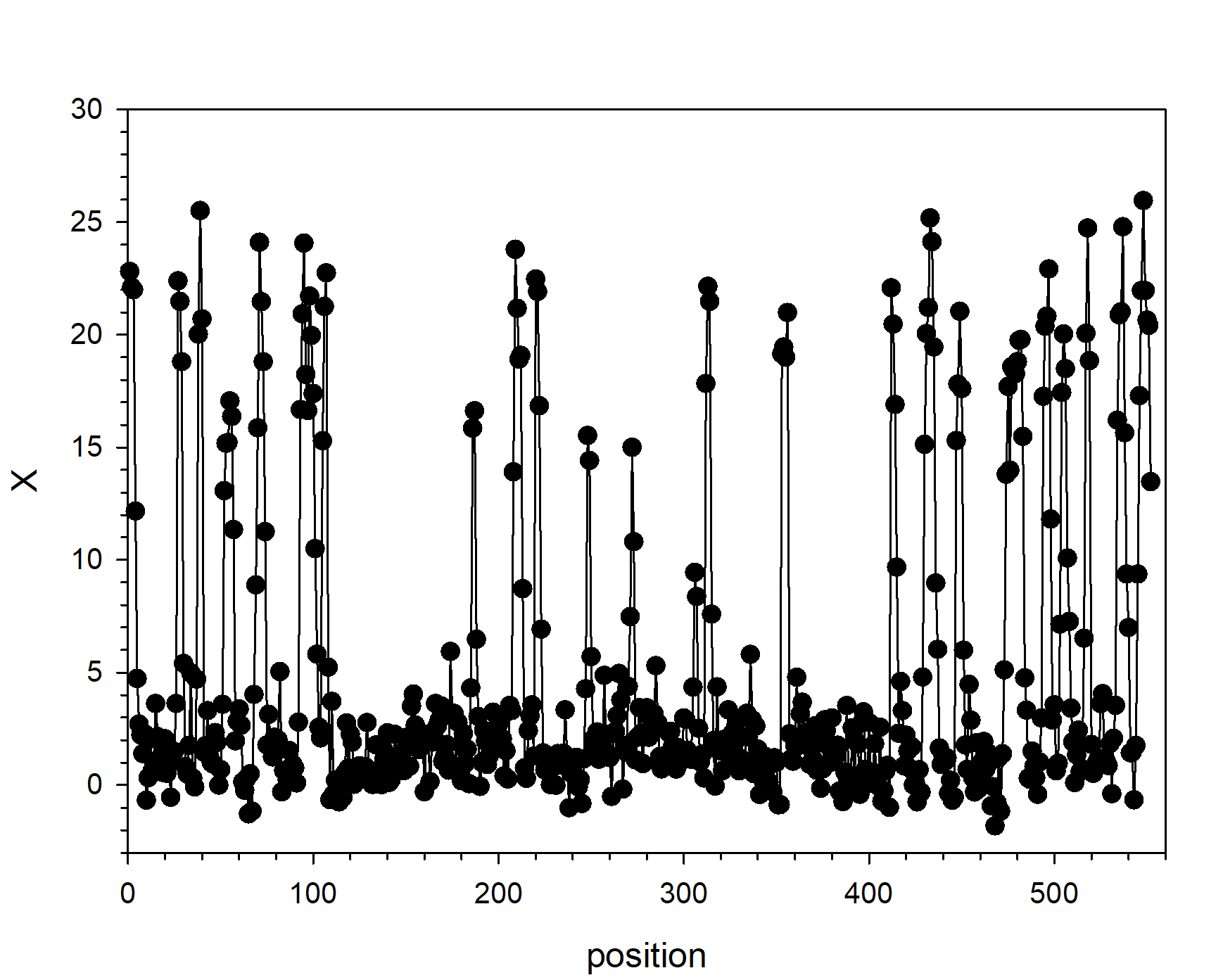

Supplement: Supplementary file 1 [file ijms-25-04441-s001.zip › ijms-2902088-supplementary/supplement/s1/fig2_10.jpg]

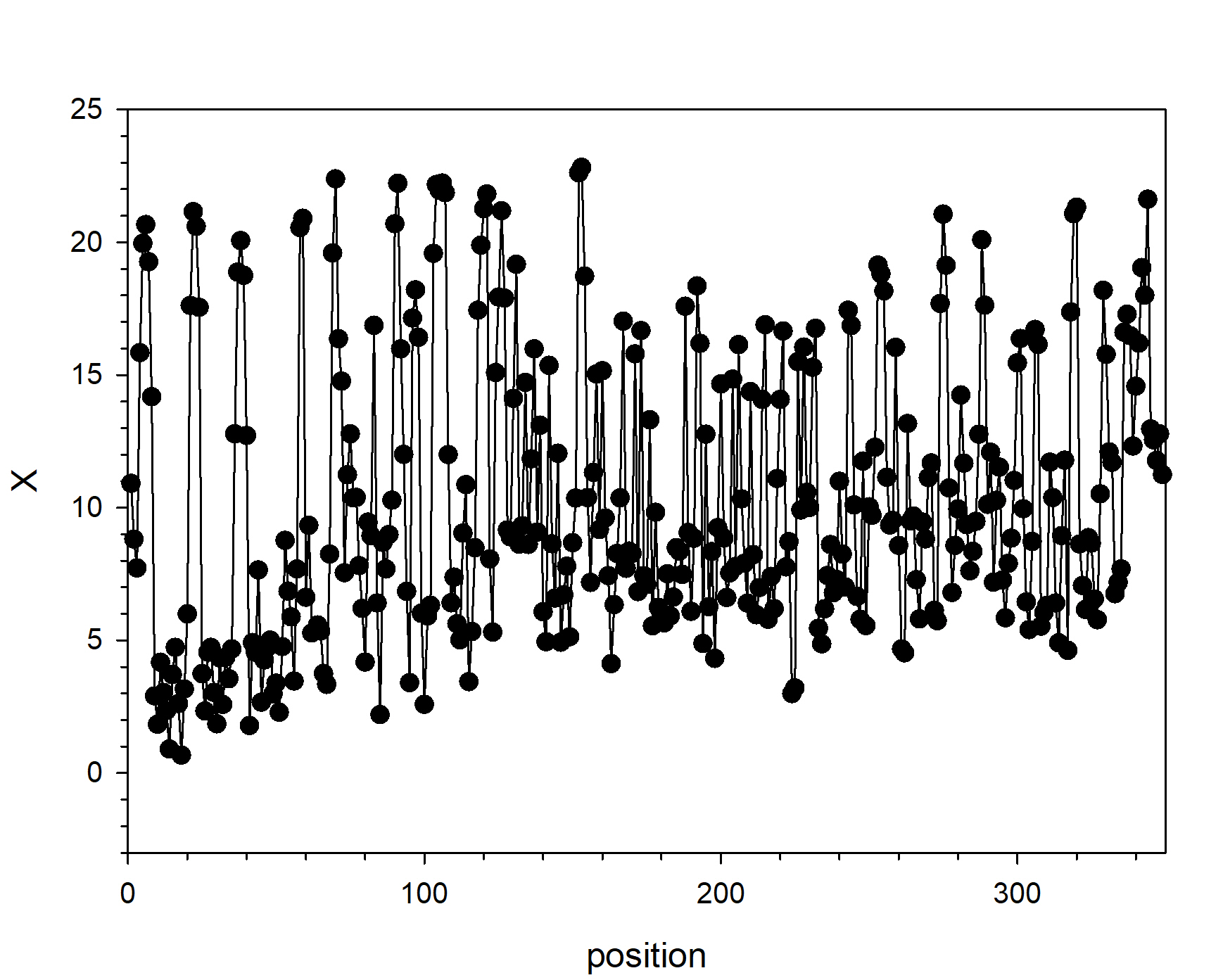

Supplement: Supplementary file 1 [file ijms-25-04441-s001.zip › ijms-2902088-supplementary/supplement/s1/fig2_11.jpg]

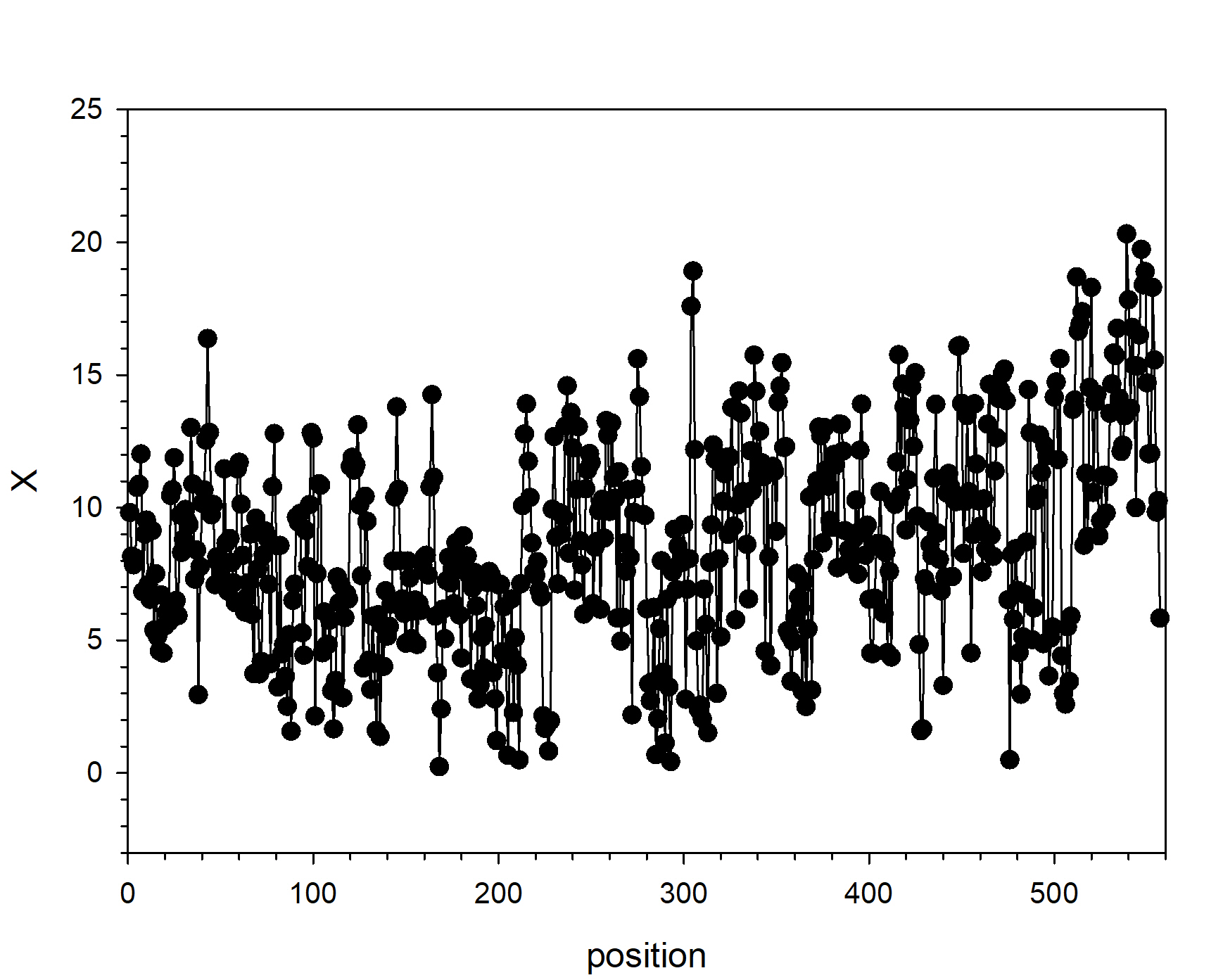

Supplement: Supplementary file 1 [file ijms-25-04441-s001.zip › ijms-2902088-supplementary/supplement/s1/fig2_12.jpg]

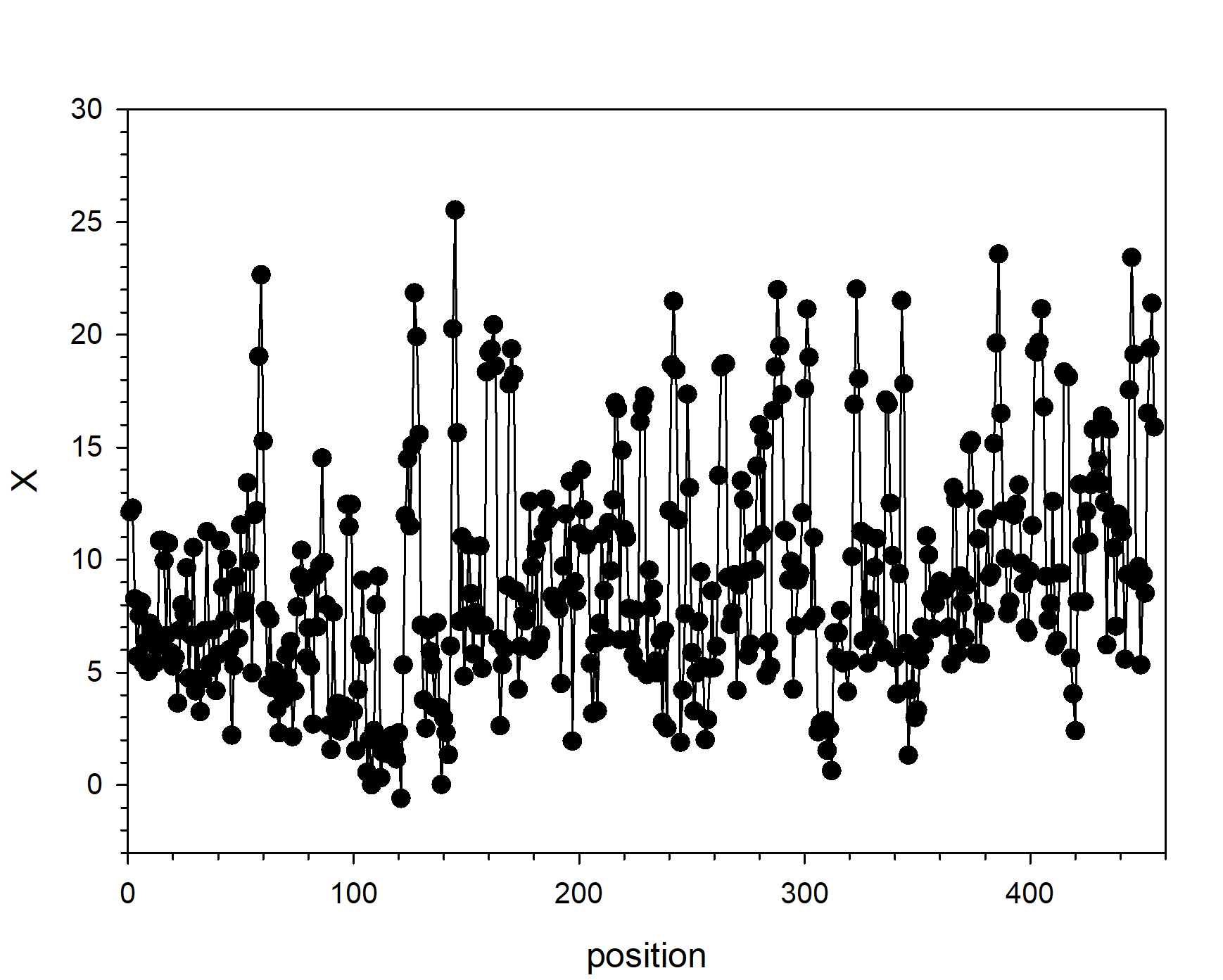

Supplement: Supplementary file 1 [file ijms-25-04441-s001.zip › ijms-2902088-supplementary/supplement/s1/fig2_13.jpg]

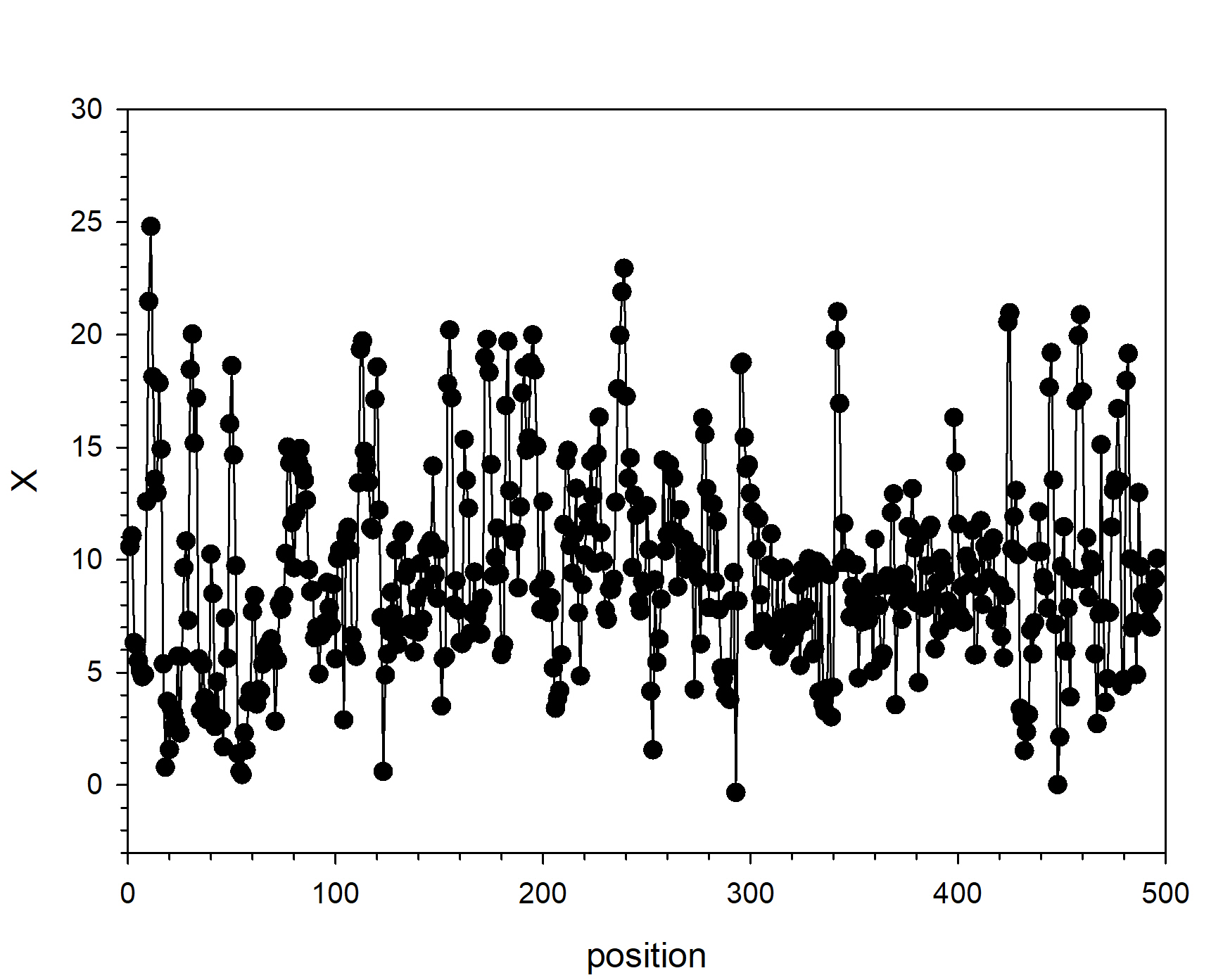

Supplement: Supplementary file 1 [file ijms-25-04441-s001.zip › ijms-2902088-supplementary/supplement/s1/fig2_14.jpg]

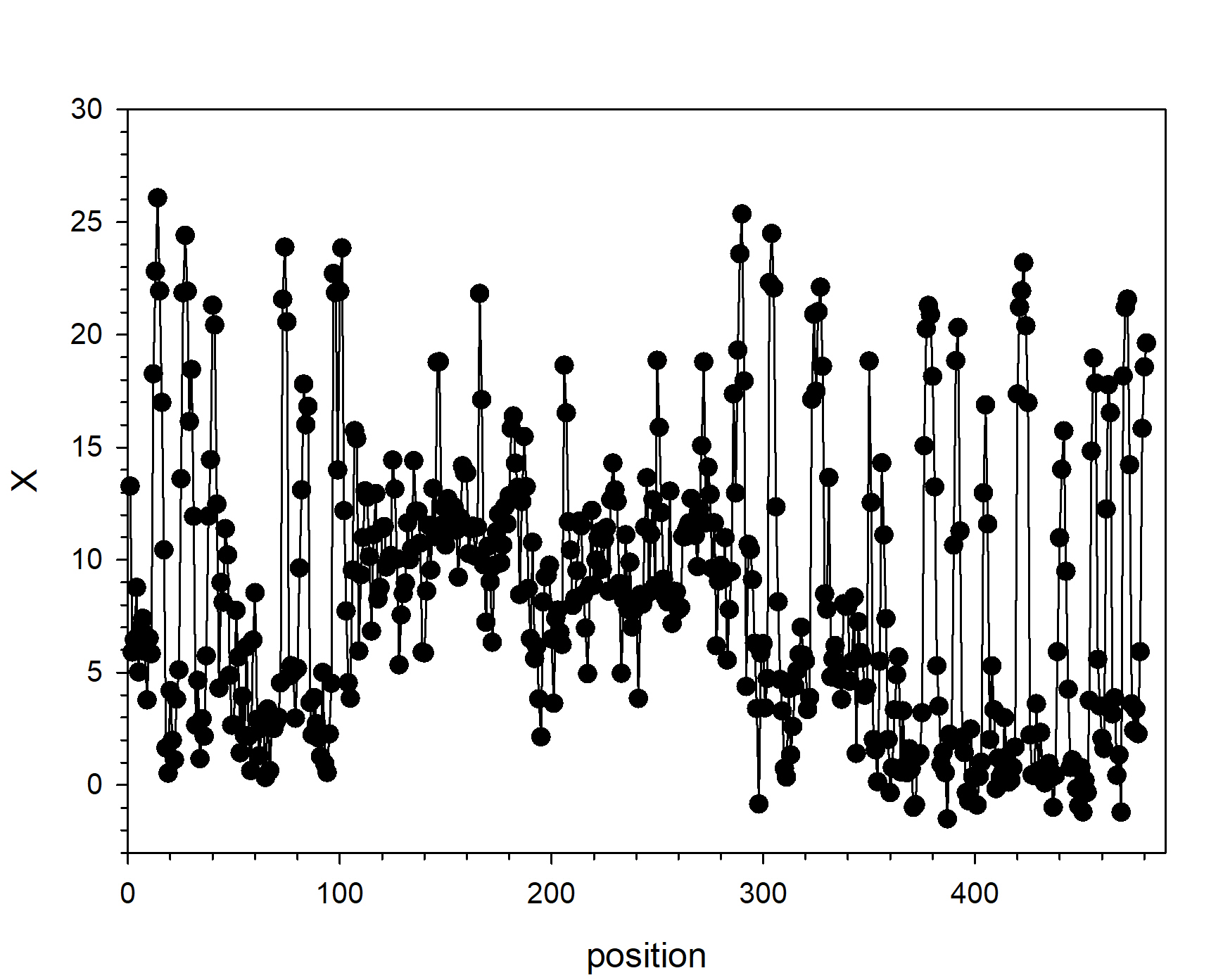

Supplement: Supplementary file 1 [file ijms-25-04441-s001.zip › ijms-2902088-supplementary/supplement/s1/fig2_15.jpg]

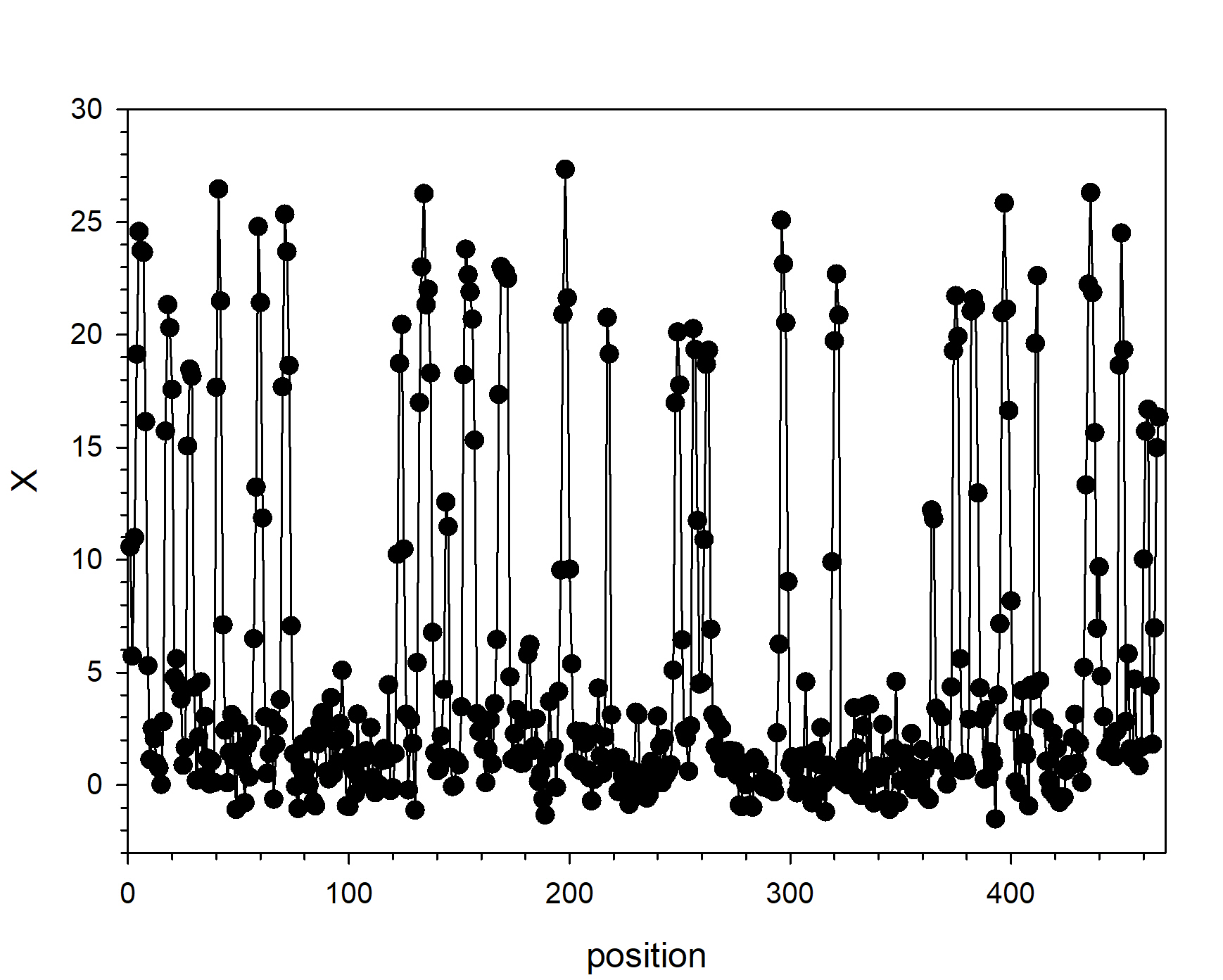

Supplement: Supplementary file 1 [file ijms-25-04441-s001.zip › ijms-2902088-supplementary/supplement/s1/fig2_16.jpg]

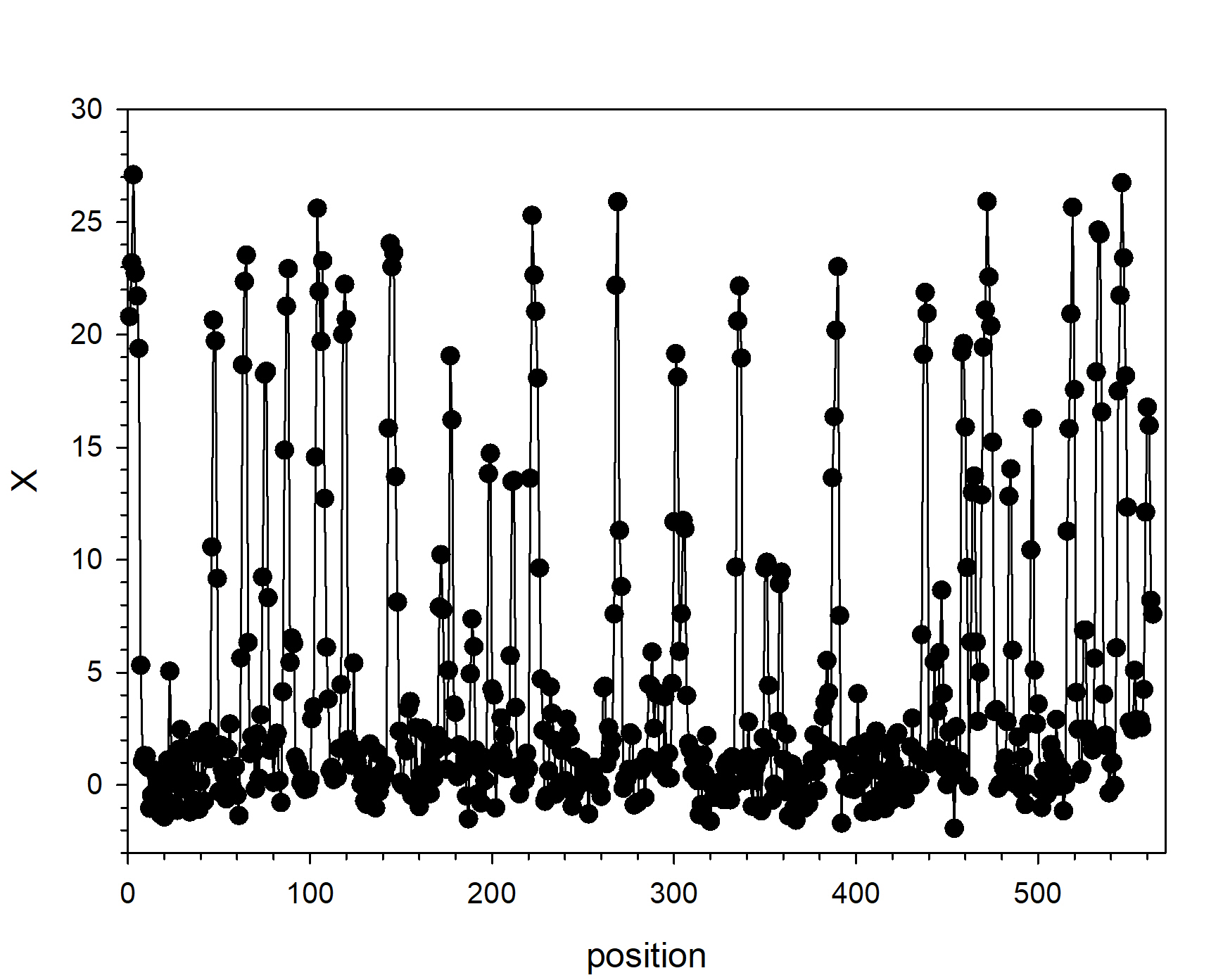

Supplement: Supplementary file 1 [file ijms-25-04441-s001.zip › ijms-2902088-supplementary/supplement/s1/fig2_17.jpg]

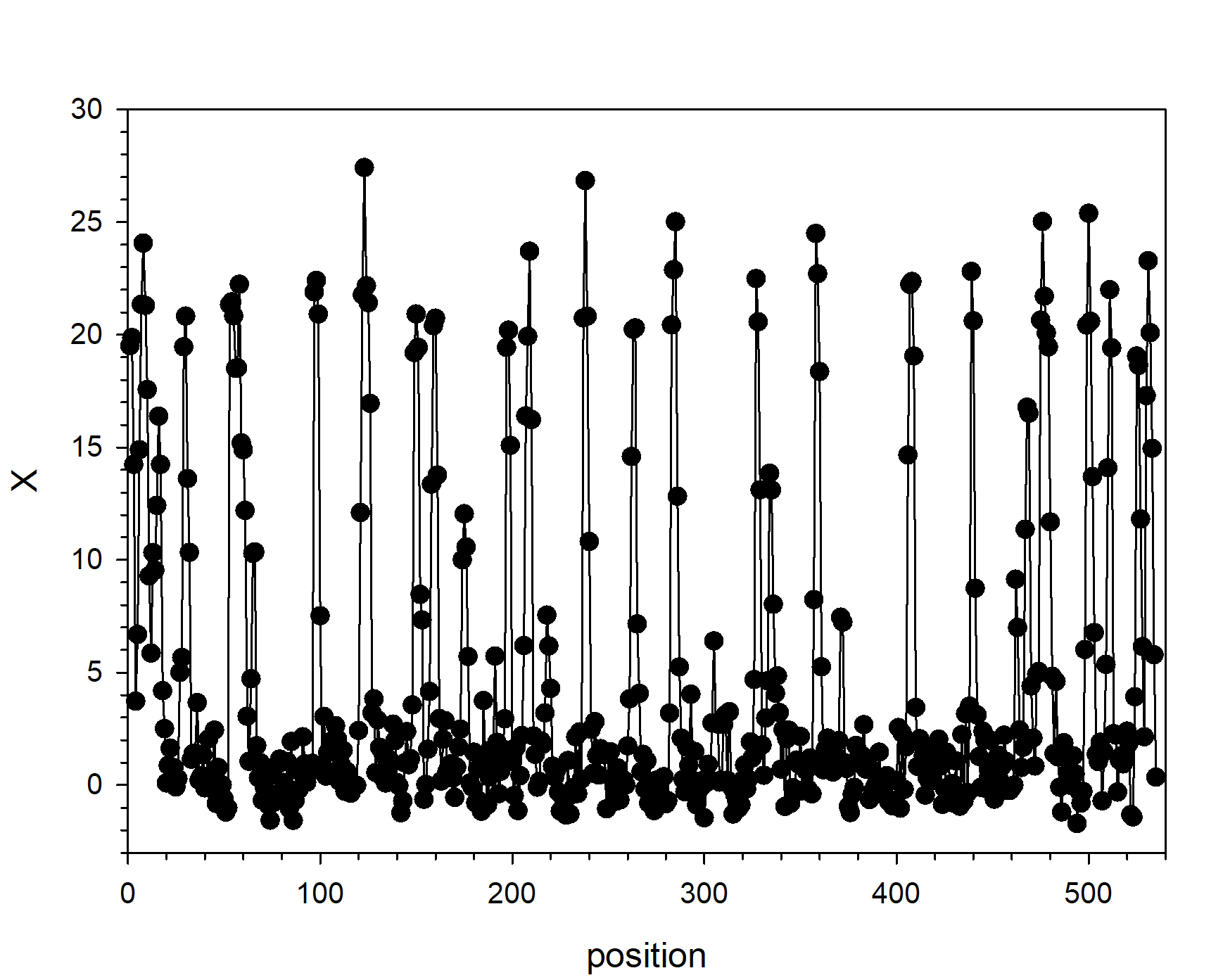

Supplement: Supplementary file 1 [file ijms-25-04441-s001.zip › ijms-2902088-supplementary/supplement/s1/fig2_18.jpg]

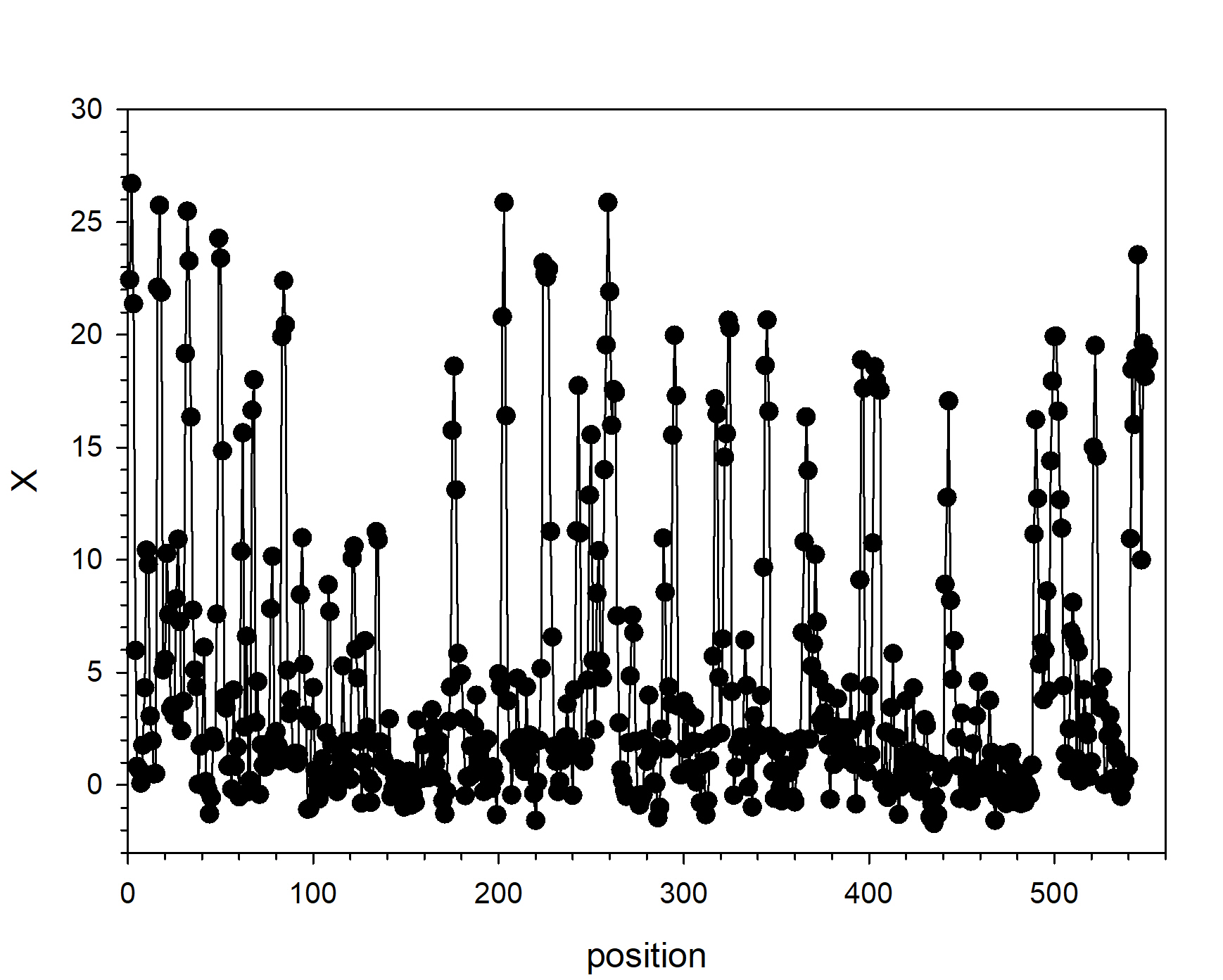

Supplement: Supplementary file 1 [file ijms-25-04441-s001.zip › ijms-2902088-supplementary/supplement/s1/fig2_19.jpg]

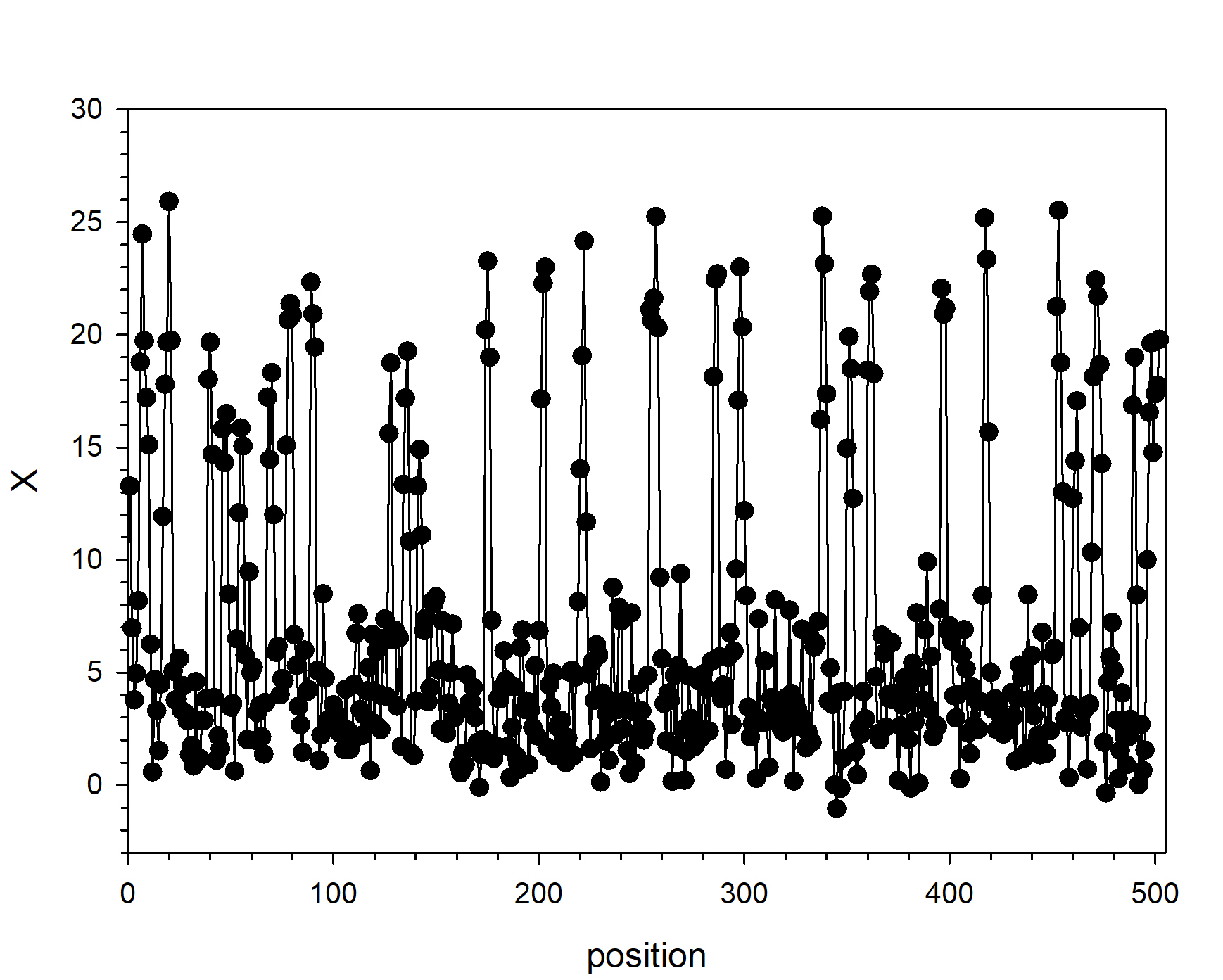

Supplement: Supplementary file 1 [file ijms-25-04441-s001.zip › ijms-2902088-supplementary/supplement/s1/fig2_2.jpg]

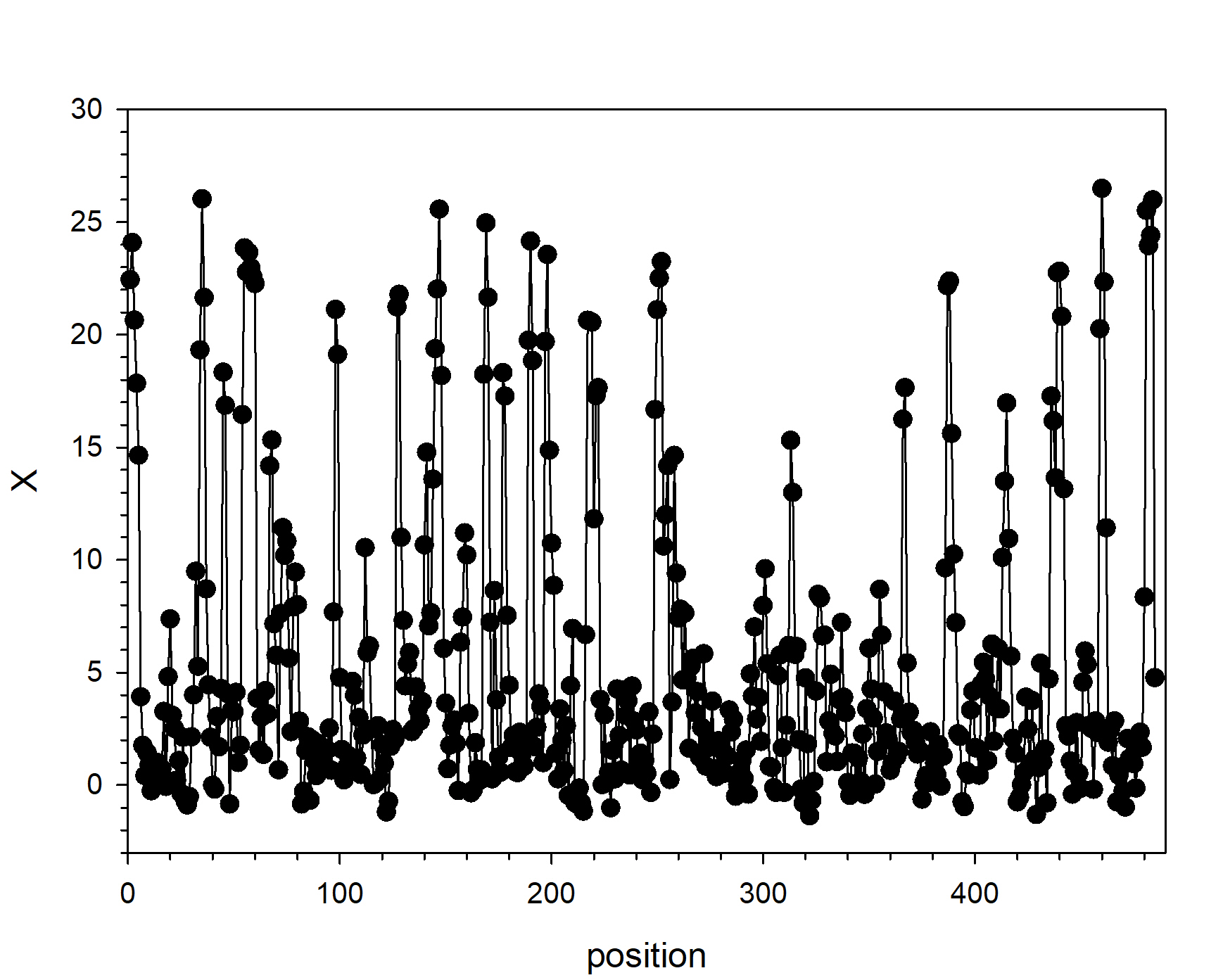

Supplement: Supplementary file 1 [file ijms-25-04441-s001.zip › ijms-2902088-supplementary/supplement/s1/fig2_20.jpg]

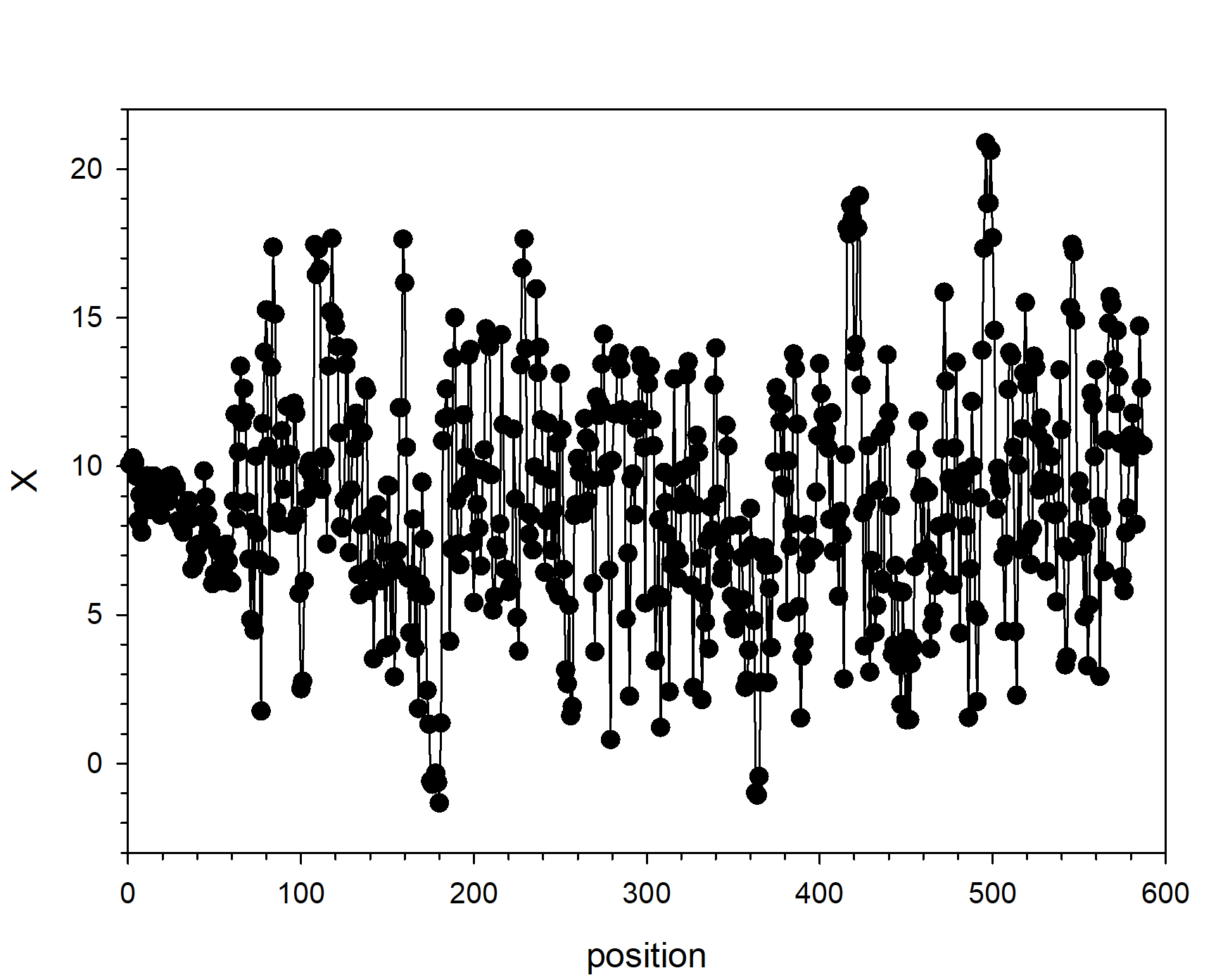

Supplement: Supplementary file 1 [file ijms-25-04441-s001.zip › ijms-2902088-supplementary/supplement/s1/fig2_3.jpg]

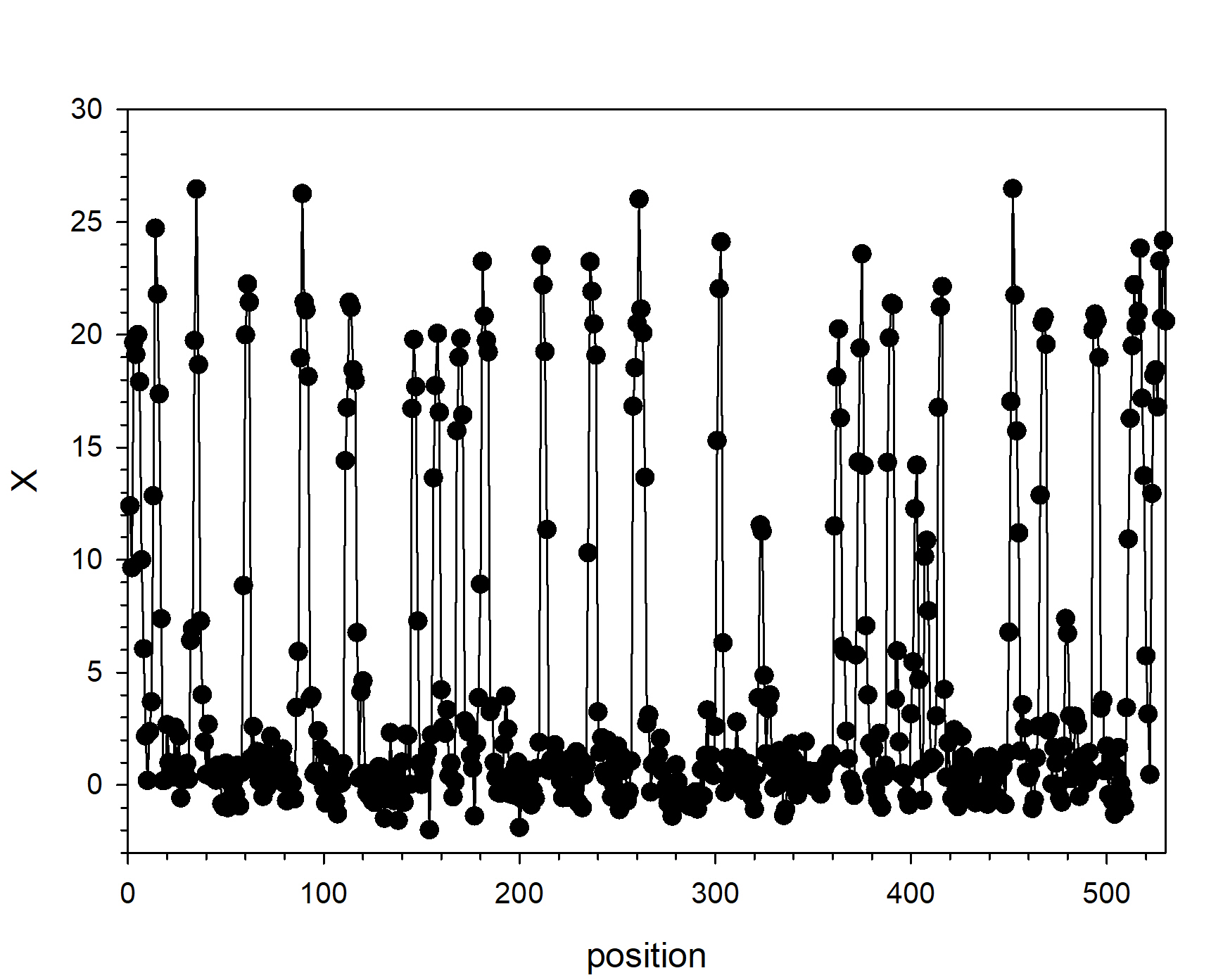

Supplement: Supplementary file 1 [file ijms-25-04441-s001.zip › ijms-2902088-supplementary/supplement/s1/fig2_4.jpg]

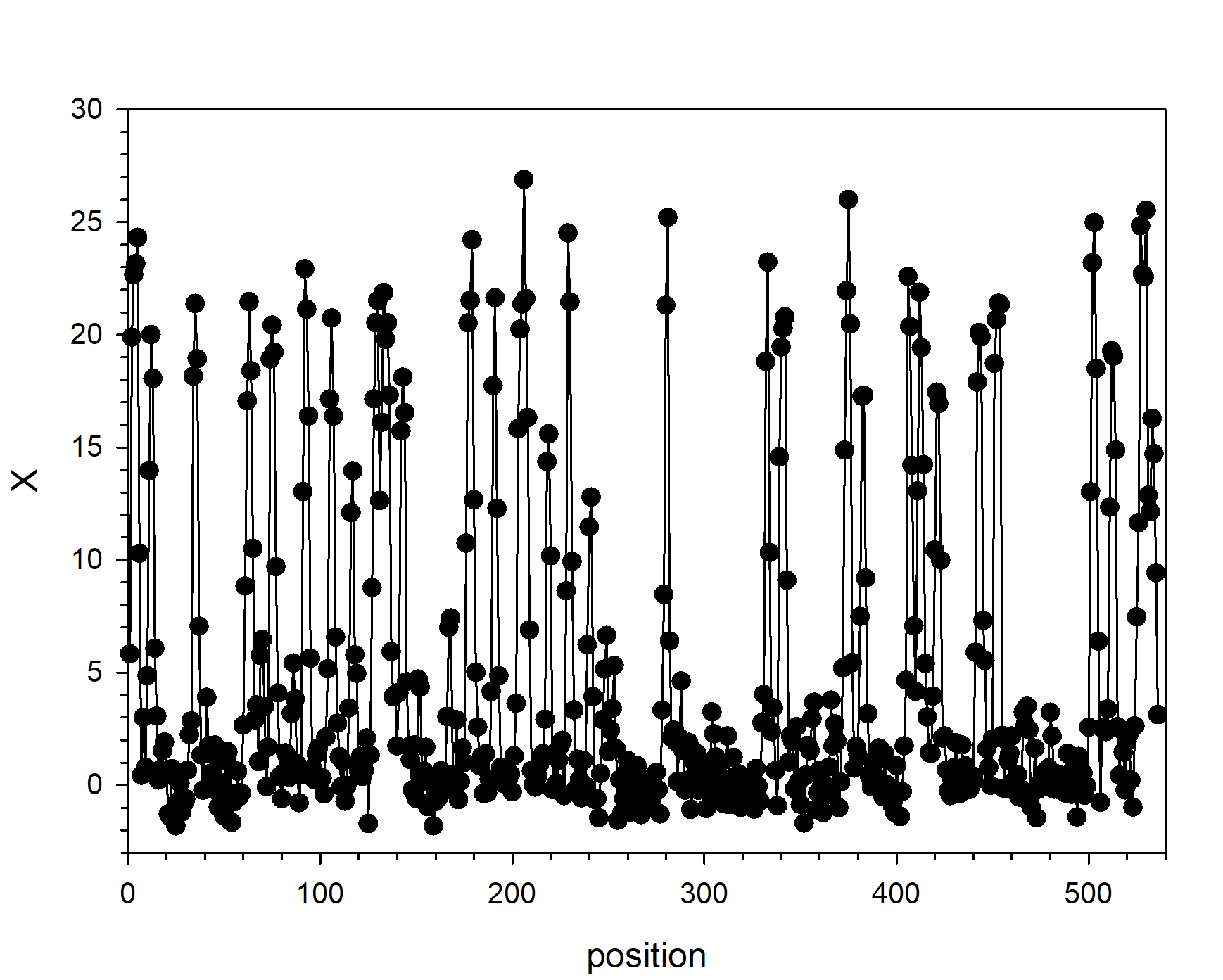

Supplement: Supplementary file 1 [file ijms-25-04441-s001.zip › ijms-2902088-supplementary/supplement/s1/fig2_5.jpg]

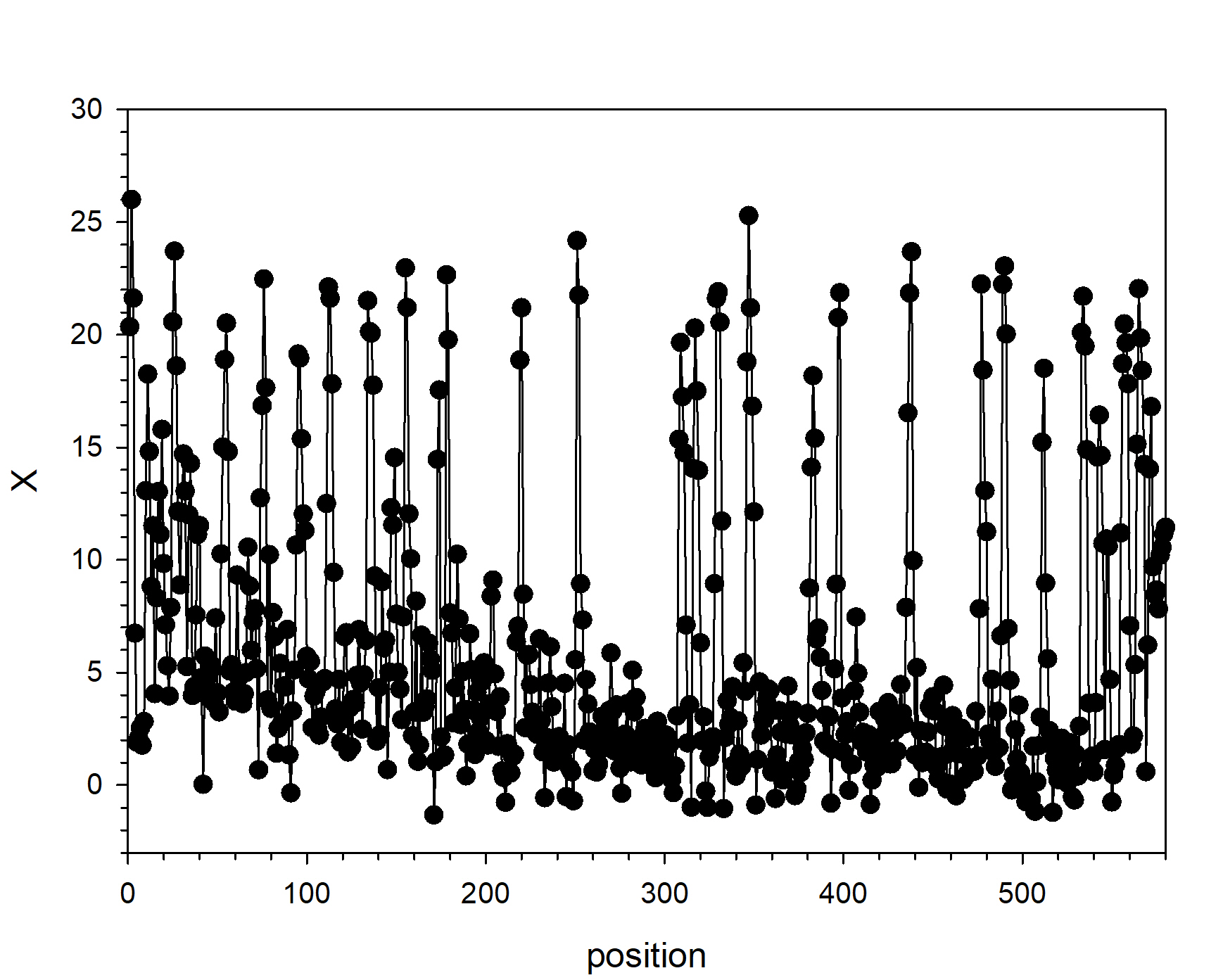

Supplement: Supplementary file 1 [file ijms-25-04441-s001.zip › ijms-2902088-supplementary/supplement/s1/fig2_6.jpg]

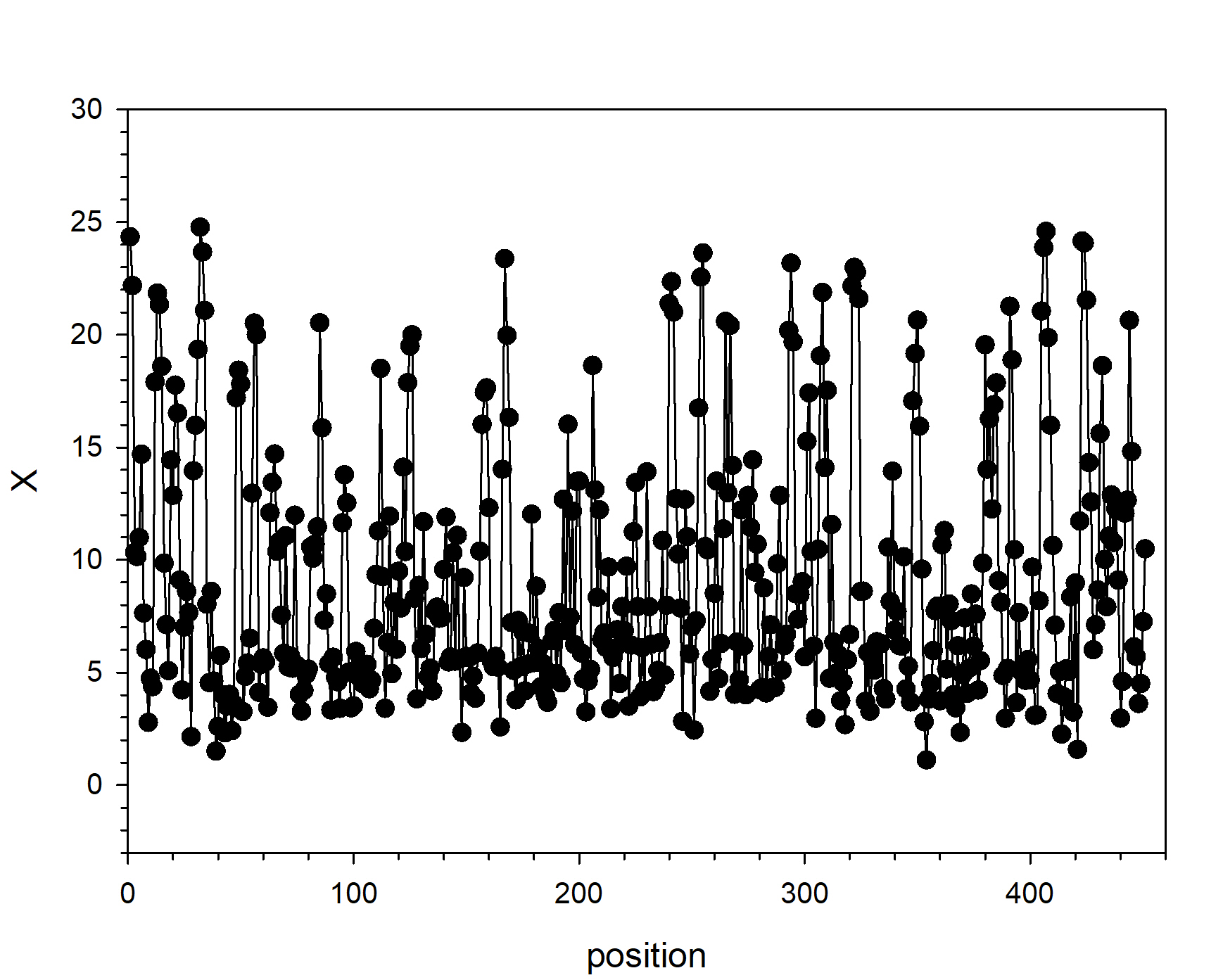

Supplement: Supplementary file 1 [file ijms-25-04441-s001.zip › ijms-2902088-supplementary/supplement/s1/fig2_7.jpg]

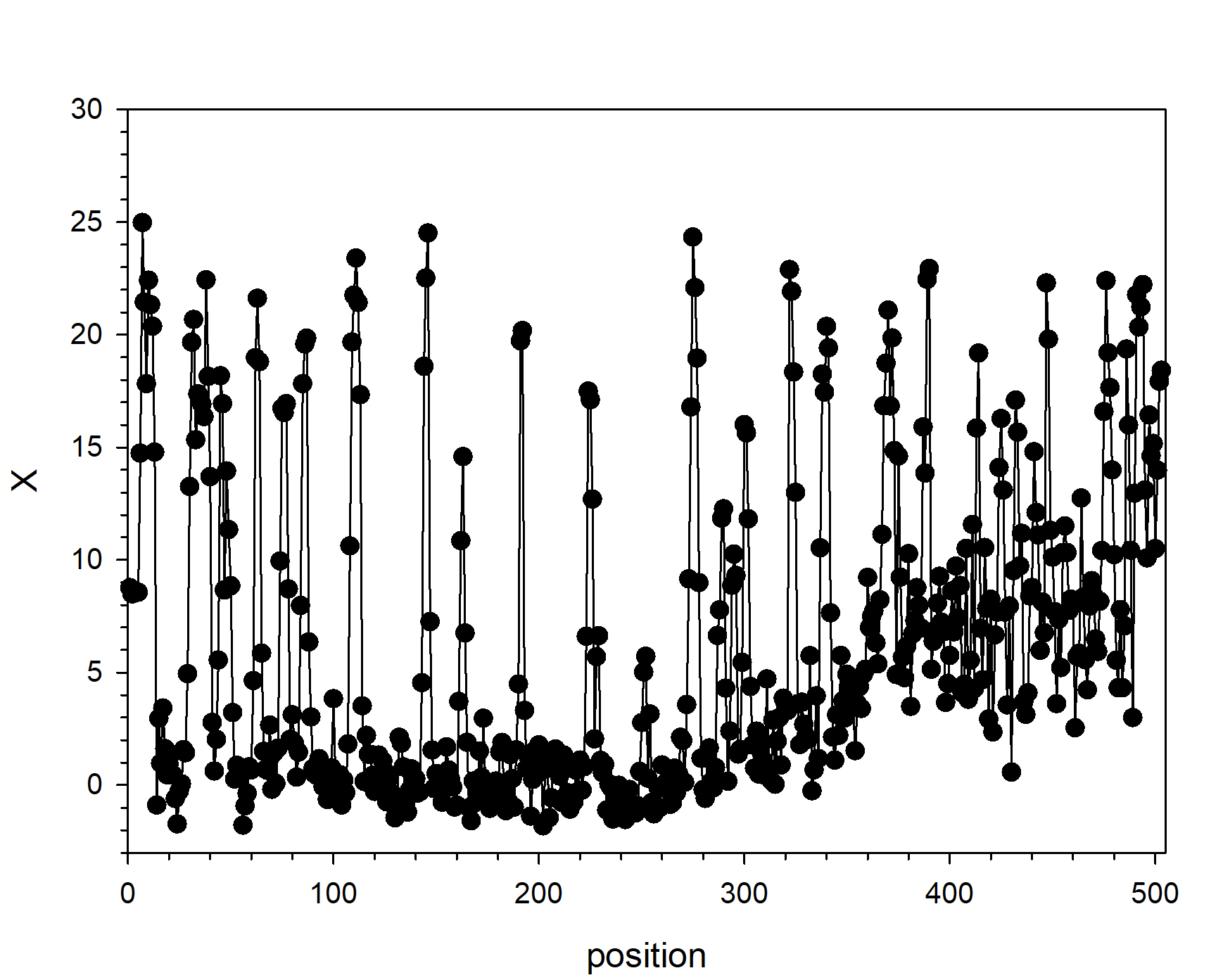

Supplement: Supplementary file 1 [file ijms-25-04441-s001.zip › ijms-2902088-supplementary/supplement/s1/fig2_8.jpg]

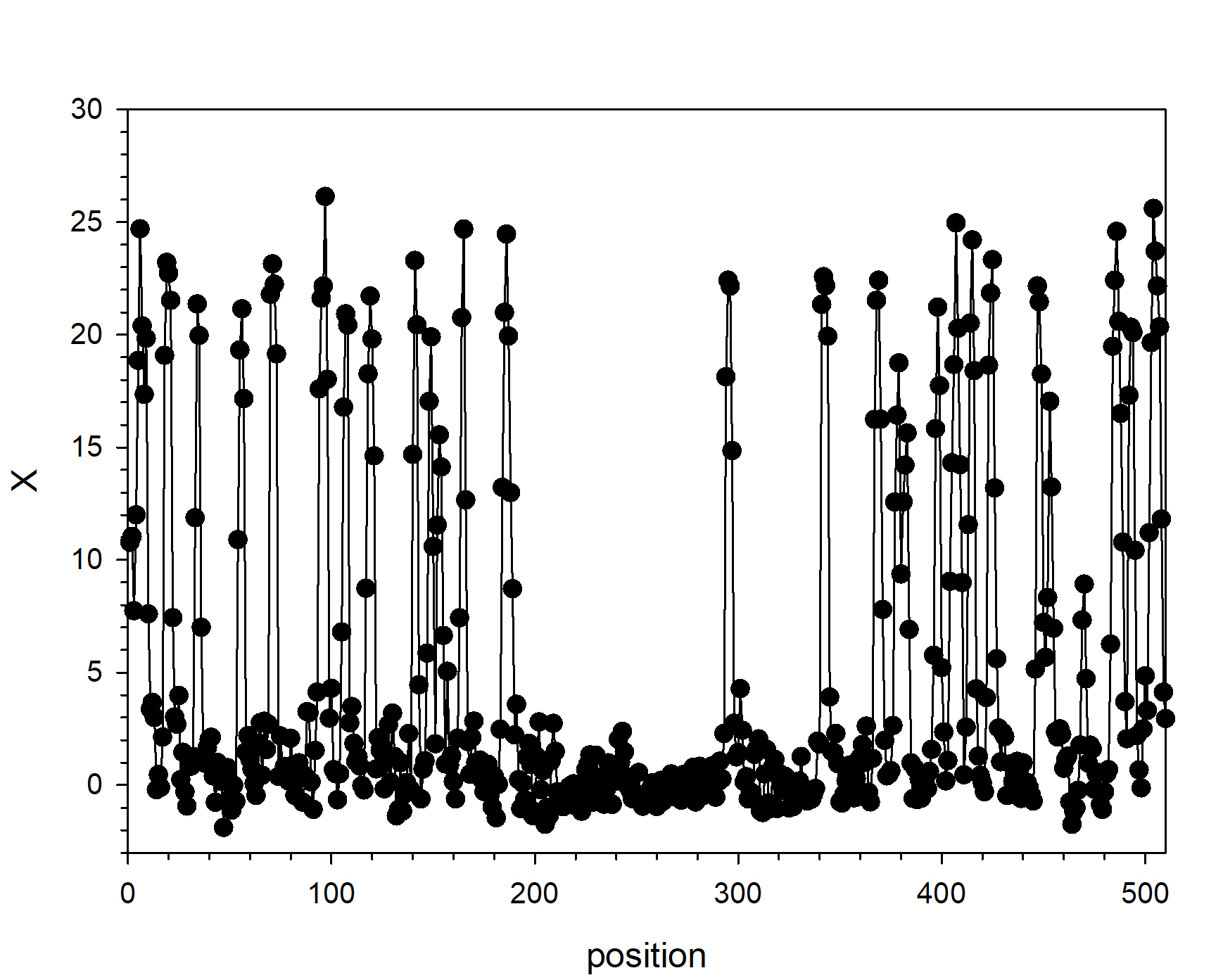

Supplement: Supplementary file 1 [file ijms-25-04441-s001.zip › ijms-2902088-supplementary/supplement/s1/fig2_9.jpg]

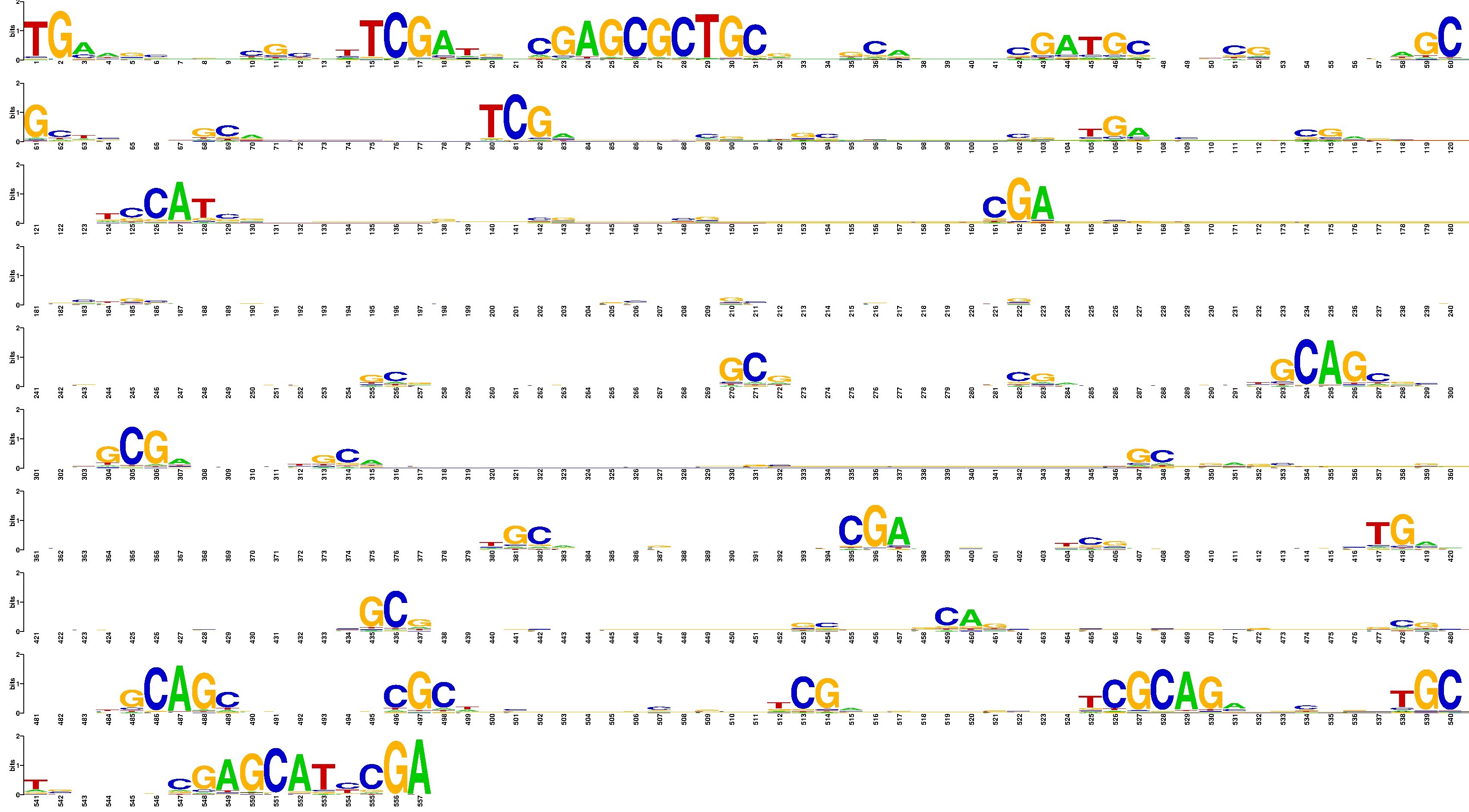

Supplement: Supplementary file 1 [file ijms-25-04441-s001.zip › ijms-2902088-supplementary/supplement/s1/fig4_1.jpg]

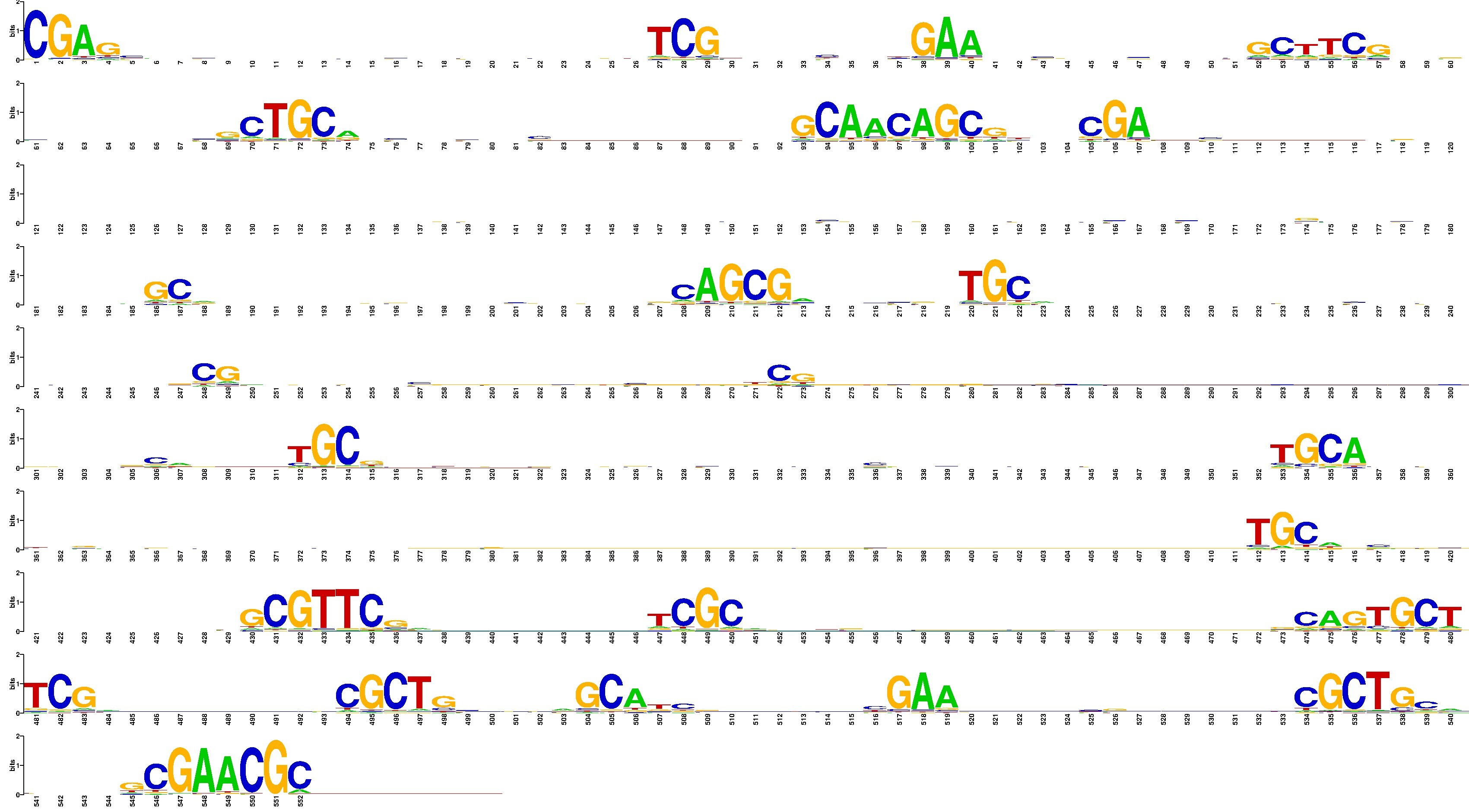

Supplement: Supplementary file 1 [file ijms-25-04441-s001.zip › ijms-2902088-supplementary/supplement/s1/fig4_10.jpg]

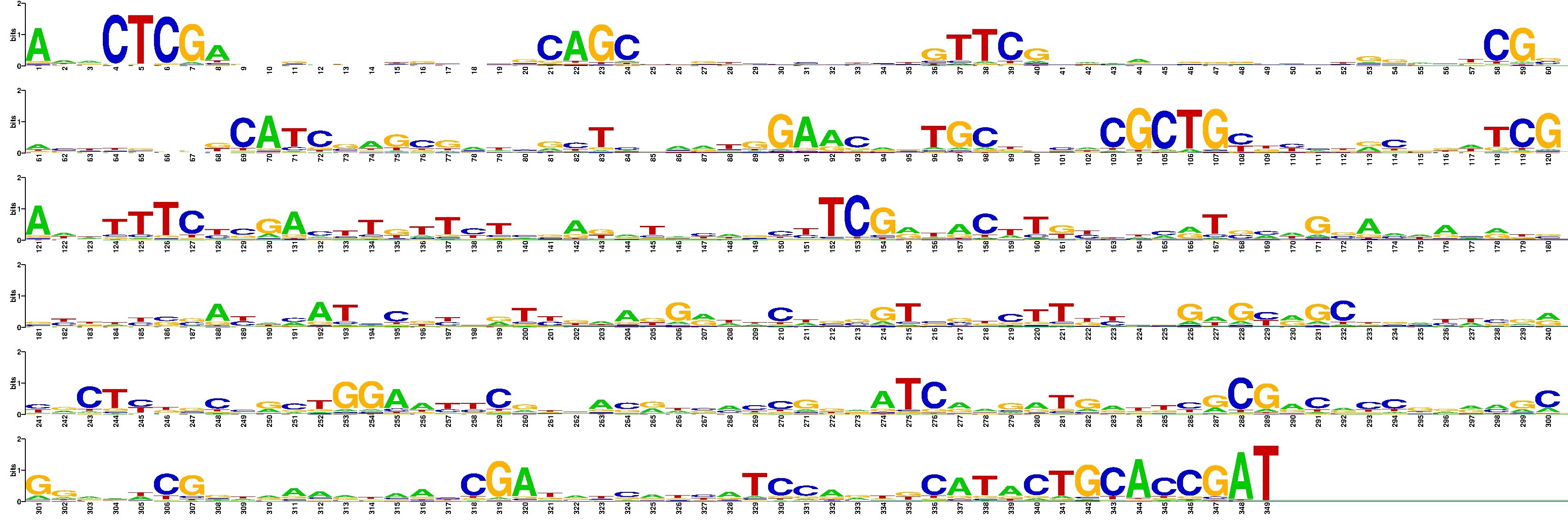

Supplement: Supplementary file 1 [file ijms-25-04441-s001.zip › ijms-2902088-supplementary/supplement/s1/fig4_11.jpg]

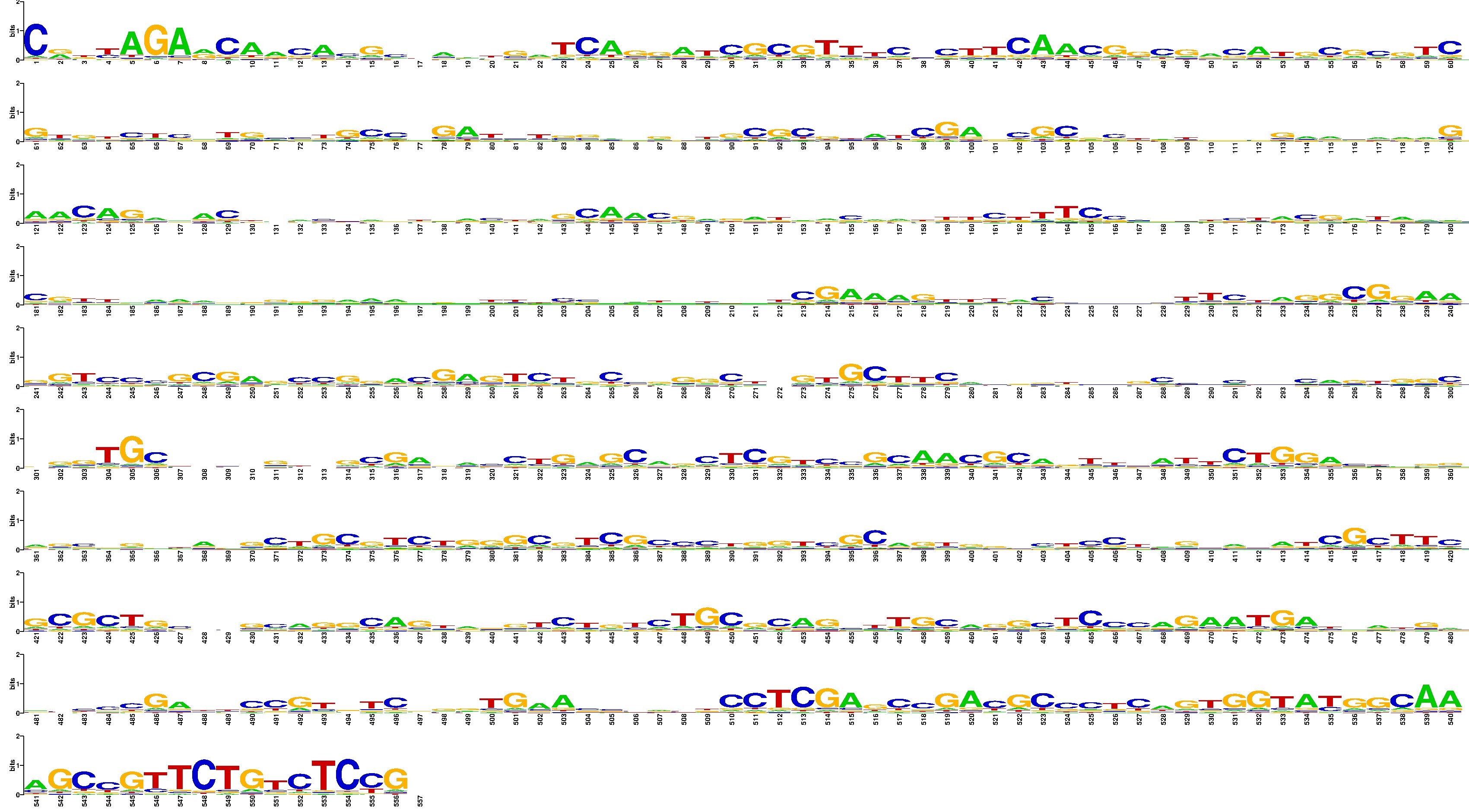

Supplement: Supplementary file 1 [file ijms-25-04441-s001.zip › ijms-2902088-supplementary/supplement/s1/fig4_12.jpg]

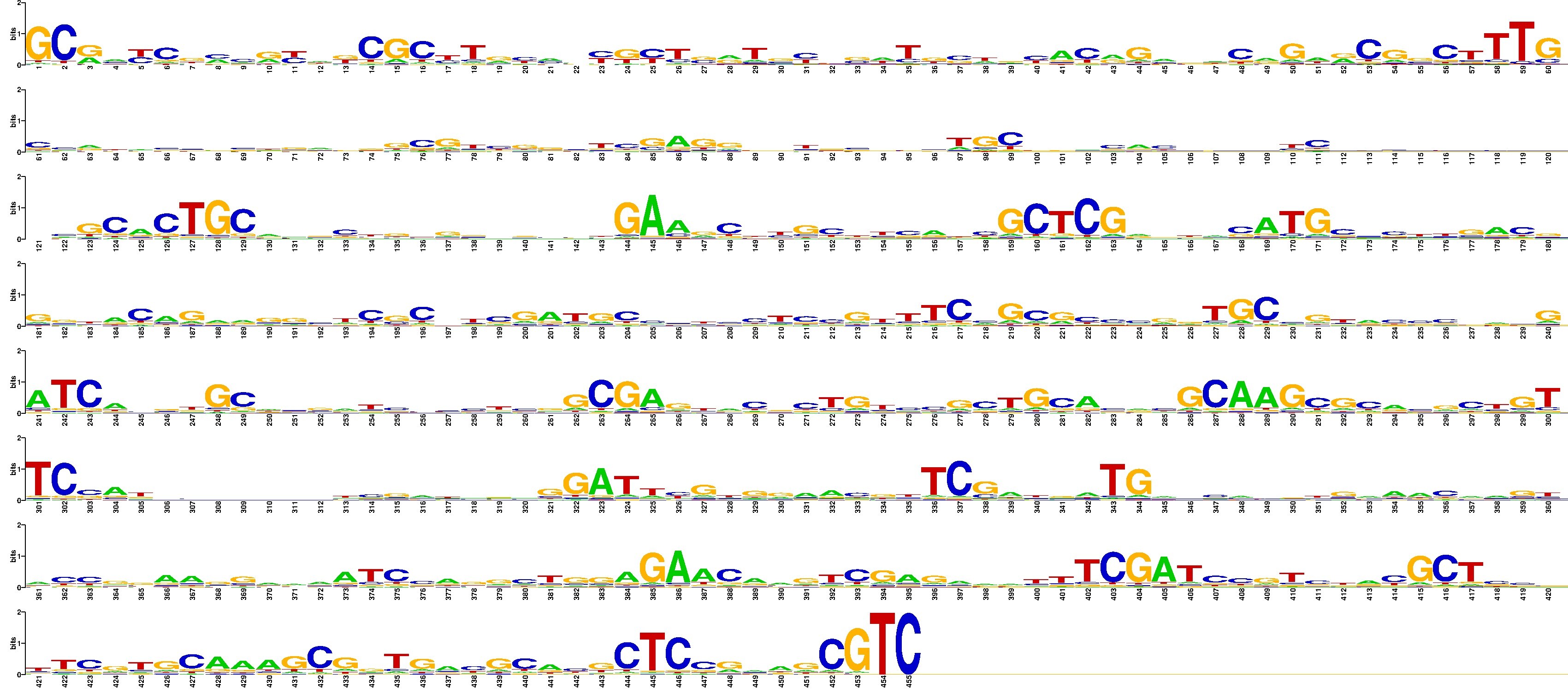

Supplement: Supplementary file 1 [file ijms-25-04441-s001.zip › ijms-2902088-supplementary/supplement/s1/fig4_13.jpg]

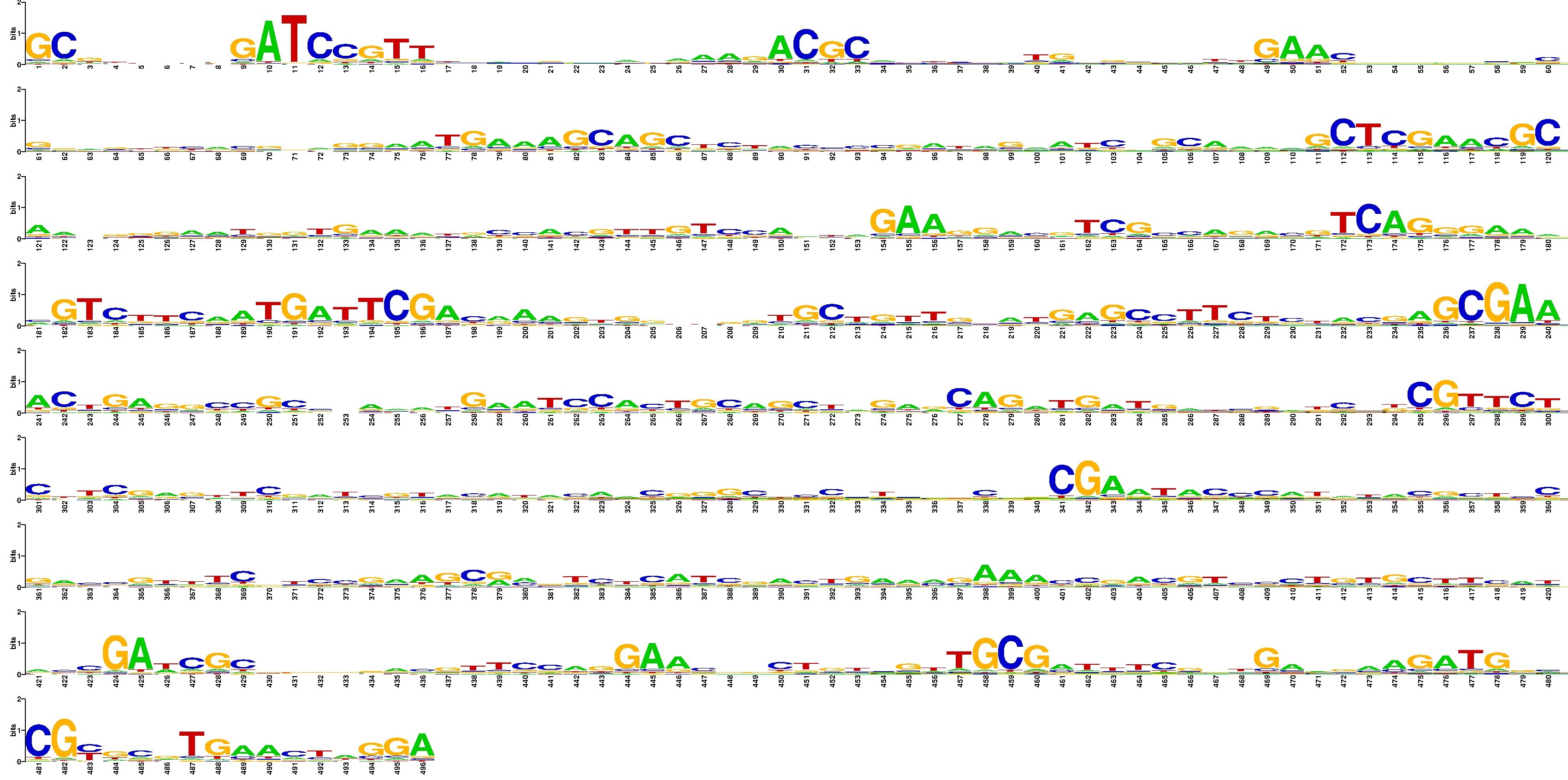

Supplement: Supplementary file 1 [file ijms-25-04441-s001.zip › ijms-2902088-supplementary/supplement/s1/fig4_14.jpg]

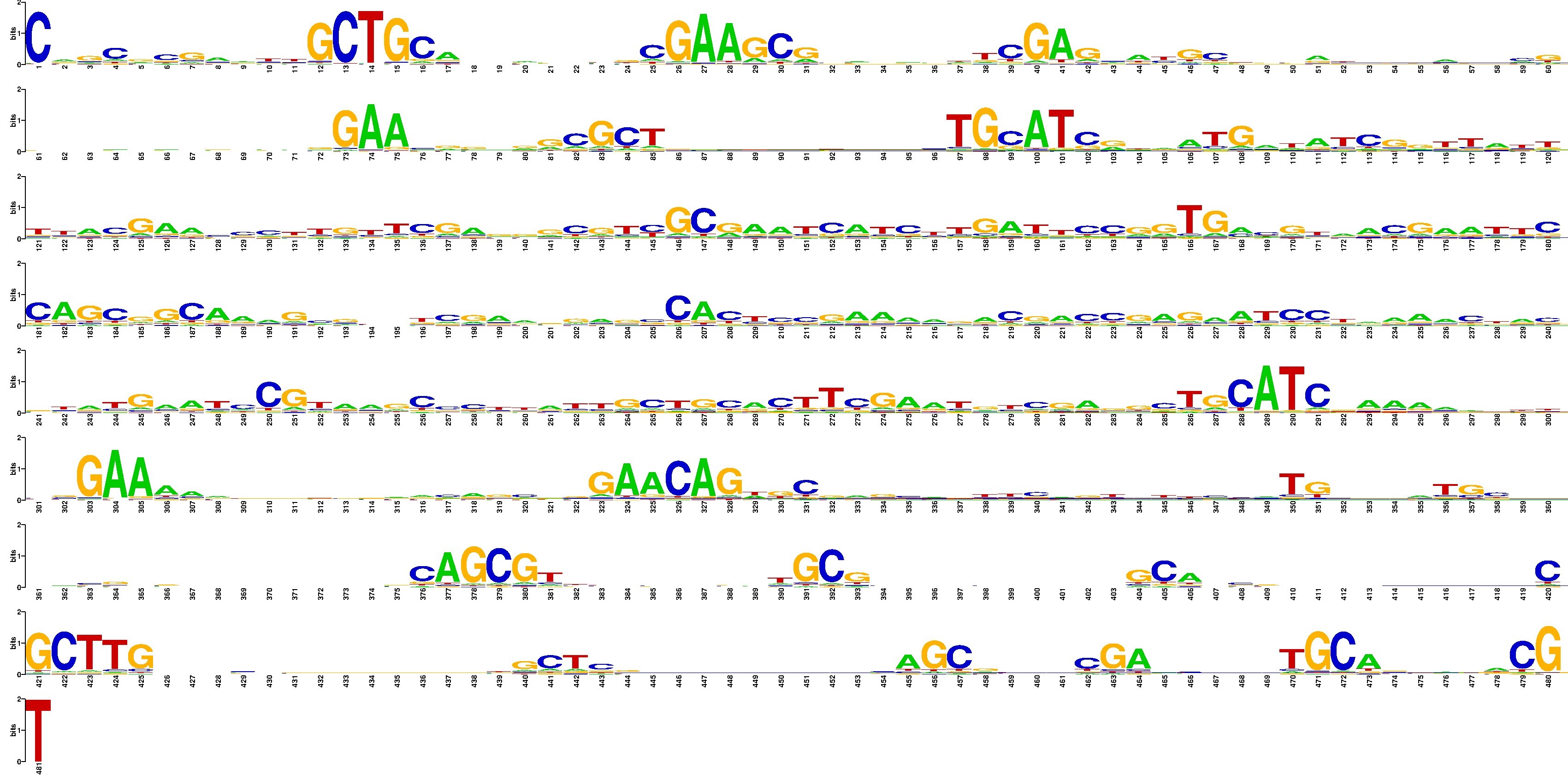

Supplement: Supplementary file 1 [file ijms-25-04441-s001.zip › ijms-2902088-supplementary/supplement/s1/fig4_15.jpg]

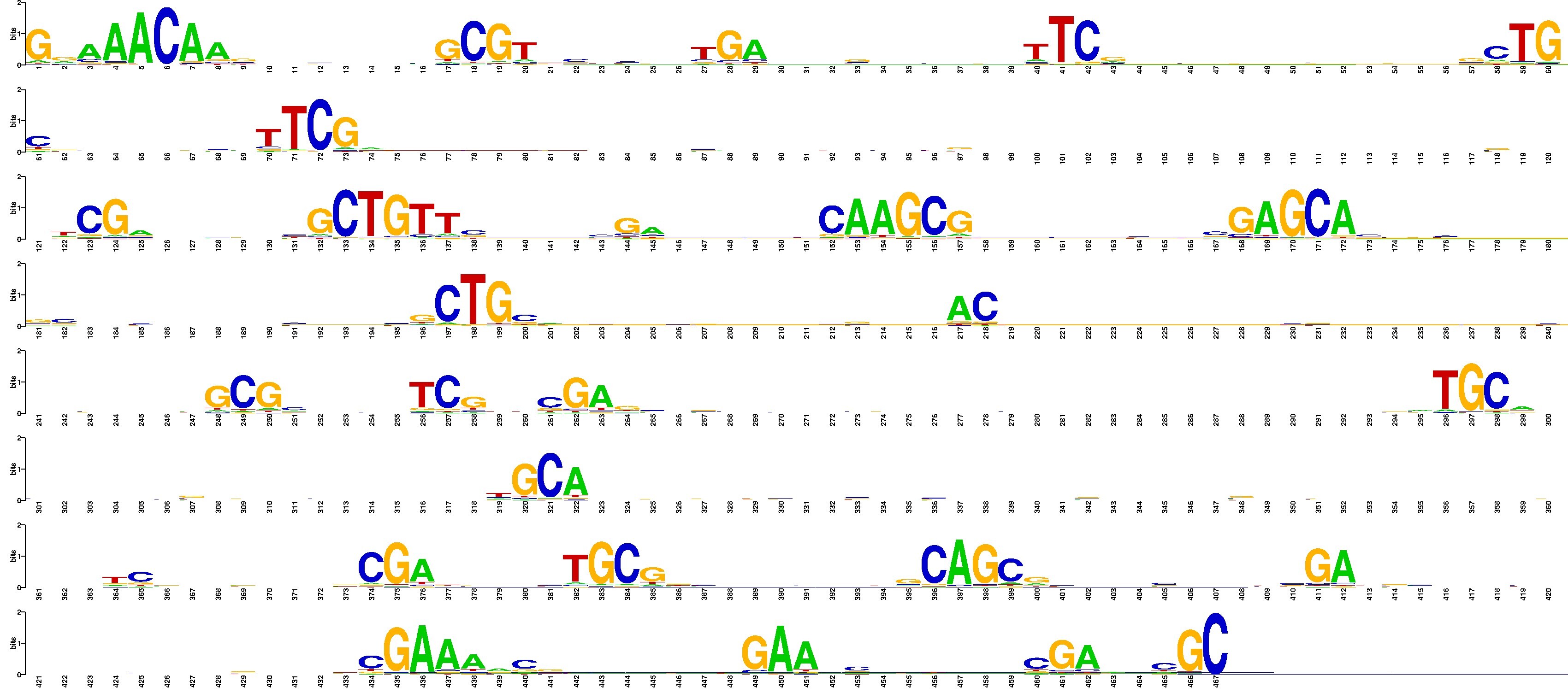

Supplement: Supplementary file 1 [file ijms-25-04441-s001.zip › ijms-2902088-supplementary/supplement/s1/fig4_16.jpg]

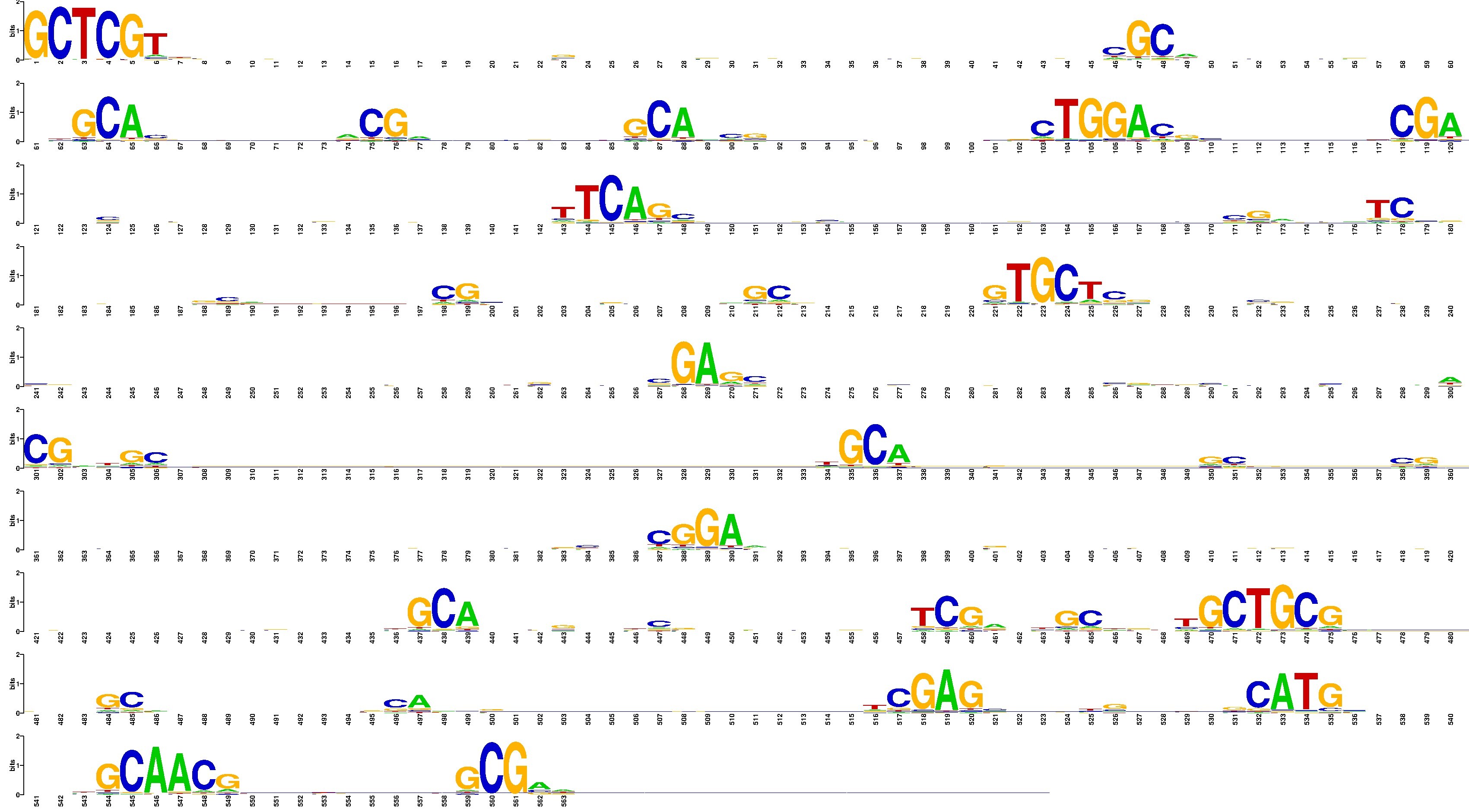

Supplement: Supplementary file 1 [file ijms-25-04441-s001.zip › ijms-2902088-supplementary/supplement/s1/fig4_17.jpg]

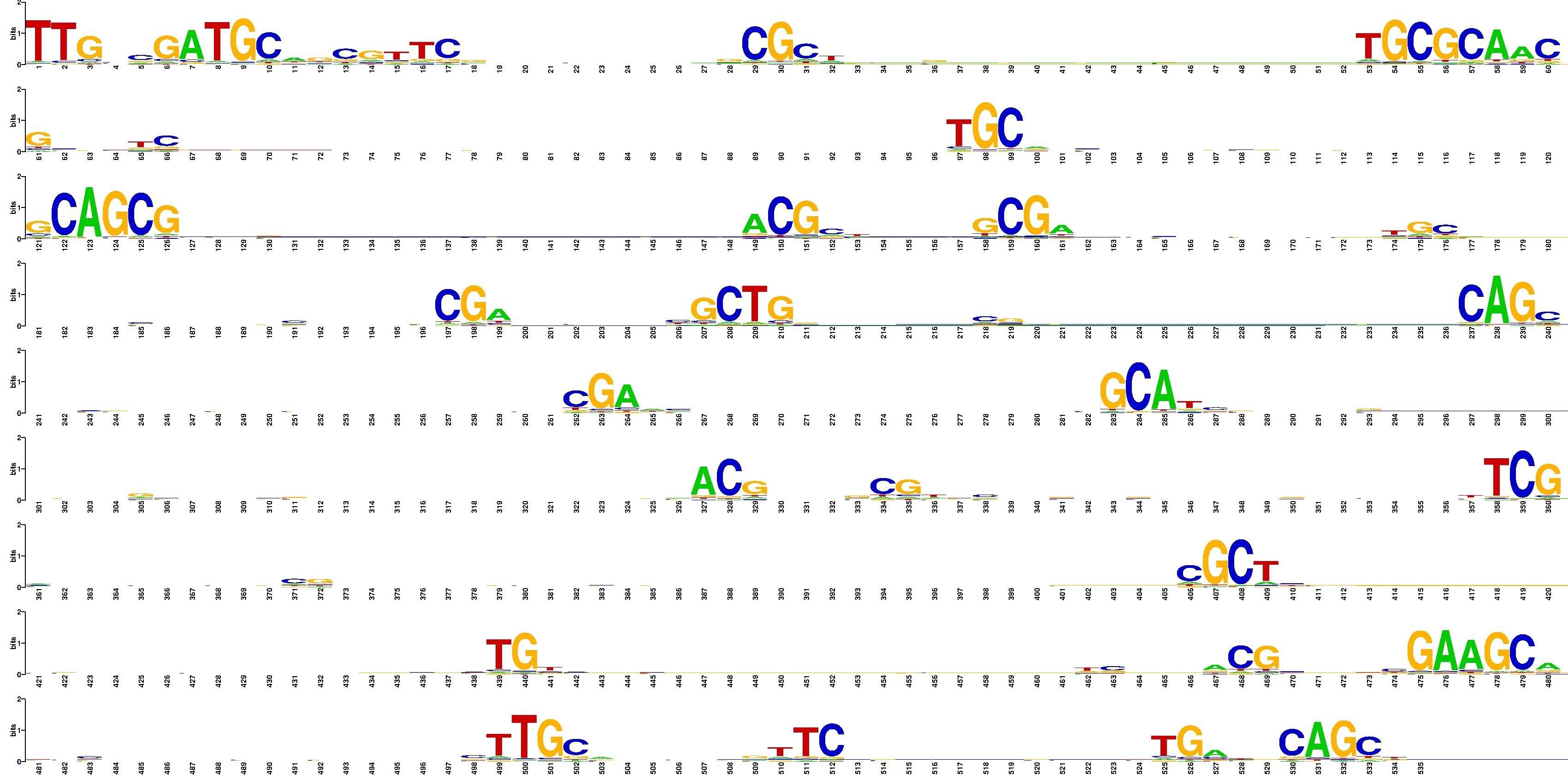

Supplement: Supplementary file 1 [file ijms-25-04441-s001.zip › ijms-2902088-supplementary/supplement/s1/fig4_18.jpg]

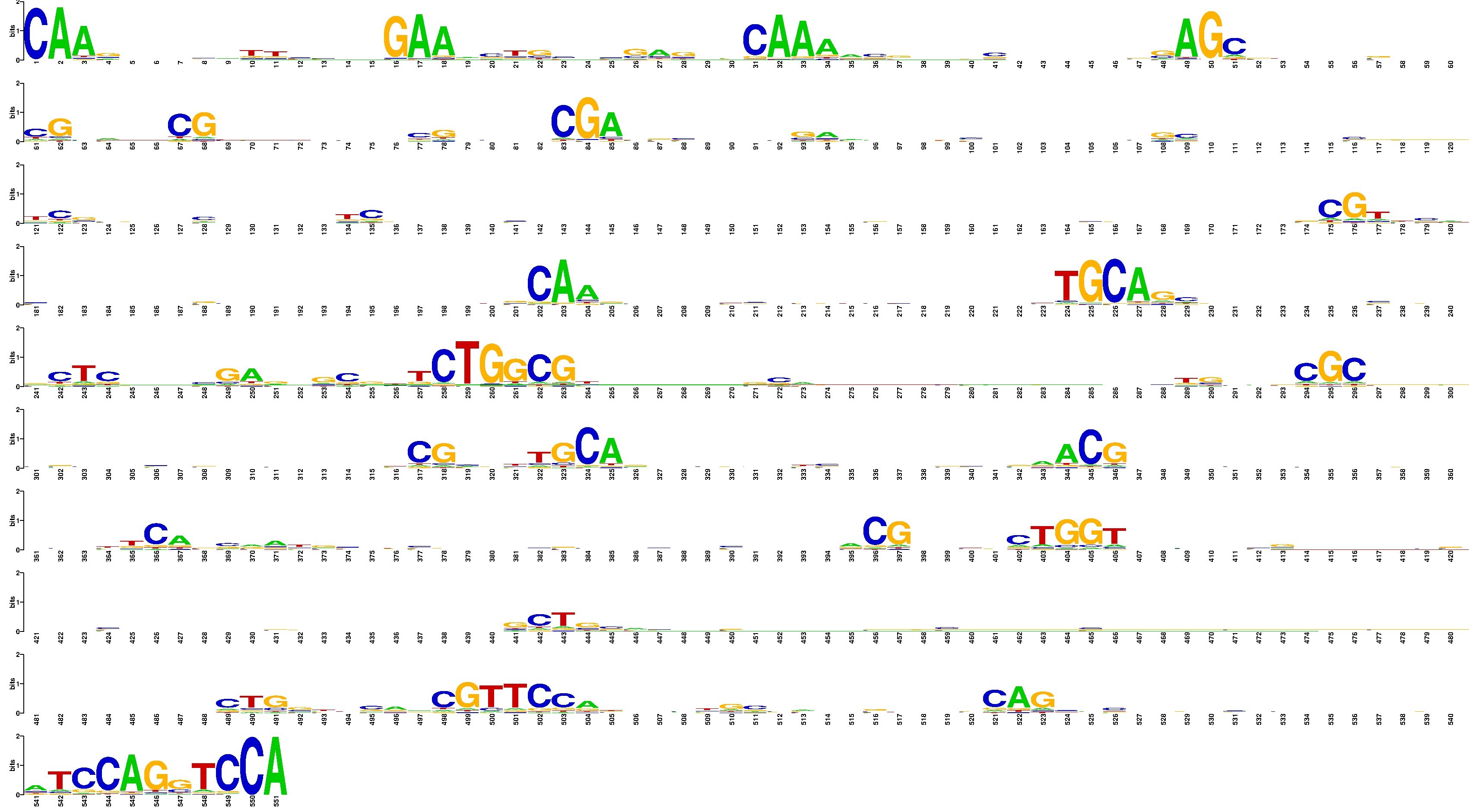

Supplement: Supplementary file 1 [file ijms-25-04441-s001.zip › ijms-2902088-supplementary/supplement/s1/fig4_19.jpg]

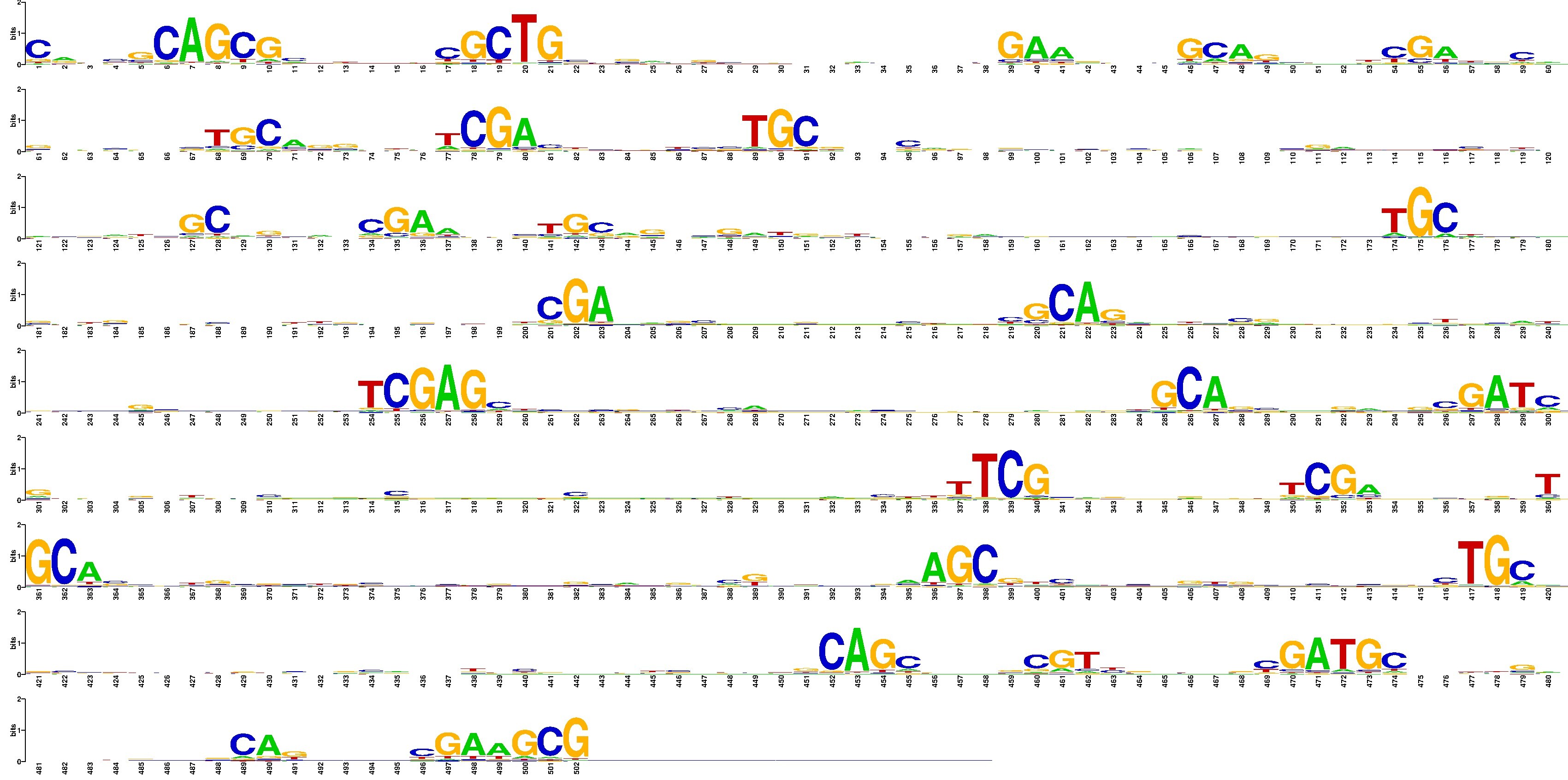

Supplement: Supplementary file 1 [file ijms-25-04441-s001.zip › ijms-2902088-supplementary/supplement/s1/fig4_2.jpg]

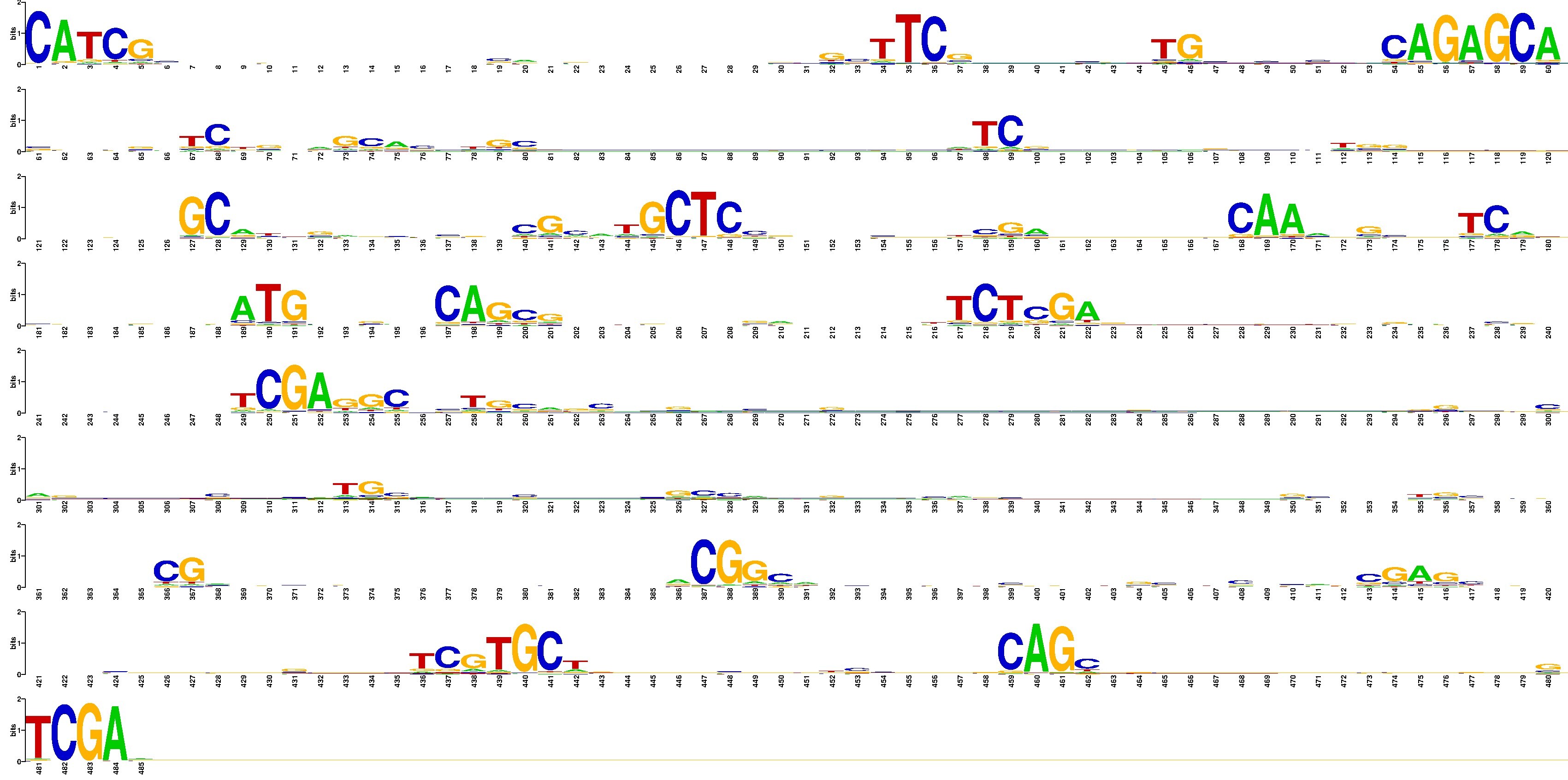

Supplement: Supplementary file 1 [file ijms-25-04441-s001.zip › ijms-2902088-supplementary/supplement/s1/fig4_20.jpg]

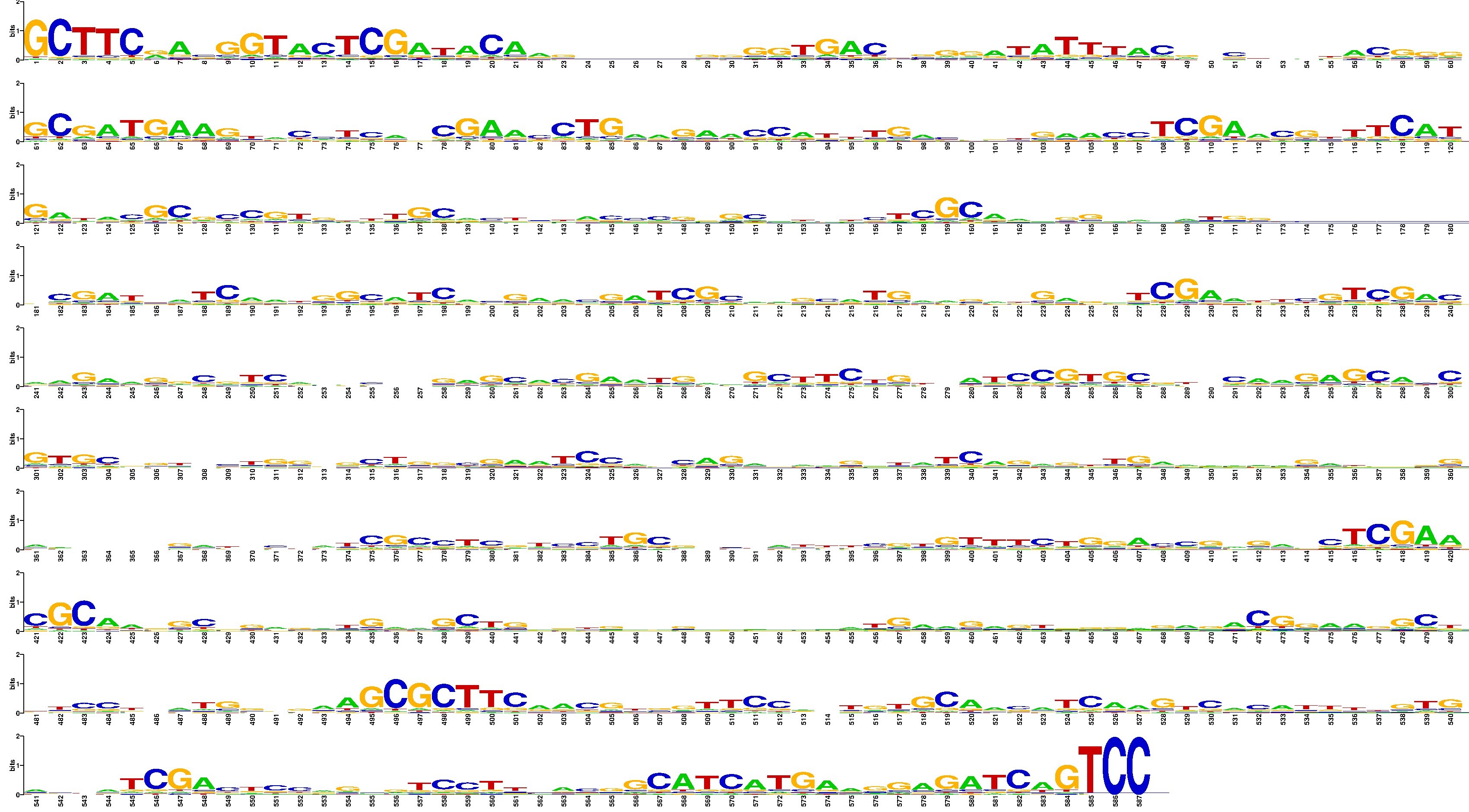

Supplement: Supplementary file 1 [file ijms-25-04441-s001.zip › ijms-2902088-supplementary/supplement/s1/fig4_3.jpg]

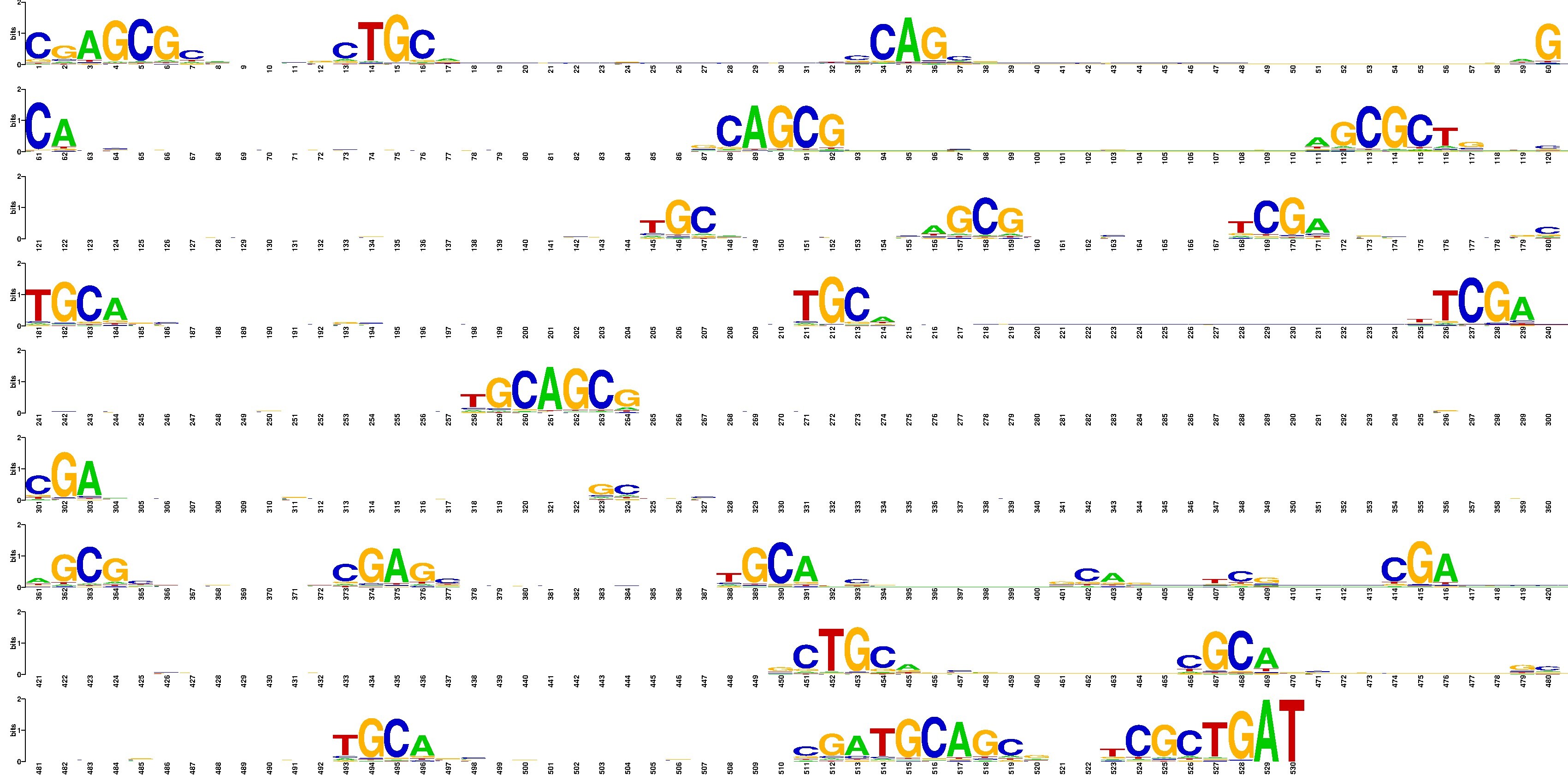

Supplement: Supplementary file 1 [file ijms-25-04441-s001.zip › ijms-2902088-supplementary/supplement/s1/fig4_4.jpg]

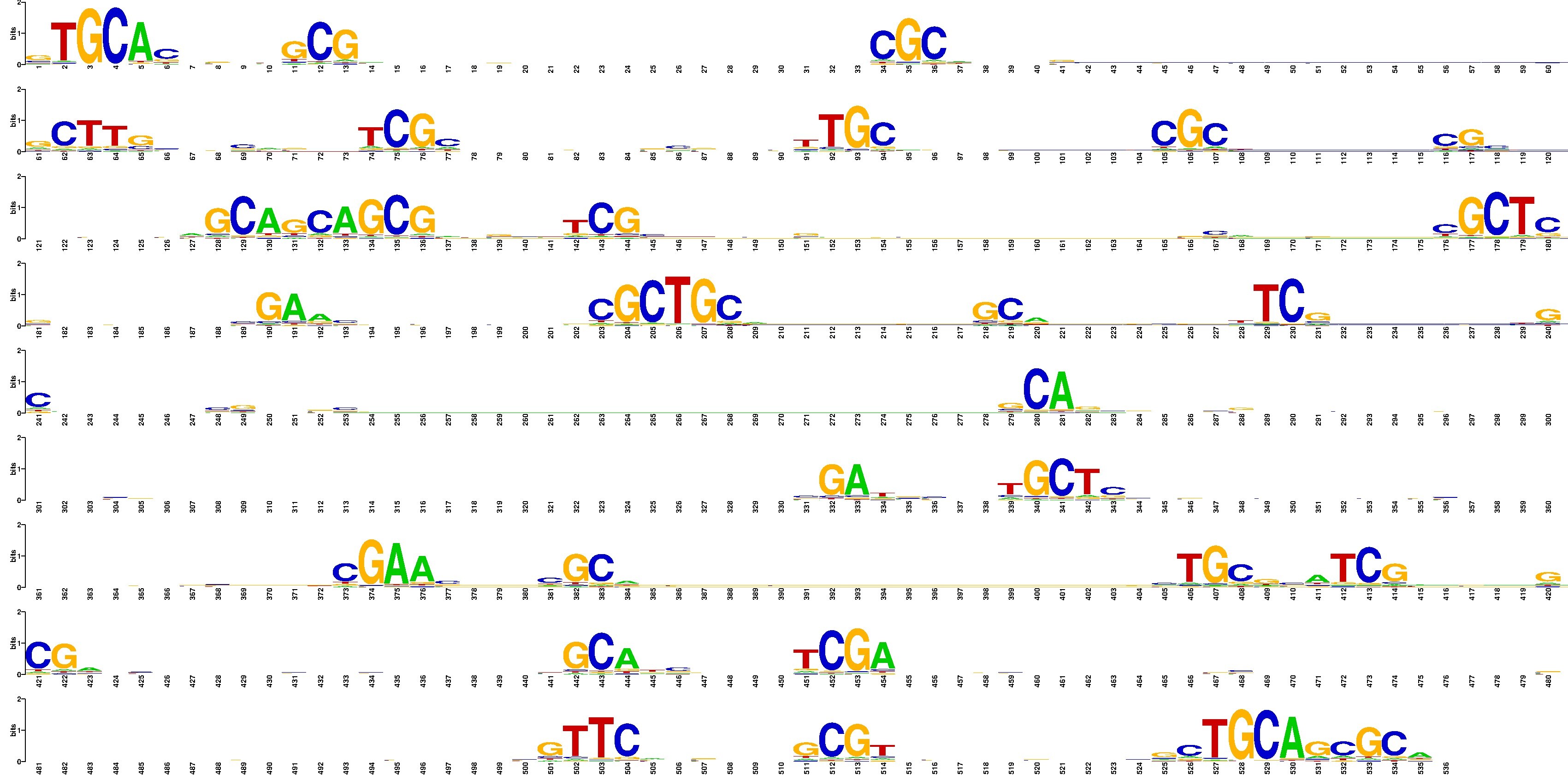

Supplement: Supplementary file 1 [file ijms-25-04441-s001.zip › ijms-2902088-supplementary/supplement/s1/fig4_5.jpg]

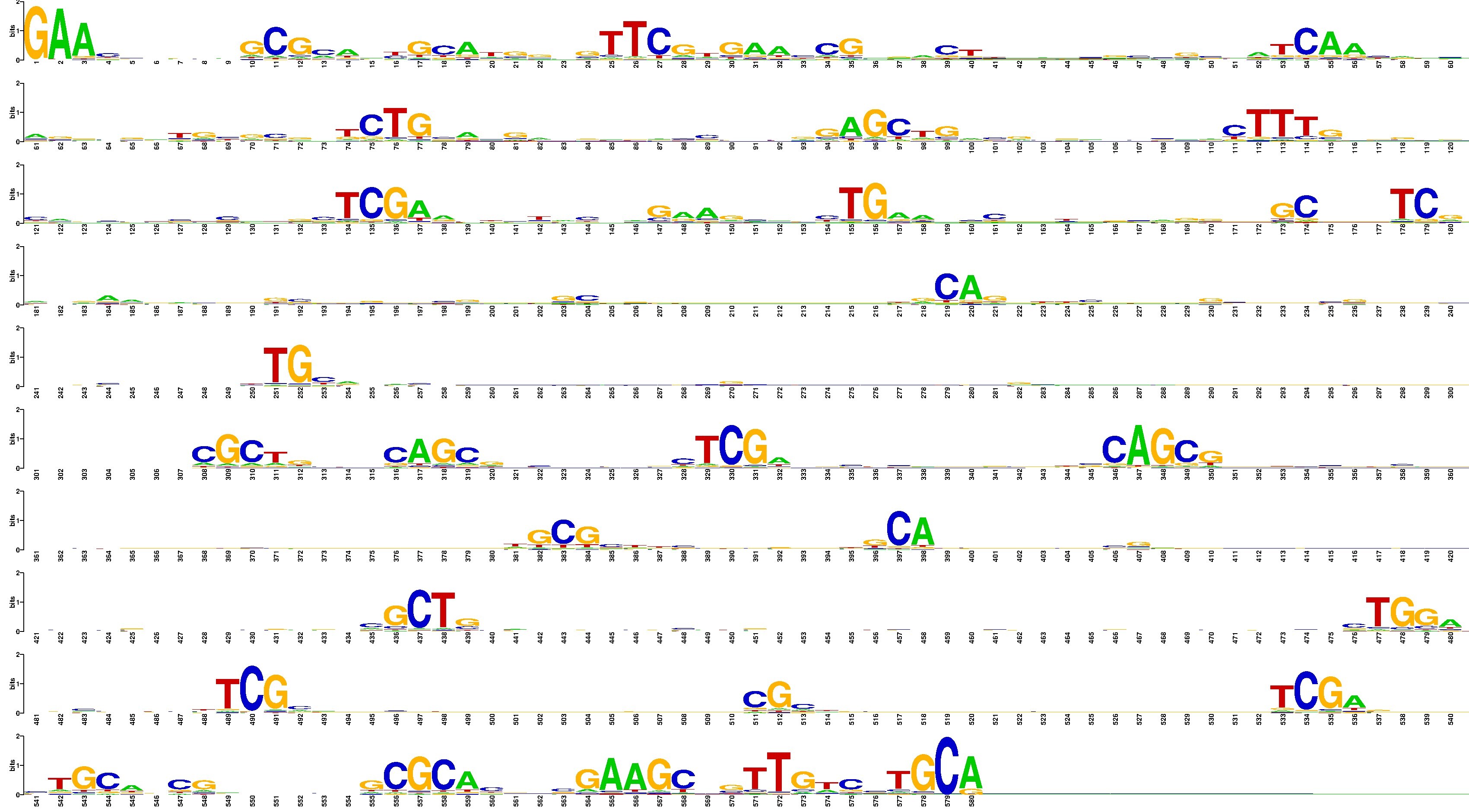

Supplement: Supplementary file 1 [file ijms-25-04441-s001.zip › ijms-2902088-supplementary/supplement/s1/fig4_6.jpg]

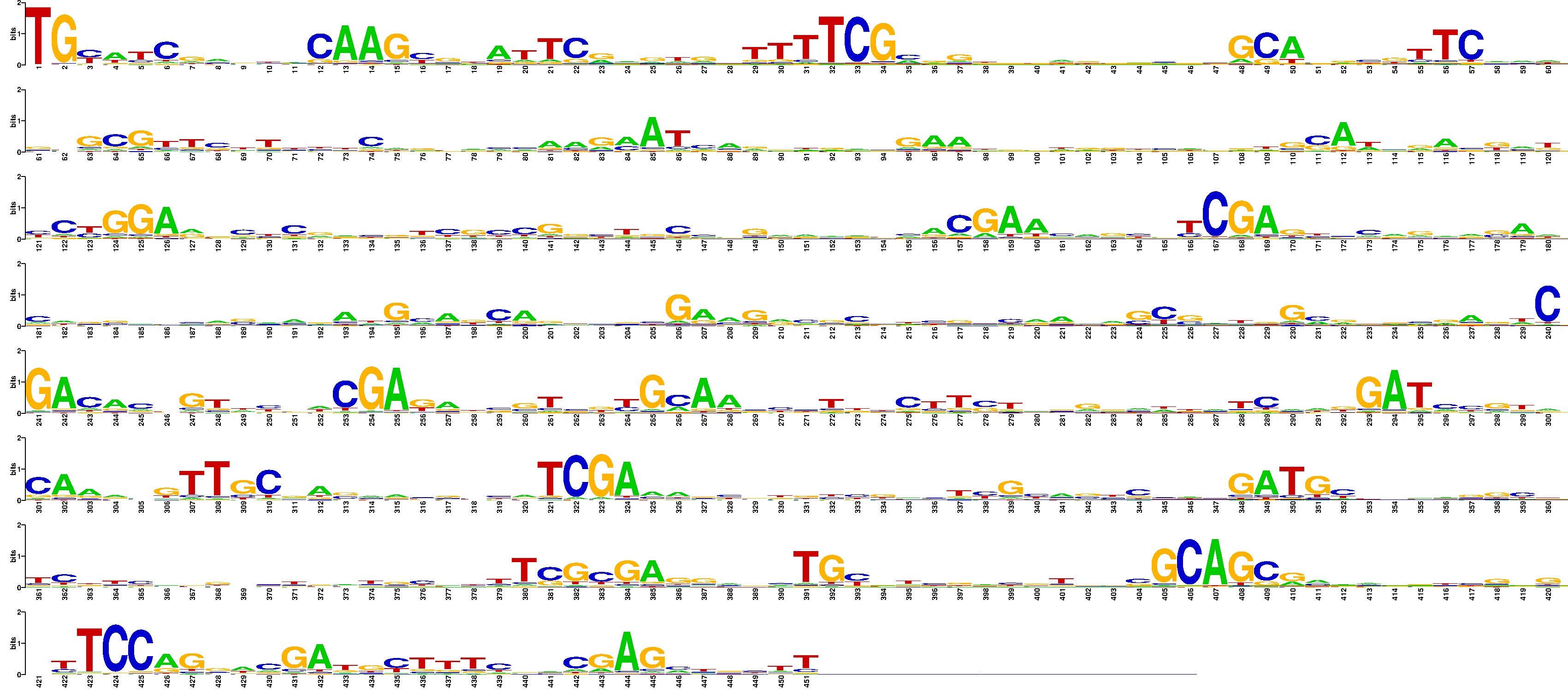

Supplement: Supplementary file 1 [file ijms-25-04441-s001.zip › ijms-2902088-supplementary/supplement/s1/fig4_7.jpg]

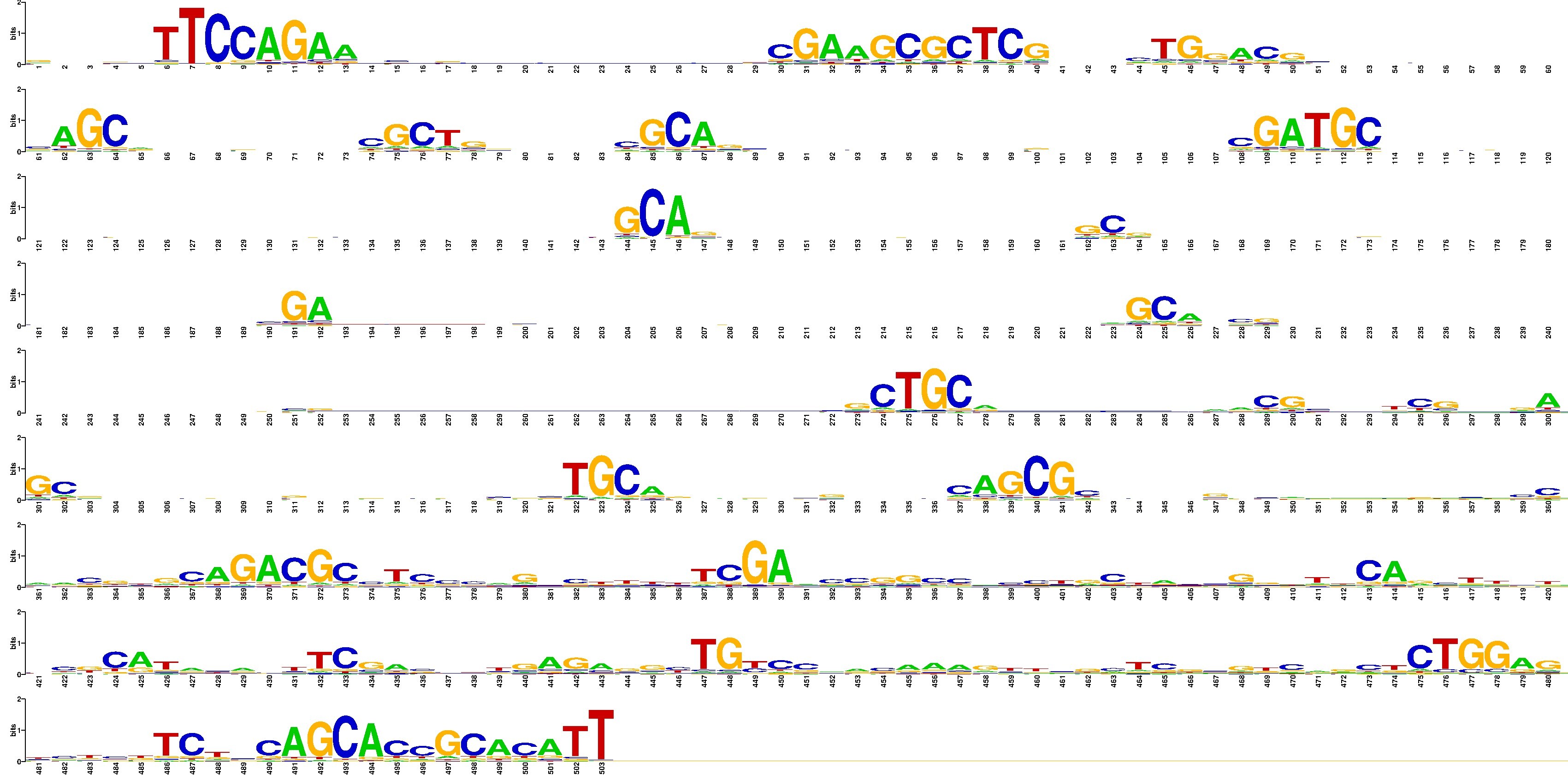

Supplement: Supplementary file 1 [file ijms-25-04441-s001.zip › ijms-2902088-supplementary/supplement/s1/fig4_8.jpg]

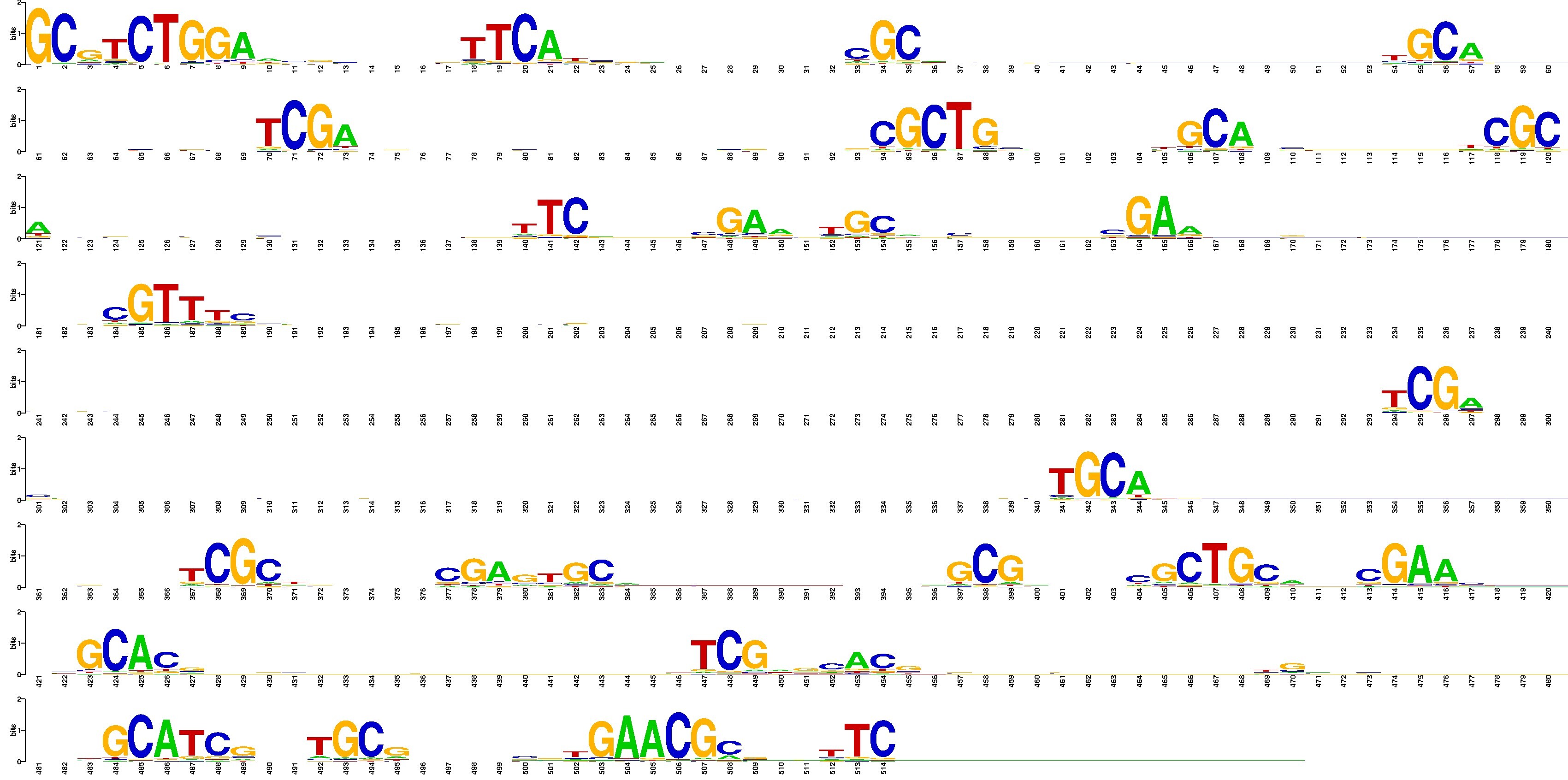

Supplement: Supplementary file 1 [file ijms-25-04441-s001.zip › ijms-2902088-supplementary/supplement/s1/fig4_9.jpg]
